# Systems biology markup language (SBML) level 3 package: multistate, multicomponent and multicompartment species, version 1, release 2

**DOI:** 10.1515/jib-2020-0015

**Published:** 2020-07-06

**Authors:** Fengkai Zhang, Lucian P. Smith, Michael L. Blinov, James Faeder, William S. Hlavacek, Jose Juan Tapia, Sarah M. Keating, Nicolas Rodriguez, Andreas Dräger, Leonard A. Harris, Andrew Finney, Bin Hu, Michael Hucka, Martin Meier-Schellersheim

**Affiliations:** NIAID/NIH, Bethesda, USA; University of Washington, Seattle, USA; UConn Health, Farmington, USA; University of Pittsburgh, Pittsburgh, USA; Los Alamos National Laboratory, Los Alamos, USA; European Bioinformatics Institute, Cambridge, UK; Babraham Institute, Cambridge, UK; Computational Systems Biology of Infection and Antimicrobial-Resistant Pathogens, Institute for Bioinformatics and Medical Informatics (IBMI), University of Tübingen, Tübingen, Germany; Vanderbilt University School of Medicine, Nashville, USA; Ansys UK Ltd, Abingdon, UK; California Institute of Technology, Pasadena, USA; Department of Computer Science, University of Tübingen, Tübingen, Germany; German Center for Infection Research (DZIF), partner site Tübingen, Germany

**Keywords:** rule-based modeling, specification, standard, systems biology

## Abstract

Rule-based modeling is an approach that permits constructing reaction networks based on the specification of rules for molecular interactions and transformations. These rules can encompass details such as the interacting sub-molecular domains and the states and binding status of the involved components. Conceptually, fine-grained spatial information such as locations can also be provided. Through “wildcards” representing component states, entire families of molecule complexes sharing certain properties can be specified as patterns. This can significantly simplify the definition of models involving species with multiple components, multiple states, and multiple compartments. The systems biology markup language (SBML) Level 3 Multi Package Version 1 extends the SBML Level 3 Version 1 core with the “type” concept in the Species and Compartment classes. Therefore, reaction rules may contain species that can be patterns and exist in multiple locations. Multiple software tools such as Simmune and BioNetGen support this standard that thus also becomes a medium for exchanging rule-based models. This document provides the specification for Release 2 of Version 1 of the SBML Level 3 Multi package. No design changes have been made to the description of models between Release 1 and Release 2; changes are restricted to the correction of errata and the addition of clarifications.

***Corresponding author: Fengkai Zhang**, NIAID/NIH, Bethesda, USA, E-mail: zhangfen@nih.gov. https://orcid.org/0000-0001-7112-9328
**Lucian P. Smith:** University of Washington, Seattle, USA, E-mail: lpsmith@uw.edu. https://orcid.org/0000-0001-7002-6386
**Michael L. Blinov:** UConn Health, Farmington, USA, E-mail: blinov@uchc.edu. https://orcid.org/0000-0002-9363-9705
**James Faeder and Jose Juan Tapia:** University of Pittsburgh, Pittsburgh, USA, E-mail: faeder@pitt.edu (J. Faeder), jjtapia@gmail.com (J. Juan Tapia). https://orcid.org/0000-0001-8127-609X (J. Faeder) . https://orcid.org/0000-0002-8392-6022 (J. Juan Tapia)
**William S. Hlavacek and Bin Hu:** Los Alamos National Laboratory, Los Alamos, USA, E-mail: wish@lanl.gov (W.S. Hlavacek), bhu@lanl.gov (B. Hu). https://orcid.org/0000-0003-4383-8711 (W.S. Hlavacek) . https://orcid.org/0000-0002-3775-387X (B. Hu)
**Sarah M. Keating:** European Bioinformatics Institute, Cambridge, UK, E-mail: keating.sm@googlemail.com. https://orcid.org/0000-0002-3356-3542
**Nicolas Rodriguez:** Babraham Institute, Cambridge, UK, E-mail: nicolas.rodriguez@babraham.ac.uk. https://orcid.org/0000-0002-9290-7894
**Andreas Dräger:** Computational Systems Biology of Infection and Antimicrobial-Resistant Pathogens, Institute for Bioinformatics and Medical Informatics (IBMI), University of Tübingen, Tübingen, Germany, E-mail: andreas.draeger@uni-tuebingen.de. https://orcid.org/0000-0002-1240-5553
**Leonard A. Harris:** Vanderbilt University School of Medicine, Nashville, USA, E-mail: l.harris@vanderbilt.edu. https://orcid.org/0000-0003-2112-6940
**Andrew Finney:** Ansys UK Ltd, Abingdon, UK; Department of Computer Science, University of Tübingen, Tübingen, Germany; and German Center for Infection Research (DZIF), partner site Tübingen, Germany, E-mail: andrewmartinfinney@gmail.com
**Michael Hucka:** California Institute of Technology, Pasadena, USA, E-mail: mhucka@caltech.edu. https://orcid.org/0000-0001-9105-5960
**Martin Meier-Schellersheim:** NIAID/NIH, Bethesda, USA, E-mail: mms@niaid.nih.gov. https://orcid.org/0000-0002-8754-6377

## Multistate, Multicomponent and Multicompartment Species Package for SBML Level 3

Fengkai Zhang
zhangfen@niaid.nih.gov
NIAID/NIH
USA

Martin Meier-Schellersheim
mms@niaid.nih.gov
NIAID/NIH
USA

Version 1, Release 2

17 April 2020

The latest release, past releases, and other materials related to this specification are available at
http://sbml.org/Documents/Specifications/SBML_Level_3/Packages/multi

*This* release of the specification is available at
http://identifiers.org/combine.specifications/sbml.level-3.version-1.multi.version-1.release-2

### Contributors

Bastian R. Angermann
NIAID/NIH
USA

Frank Bergmann
California Institute of Technology
USA

Michael L. Blinov
UConn Health
USA

Andreas Dräger
Universtiy of Tuebingen
Germany

James Faeder
University of Pittsburgh School of Medicine
USA

Andrew Finney
University Hertfordshire
United Kingdom

Leonard A. Harris
University of Pittsburgh School of Medicine
USA

William S. Hlavacek
Los Alamos National Laboratory
USA

Stefan Hoops
Virginia Tech
USA

Bin Hu
Los Alamos National Laboratory
USA

Michael Hucka
California Institute of Technology
USA

Sarah M. Keating
European Bioinformatics Institute
United Kingdom

Nicolas Le Novère
Babraham Institute
United Kingdom

Anika Oellrich
European Bioinformatics Institute
United Kingdom

Nicolas Rodriguez
European Bioinformatics Institute
United Kingdom

Lucian P. Smith
University of Washington
USA

Jose Juan Tapia
University of Pittsburgh School of Medicine
USA

And all the people who contributed to the discussions on the sbml-multi mailing list.

## Contents

**1 Introduction** 5
- 1.1 Proposal and specifications . . . 5
- 1.2 Package dependencies . . . 5
- 1.3 Document conventions . . . 5

**2 Background and context** 6
- 2.1 Past work on this problem or similar topics . . . 6
- 2.2 Revision history . . . 7
  - 2.2.1 Release(s) . . . 7
  - 2.2.2 Release Candidates . . . 7
  - 2.2.3 Drafts . . . 8
  - 2.2.4 Revision history before draft version 1.0.1 . . . 8

**3 Package syntax and semantics** 9
- 3.1 Namespace URI and other declarations necessary for using this package . . . 9
- 3.2 Primitive data types . . . 9
  - 3.2.1 Type `BindingStatus` . . . 9
  - 3.2.2 Type `Relation` . . . 9
  - 3.2.3 Type `RepresentationType` . . . 10
- 3.3 The new and extended classes in the Multi Package . . . 10
- 3.4 Extended **Model** . . . 10
  - 3.4.1 **ListOfSpeciesTypes** . . . 11
- 3.5 Extended **Compartment** . . . 11
  - 3.5.1 The `isType` attribute . . . 11
  - 3.5.2 The `compartmentType` attribute . . . 11
  - 3.5.3 **ListOfCompartmentReferences** . . . 12
- 3.6 **CompartmentReference** . . . 12
  - 3.6.1 The `id` and `name` attributes . . . 12
  - 3.6.2 The `compartment` attribute . . . 12
- 3.7 The relationship of **Compartment**, **CompartmentReference** and **ListOfCompartmentReferences** . . . 12
- 3.8 **SpeciesType** . . . 12
  - 3.8.1 The `id` and `name` attributes . . . 13
  - 3.8.2 The `compartment` attribute . . . 13
  - 3.8.3 **ListOfSpeciesFeatureTypes** . . . 13
  - 3.8.4 **ListOfSpeciesTypeInstances** . . . 13
  - 3.8.5 **ListOfInSpeciesTypeBonds** . . . 14
  - 3.8.6 **ListOfSpeciesTypeComponentIndexes** . . . 14
  - 3.8.7 **BindingSiteSpeciesType** . . . 14
- 3.9 **SpeciesFeatureType** . . . 14
  - 3.9.1 The `id` and `name` attributes . . . 14
  - 3.9.2 The `occur` attribute . . . 14
  - 3.9.3 **ListOfPossibleSpeciesFeatureValues** . . . 14
- 3.10 **PossibleSpeciesFeatureValue** . . . 15
  - 3.10.1 The `id` and `name` attributes . . . 15
  - 3.10.2 The `numericValue` attribute . . . 15
- 3.11 **SpeciesTypeInstance** . . . 16
  - 3.11.1 The `id` and `name` attributes . . . 16
  - 3.11.2 The `speciesType` attribute . . . 16
  - 3.11.3 The `compartmentReference` attribute . . . 16
- 3.12 **SpeciesTypeComponentIndex** . . . 17
  - 3.12.1 The `id` and `name` attributes . . . 17
  - 3.12.2 The `component` attribute . . . 17
  - 3.12.3 The `identifyingParent` attribute . . . 18
  - 3.12.4 Reference a `component` in a `speciesType` or a `species` . . . 18
- 3.13 **InSpeciesTypeBond** . . . 19
  - 3.13.1 The `id` and `name` attributes . . . 19
  - 3.13.2 The `bindingSite1` and `bindingSite2` attributes . . . 20
- 3.14 Uniqueness of **SpeciesType** definitions . . . 20
- 3.15 Extended **Species** . . . 22
  - 3.15.1 The `speciesType` attribute . . . 22
  - 3.15.2 **ListOfOutwardBindingSites** . . . 22
  - 3.15.3 **ListOfSpeciesFeatures** . . . 23
- 3.16 **OutwardBindingSite** . . . 23

3.16.1 The id and name attributes . . . 23
3.16.2 The bindingStatus attribute . . . 23
3.16.3 The component attribute . . . 23
3.16.4 Example . . . 24
3.17 **SubListOfSpeciesFeatures** . . . 25
3.17.1 The id and name attributes . . . 25
3.17.2 The relation attribute . . . 25
3.17.3 The component attribute . . . 25
3.18 **SpeciesFeature** . . . 25
3.18.1 The id and name attributes . . . 26
3.18.2 The speciesFeatureType attribute . . . 26
3.18.3 The occur attribute . . . 26
3.18.4 The component attribute . . . 27
3.18.5 **ListOfSpeciesFeatureValues** . . . 27
3.18.6 **SpeciesFeatureValue** . . . 27
3.18.7 Example . . . 27
3.19 *Fully defined* species and mapping to *pattern* species . . . 29
3.20 **Reaction** . . . 29
3.21 **IntraSpeciesReaction** . . . 30
3.22 Extended **SimpleSpeciesReference** . . . 30
3.23 Extended **SpeciesReference** . . . 32
3.23.1 **ListOfSpeciesTypeComponentMapsInProduct** . . . 32
3.24 **SpeciesTypeComponentMapInProduct** . . . 32
3.24.1 The id and name attributes . . . 32
3.24.2 The reactant attribute . . . 33
3.24.3 The reactantComponent attribute . . . 33
3.24.4 The productComponent attribute . . . 33
3.25 OutwardBindingSites and speciesFeatures in *don't care* state in reaction products . . . 33
3.26 Extended ci elements in **Math** objects . . . 33
3.26.1 The speciesReference attribute . . . 33
3.26.2 The representationType attribute . . . 34
3.27 Namespace scoping rules for identifiers . . . 35
**4 Examples** **36**
4.1 Example: **Compartment**, **SpeciesType**, and **Species** . . . 36
4.2 *Simmune* example: the Ecad model . . . 37
4.3 A *BioNetGen* example from its user manual . . . 45
4.4 Example from *Kappa*'s documentation . . . 57
**A Validation of SBML documents using Multi constructs** **60**
**Acknowledgments** **72**
**References** **73**

## 1 Introduction

This Multistate, Multicomponent and Multicompartment Species (Multi) package provides an extension of SBML Level 3 [Hucka et al. (2016)] that supports encoding `models` with molecular complexes that have multiple components and can exist in multiple states and in multiple `compartments`. One of its goals is to provide a platform for sharing `models` based on the specifications of molecular transformations/interactions and the rules governing such reactions [Angermann et al. (2012); Feret et al. (2009); Hlavacek et al. (2006); Zhang et al. (2013)]. This specification covers the goals and features described in the previous Multi proposal [Le Novère and Oellrich (2010)] for extending SBML to carry the information for *multistate multicomponent* `species` with revised data structure. In addition, this specification includes the feature for *multicompartment* `species` as described in the releases of the Multi proposal [Zhang and Meier-Schellersheim (2013a), Zhang et al. (2012)].

### 1.1 Proposal and specifications

The proposal corresponding to this package specification is available at:

http://sbml.org/Community/Wiki/SBML_Level_3_Proposals/Multistate_and_Multicomponent_Species_Proposal

The specifications (v1.0.1 to current) are located at:

https://sourceforge.net/p/sbml/code/HEAD/tree/trunk/specifications/sbml-level-3/version-1/multi/spec/

### 1.2 Package dependencies

The Multi package has no dependencies on other SBML Level 3 packages.

### 1.3 Document conventions

UML 1.0 notation is used in this document to define the constructs provided by this package. Colors in the diagrams carry the following additional information for the benefit of those viewing the document on media that can display color:

- *Black* Items colored black are components taken unchanged from their definitions in the SBML Level 3 Core specification document.
- *Green* Items colored green are components that exist in SBML Level 3 Core, but are extended by this package. Class boxes are also drawn with with dashed lines to further distinguish them.
- Blue Items colored blue are new components introduced in this package specification. They have no equivalent in the SBML Level 3 Core specification.
- Red

For other matters involving the use of UML, XML and typographical conventions, this document follows the conventions used in the SBML Level 3 Core specification document [Hucka et al. (2016)].

For simplicity, "`...`" in all example code refers to some unspecified code content, that is not important for the purpose of illustrating the issue at hand.

We use red color in text to indicate changes between this version of the specification, namely SBML Multi Package Version 1 *Release 2*, and the most recent previous release of the specification (which, for the present case, is SBML Multi Package Version 1 *Release 1*).

## 2 Background and context

Rule-based, domain-detailed modeling has been extremely valuable in systems biology related studies [Manes et al. (2015) and Miskov-Zivanov1 et al. (2013)]. Rule-based, domain-detailed modeling approaches (*BioNetGen* [Faeder et al. (2009)], *Kappa* [Danos and Laneve (2004)], and *Simmune* [Angermann et al. (2012); Meier-Schellersheim et al. (2006)]) define rules for transformations of molecular domains and interactions between pairs of molecule domains, specifying how the transformations/interactions depend on particular states of the molecules (pattern) and their locations in specific compartments. In order to generate networks of biochemical reactions these rules are applied to the molecular components of the systems to be modeled, either at the beginning of the modeling (simulation) process or "on the fly" (as molecule complexes emerge from the interaction rules). Expressing such rule-based, domain-detailed reaction networks using the concepts of **Species** and **Compartment** in SBML (L3 core and L2) can be difficult for rules and molecule sets that lead to large numbers of resulting molecular complexes. It would therefore be desirable to have an SBML standard for encoding rule-based, domain-detailed `model`s using their "native" concepts for describing reactions instead of having to apply the rules and unfold the networks prior to encoding in an SBML format.

We proposed a revised proposal of the Multi package: "Multistate, Multicomponent and Multicompartment Species Package for SBML Level 3" (abbreviated as Multi) [Zhang et al. (2012) and Zhang and Meier-Schellersheim (2013a)] which takes the scopes and some data structures developed in the previous Multi proposal [Le Novère and Oellrich (2010)] and addresses main issues arising from a rule-based, domain-detailed modeling point of view with the data structures consistent with that used in the available rule-based, domain-detailed modeling tools.

☞ *Note:*
*This specification was developed with the main goal of taking into account molecular transformations and bi-molecular interactions mediated through specific binding domains (or sites). Models without such detailed description of the molecular interactions can be encoded as well if the other features in this specification such as* ***SpeciesFeatureType***, ***SpeciesFeature***, *and extended* ***Compartment*** *satisfy the model requirements.*

### 2.1 Past work on this problem or similar topics

- Nicolas Le Novère and Anika Oellrich proposed the previous version of the Multi proposal [Le Novère and Oellrich (2010)]. However, it was realized that a more detailed treatment of molecular binding sites and their state-dependent interactions would be desirable.

- In August 2012, Fengkai Zhang from the *Simmune* group presented " Draft for discussion SBML Proposals for Revised Multi, Simple Spatial and Multi-Spatial Extensions" at COMBINE 2012 [Zhang et al. (2012)]. The three proposals cover the goals and scope of the previous Multi proposal (2010), revise it and add some new features that improve usage of the proposal for rule-based approaches.

- Based on the discussions and suggestions received during COMBINE 2012 as well as on feedback from the SBML discussion forum, the new Multi proposal [Rev 221, Zhang and Meier-Schellersheim (2012)] was released to the SBML-Multi community, which integrates and covers most of the features in the three previous proposals of August 2012.

- In May 2013, a new revision (rev 280) of the Multi proposal [Zhang and Meier-Schellersheim (2013a)] was released before the meeting of HARMONY 2013. The extended **Compartment** class and its related classes have been reorganized. All optional boolean attributes have been removed/replaced. A new optional Multi attribute, "`whichValue`", was added to the `ci` elements in **KineticLaw** to identify the sources of `species`. (Lucian Smith gave many comments/suggestions about this proposal and William Hlavacek gave thoughtful feedback about the *BioNetGen* example in this proposal). This revision (rev 280) was presented at HARMONY 2013 [Zhang and Meier-Schellersheim (2013b)] with new features to configure multiple occurrences of **SpeciesFeatureType**. Several new or revised features were discussed during and after HARMONY 2013, including multiple occurrences of **SpeciesFeatureType**, multiple copies of **SpeciesTypeInstance**, the `numericValue` attribute for

**PossibleSpeciesFeatureValue** and concentration summation of pattern `species`. These features are covered or updated in the specifications from v1.0.1.

### 2.2 Revision history

The versioning convention used in this document:
`x.y.z (status)`

`x`: version of SBML Level 3 core.
`y`: version of the Multi package.
`z`: release of the Multi package at its version `y`.
`status`: "`draft`", "`release candidate`", or "`release`". Absence of status means "`release`".

For example, the current version is "`1.1.2`"
`x` = "`1`"
`y` = "`1`"
`z` = "`2`"

The followings are the revision history of the Multi package:

#### *2.2.1 Release(s)*

- **Version: 1.1.2**, this version
  - Fix issues raised by the community after "Version 1 release 1".
  - Revise parts of the text.
  - Add a paragraph to clarify "XML Namespace use"(see Section 3.1 on page 9).
  - Validate the complete models and add "demonstration only" notes to those "incomplete" models in the example section (see Section 4 on page 36).
- **Version: 1.1.1, March 2017**

#### *2.2.2 Release Candidates*

- **Version: 1.1.rc5 (release candidate), March 2017**
  - Add two validation rules **multi-21213** and **multi-21214** to check the `speciesType` attribute of a `species` with `listOfOutwardBindingSites` and/or `listOfSpeciesFeatures`(See Section 3.14 on page 22.)
  - Add a constraint to the `relation` attribute of a `subListOfSpeciesFeatures` having a `speciesFeature` child referencing a `speciesFeatureType` with "`occur > 1`". (See Section 3.17.2 on page 25 and Section A on page 68.)
  - Enforce the **SubListOfSpeciesFeatures** class to have at least two `speciesFeature`s and set `relation` as a required attribute. (See Section 3.17 on page 25.)
- **Version: 1.1.rc4 (release candidate), March 2017**
  More updates on validation rule numbers, line breaks, and the example about **SubListOfSpeciesFeatures**.
- **Version: 1.1.rc3 (release candidate), Febrary 2017**
  Modify the numbers of several rules to be consistent with the general SBML validation rule conventions.
- **Version: 1.1.rc2 (release candidate), January 2017**
  Add a new validation rule **multi-22006** to prevent circular referencing among the extended **Compartment** objects.

Revise the specification text with minor changes towards a version of the official release candidate.

- **Version: 1.1.rc1 (release candidate), November 2016**
  Revise the specification text with minor changes towards a version of the official release candidate.

#### 2.2.3 Drafts

- **Version: 1.0.7 (draft), August 2016**
  Remove the **SpeciesFeatureChange** and **ListOfSpeciesFeatureChanges** classes under **SpeciesTypeComponentMapInProduct**. The relations expressed in **SpeciesFeatureChange** can be inferred from the `speciesTypeComponentMapInProduct` and the `species` of the mapped `reactant` and `product`.

Add a new validation rule 21306, "an `outwardBindingSite` cannot be a binding site in a bond of the species" (see Section 3.16.3 on page 23 and Section A on page 69)

- **Version: 1.0.6 (draft), March 2016**
  Remove recursively referencing relationship in the **ListOfSpeciesFeatures** class and add a **SubListOfSpeciesFeatures** class. See the details in **Species**.

Version 1.0.6.1 with minor document update is released in April 2016.

- **Version 1.0.5 (draft), November 2015**
  This version has been developed from the previous release v1.0.4 with the following modifications based on the discussion during and after COMBINE 2015 [Zhang (2015)]:

- Drop the `occur` attribute in the class of **SpeciesTypeInstance**.
  - Drop the `occur` attribute in the class of **SpeciesTypeComponentIndex**.
  - Drop the class of **DenotedSpeciesTypeComponentIndex**.
  - Revise the scope of **PossibleSpeciesFeatureValue** ids to be global.

Version 1.0.5.1 with minor document update is released in Dec 2015.

- **Version 1.0.4 (draft), June 2015**
  This version has been developed from the previous release v1.0.3 with minor document update and complete validation rules.

- **Version 1.0.3 (draft), April 2015**
  This version has been developed from the previous release v1.0.2 mainly based on the discussion in COMBINE 2014 with focus on how to facilitate tools to export and import `model`s encoded in the Multi format [Zhang and Meier-Schellersheim (2014)]

- **Version 1.0.2 (draft), November 2014**
  This version has been developed from the previous release v1.0.1 with the following modifications:
  - A new **BindingSiteSpeciesType** sub-class inheriting the **SpeciesType** class for `binding sites`. Accordingly, the `isBindingSite` attribute has been dropped from **SpeciesType**.
  - Restriction on `binding sites` which have to be atomic.
  - Restriction on **SpeciesType** that a `speciesType` cannot have a `listOfSpeciesFeatureTypes` if it has a `listOfInSpeciesTypeBonds`.
  - A new **IntraSpeciesReaction** sub-class inheriting the **Reaction** class for the reactions happening within a **Species** object. Accordingly, the `isIntraSpeciesReaction` attribute has been dropped from **Reaction**.
  - Validation rules.

- **Version 1.0.1 (draft), September 2013**
  This was released and presented in COMBINE 2013 [Zhang and Meier-Schellersheim (2013c)], mainly addressing the scenario of multiple occurrences of identical components and/or identical features.

#### 2.2.4 Revision history before draft version 1.0.1

See the past work (Section 2.1 on page 6).

## 3 Package syntax and semantics

This section contains a definition of the syntax and semantics of the Multi package for SBML Level 3 Core.

### 3.1 Namespace URI and other declarations necessary for using this package

The following is the namespace URI for this version of the Multi package for SBML Level 3 Core:

"`http://www.sbml.org/sbml/level3/version1/multi/version1`"

In addition, SBML documents using a given package must indicate whether the package can be used to change the mathematical interpretation of a model. This is done using the attribute `required` on the `<sbml>` element in the SBML document. For the Multi package, the value of this attribute must be "`true`".

The following fragment illustrates the beginning of a typical SBML model using SBML Level 3 Core and this version of the Multi package:

```
<?xml version="1.0" encoding="UTF-8"?>
<sbml xmlns="http://www.sbml.org/sbml/level3/version1/core" level="3" version="1"
   xmlns:multi="http://www.sbml.org/sbml/level3/version1/multi/version1" multi:required="true">
```

***XML Namespace use***

For element names, XML has clear rules about how to declare and use namespaces. In typical SBML documents, the Multi package namespace will be defined as above, and the Multi package elements will therefore need to be prefixed with "`multi:`".

In contrast to element names, XML *attribute* names are completely defined by the element in which they appear, and never have a 'default' namespace defined: the element itself declares whether any attributes should be defined with a namespace prefix or not.

Any attribute that appears in a UML diagram in this specification may *either* be defined with no namespace prefix, *or* be defined with the *multi* namespace as a prefix. For attributes added to SBML core elements (**Compartment**, **Species**, **SimpleSpeciesReference**, and within **MathML**), the *multi* namespace prefix is required.

If the Multi package is used in an SBML Level 3 Version 2 document, *any* element defined here may have either an `id` or a `name` with no prefix, as `id` and `name` were added to ***SBase*** in that level/version.

### 3.2 Primitive data types

The Multi package uses a number of the primitive data types described in section 3.1 of the SBML Level 3 Core Release 2[Hucka et al. (2016)] specification such as `SId`, `SIdRef`, `string`, `boolean`, `int` and `positiveInteger`, and adds three additional primitive types described below.

#### 3.2.1 *Type* `BindingStatus`

The `BindingStatus` primitive data type is used in the definition of the **OutwardBindingSite** class. `BindingStatus` is derived from type `string` and its values are restricted to be one of the following possibilities: "`bound`", "`unbound`", and "`either`". Attributes of type `BindingStatus` cannot take on any other values. The meaning of these three values is discussed in the context of the **OutwardBindingSite** class in Section 3.16 on page 23.

#### 3.2.2 *Type* `Relation`

The `Relation` primitive data type is used in the definition of the **SubListOfSpeciesFeatures** class. `Relation` is derived from type `string` and its values are restricted to be one of the following possibilities: "`and`", "`or`", and "`not`". Attributes of type `Relation` cannot take on any other values. The meaning of these three values is discussed in the context of the **SubListOfSpeciesFeatures** class in Section 3.17 on page 25.

#### 3.2.3 *Type* `RepresentationType`

The `RepresentationType` primitive data type is used in the extension of the `ci` element. `RepresentationType` is derived from type `string` and its values are restricted to be one of the following possibilities: "`sum`" or "`numericValue`". If present, attributes of type `RepresentationType` cannot take on any other values. The meaning of these values is discussed in the context of the `ci` element in Section 3.26 on page 33.

### 3.3 The new and extended classes in the Multi Package

The Multi package extends the following object classes:
**Model**, **Compartment**, **Species**, **Reaction**, **SimpleSpeciesReference**, and **SpeciesReference**.

The Multi package defines the following object classes:
**CompartmentReference**, **SpeciesType**, **SpeciesFeatureType**, **PossibleSpeciesFeatureValue**, **SpeciesTypeInstance**, **InSpeciesTypeBond**, **SpeciesTypeComponentIndex**, **SubListOfSpeciesFeatures**, **OutwardBindingSite**, **SpeciesFeature**, **SpeciesFeatureValue**, **SpeciesTypeComponentMapInProduct**
and "**ListOf-**" classes:
**ListOfSpeciesTypes**, **ListOfCompartmentReferences**, **ListOfSpeciesTypeInstances**, **ListOfSpeciesFeatureTypes**, **ListOfInSpeciesTypeBonds**, **ListOfSpeciesTypeComponentIndexes**, **ListOfPossibleSpeciesFeatureValues**, **ListOfOutwardBindingSites**, **ListOfSpeciesFeatures**, **ListOfSpeciesFeatureValues**, **ListOfSpeciesTypeComponentMapsInProduct**.

All the classes in the Multi package are directly or indirectly derived from ***SBase***, and ***SBase*** provides the ability to attach `SBO` terms as well as `MIRIAM` annotations. The semantics of a given class in the Multi package can be made more precise by referencing to external controlled vocabularies and ontologies.

Like the classes in SBML Level 3 Core, most new Multi classes have the attribute `id` (typically mandatory but not all, and of type `SId`), which serves as an identifier to provide a way to identify the class object. The identifier of a class object reference may or may not carry mathematical interpretation or be used in mathematical formulas, depending on its class and the class object referencing it. The scope of `id`s is described in the section of "Namespace scoping rules for identifiers"(Section 3.27 on page 35).

### 3.4 Extended Model

The Multi package extends the **Model** class of SBML Level 3 Core and adds an optional **ListOfSpeciesTypes** child to **Model**. Figure 1 provides the UML diagram for the extended **Model** class.

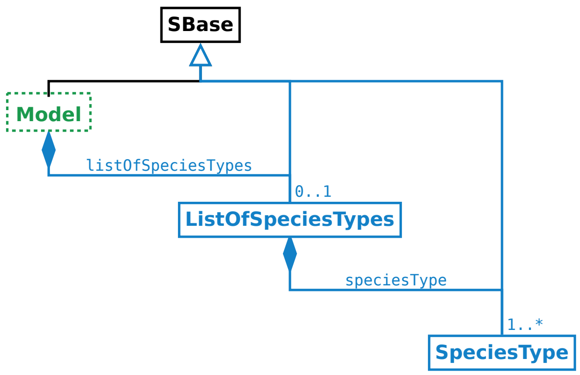

***Figure 1:*** *The extension of the **Model** class.*

#### 3.4.1 ListOfSpeciesTypes

**ListOfSpeciesTypes** is defined in Figure 1 on the preceding page. If present, a **ListOfSpeciesTypes** object must contain at least one **SpeciesType** object. Since **ListOfSpeciesTypes** is derived from **SBase**, it inherits the `sboTerm` and `metaid` attributes, as well as the optional child **Notes** and **Annotation** objects.

### 3.5 Extended Compartment

A **Compartment** object in SBML Level 3 Core represents a bounded space in which *species* are located. In the Multi package, **Compartment** is extended. A Multi `compartment` can be a `type` that multiple referencing `compartments` can map to. A Multi `compartment` can also be a composite `compartment` or a container that includes other `compartments`.

The extension of **Compartment** is defined in Figure 2. The extended **Compartment** class has a new required attribute `isType`, a new optional attribute `compartmentType` and an optional **ListOfCompartmentReferences** child. The example at Section 4.1 on page 36 illustrates the use of the extended **Compartment** class.

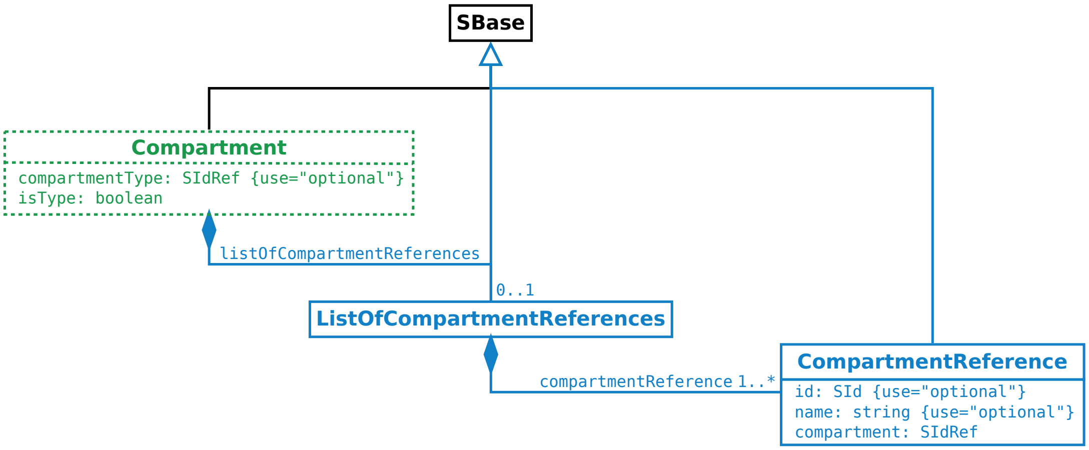

***Figure 2:*** *The definitions of **Compartment**, **ListOfCompartmentReferences** and **CompartmentReference***

#### 3.5.1 The `isType` attribute

The required attribute `isType`, of type `boolean`, on the **Compartment** class serves to provide a way to indicate whether the **Compartment** object is a compartment type.

A **Compartment** object is a type of compartment if the value of its `isType` attribute is "`true`". A type of compartment is a template (in the sense of prototype) for all **Compartment** objects referencing it (via `compartmentType` attributes).

☞ *Note:*
*A **Species** object directly referencing a compartment type is not a* fully defined `species` *(see Section 3.19 on page 29).*

If the value of the `isType` attribute is "`false`", the **Compartment** object is a "`not-a-type`" *compartment* , and it is similar to a SBML core `compartment` except it can reference a compartment type and can have a **ListOfCompartmentReferences** child.

#### 3.5.2 The `compartmentType` attribute

The optional attribute `compartmentType`, of type `SIdRef`, is used for a "`not-a-type`" `compartment` to reference a compartment type. A `compartment` with the "`true`" value of its `isType` attribute cannot have the `compartmentType` attribute defined.

#### 3.5.3 ListOfCompartmentReferences

**ListOfCompartmentReferences** is defined in Figure 2 on the preceding page, and is extended from the **ListOf** class. A `listOfCompartmentReferences` must have one or more **CompartmentReference** children. Since **ListOfCompartmentReferences** is derived from **SBase** through **ListOf**, it inherits the `sboTerm` and `metaid` attributes, as well as the optional child **Notes** and **Annotation** objects.

### 3.6 CompartmentReference

**CompartmentReference** is defined in Figure 2 on the previous page. It has two optional attributes `id` and `name`, and a required attribute `compartment`. Since **CompartmentReference** is derived from **SBase**, it inherits the `sboTerm` and `metaid` attributes, as well as the optional child **Notes** and **Annotation** objects.

#### 3.6.1 The `id` and `name` attributes

The optional `id` attribute, of type `SId`, serves to provide a way to identify a `compartmentReference`. **CompartmentReference** also has an optional `name` attribute of type `string`.

☞ *Note:*
*If some or all* `compartmentReference`*s within a* ***ListOfCompartmentReferences*** *object reference the same* `compartment`*, those* `compartmentReference`*s are required to have their* `id` *attributes defined to distinguish them.*

#### 3.6.2 The `compartment` attribute

The required `compartment` attribute, of type `SIdRef`, serves to provide a way to reference a **Compartment** object.

☞ *Note:*
*A* `compartmentReference` *cannot reference a* `compartment` *that directly or indirectly contains the* `compartmentReference`*. In other words, circular references are not allowed when constructing* `compartment`*s and* `compartmentReference`*s.*

### 3.7 The relationship of Compartment, CompartmentReference and ListOfCompartmentReferences

In a **ListOfCompartmentReferences** object, every child `compartmentReference`s must exclusively reference, directly or indirectly, "`not-a-type`" `compartment` which can be of the same compartment type. See the extended **Compartment** objects in the example in Section 4.1 on page 36.

All `compartment`s referenced by a `listOfCompartmentReferences` must have the values of their `isType` attributes the same as that in the parent `compartment` of the `listOfCompartmentReferences`. For example, a compartment "`A`" with `isType`="`true`" has a `listOfCompartmentReferences` referencing two compartments "`A1`" and "`A2`". Then, "`A1`" and "`A2`" must have `isType`="`true`".

### 3.8 SpeciesType

**SpeciesType** is defined in Figure 3 on the next page and serves to provide backbone structures for `species`. **SpeciesType** has one required attribute, `id`, two optional attributes, `name` and `compartment` and four optional **ListOf___** objects, including **ListOfSpeciesFeatureTypes**, **ListOfSpeciesTypeInstances**, **ListOfInSpeciesTypeBonds** and **ListOfSpeciesTypeComponentIndexes**. Since **SpeciesType** is derived from **SBase**, it inherits the `sboTerm` and `metaid` attributes, as well as the optional child **Notes** and **Annotation** objects.

The **ListOfSpeciesTypeInstances** element provides a way to define multicomponents which are instances of other **SpeciesType** objects.

The **ListOfSpeciesFeatureTypes** element and its **SpeciesFeatureType** child set up a framework for the referencing `species` or the instances of `speciesType`s to be able to have multistates. The **ListOfSpeciesTypeComponent-**

**Indexes** element provides a way to reference a `component` in a `speciesType`.

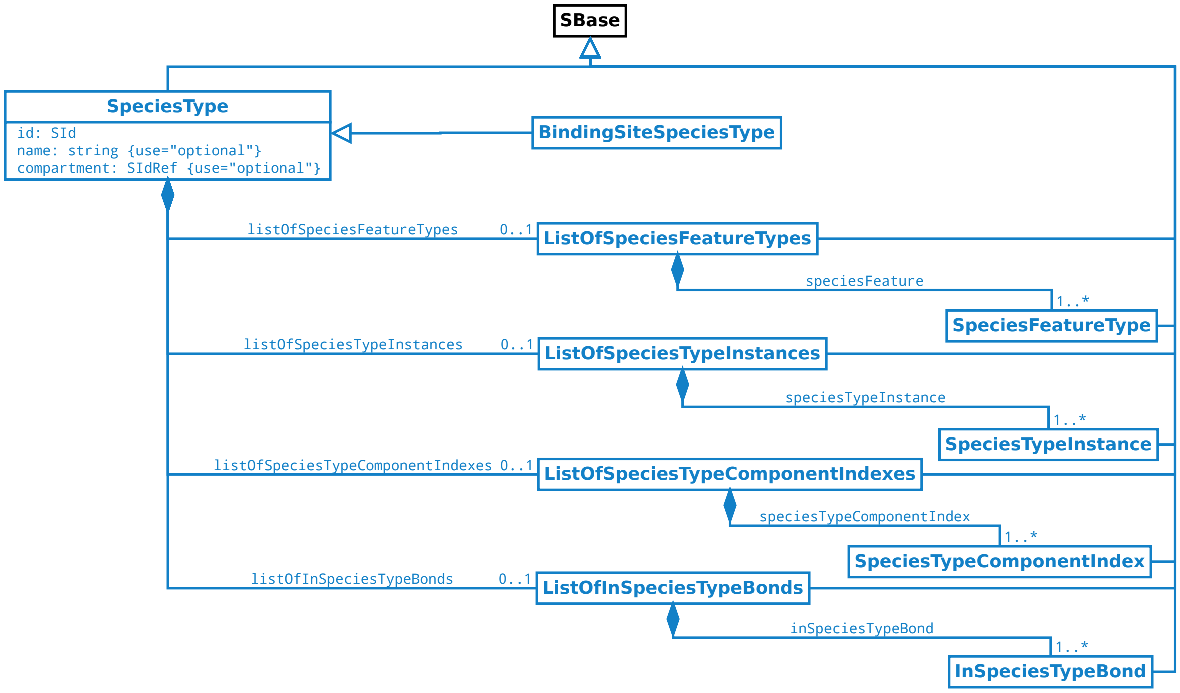

***Figure 3:*** *The definition of the* ***SpeciesType*** *class.*

#### 3.8.1 The `id` and `name` attributes

The required `id` attribute, of type `SId`, serves to provide a way to identify a `speciesType`. **SpeciesType** also has an optional `name` attribute of type `string`.

#### 3.8.2 The `compartment` attribute

**SpeciesType** has an optional attribute `compartment`, of type `SIdRef`, to be used to identify the `compartment` where the `speciesType` is located. The attribute value must be the identifier of an existing `compartment` in the `model`. If present, it must be consistent with the `compartment` attributes of the referencing `species` (see Section 3.14 on page 22) and the `compartmentReference` attributes of its instances (see Section 3.11.3 on page 16). The example in Section 4.1 on page 36 illustrates how to keep the consistency of this attribute.

#### 3.8.3 ListOfSpeciesFeatureTypes

**ListOfSpeciesFeatureTypes** is defined in Figure 3, and is extended from the **ListOf** class. If present, a **ListOfSpeciesFeatureTypes** object must have one or more **SpeciesFeatureType** children. Since **ListOfSpeciesFeatureTypes** is derived from **SBase** through **ListOf**, it inherits the `sboTerm` and `metaid` attributes, as well as the optional child **Notes** and **Annotation** objects.

#### 3.8.4 ListOfSpeciesTypeInstances

**ListOfSpeciesTypeInstances** is defined in Figure 3, and is extended from the **ListOf** class. If present, a **ListOfSpeciesTypeInstances** object must have one or more **SpeciesTypeInstance** children. Since **ListOfSpeciesTypeInstances** is derived from **SBase** through **ListOf**, it inherits the `sboTerm` and `metaid` attributes, as well as the optional child

**Notes** and **Annotation** objects.

#### 3.8.5 ListOfInSpeciesTypeBonds

**ListOfInSpeciesTypeBonds** class is defined in Figure 3 on the previous page, and is extended from the **ListOf** class. If present, a **ListOfInSpeciesTypeBonds** object must have one or more **InSpeciesTypeBond** children. Since **ListOfInSpeciesTypeBonds** is derived from **SBase** through **ListOf**, it inherits the sboTerm and metaid attributes, as well as the optional child **Notes** and **Annotation** objects.

#### 3.8.6 ListOfSpeciesTypeComponentIndexes

**ListOfSpeciesTypeComponentIndexes** is defined in Figure 3 on the preceding page, and is extended from the **ListOf** class. If present, a **ListOfSpeciesTypeComponentIndexes** object must have one or more **SpeciesTypeComponent-Index** children. Since **ListOfSpeciesTypeComponentIndexes** is derived from **SBase** through **ListOf**, it inherits the sboTerm and metaid attributes, as well as the optional child **Notes** and **Annotation** objects.

#### 3.8.7 BindingSiteSpeciesType

**BindingSiteSpeciesType** inherits the **SpeciesType** class and is defined in Figure 3 on the previous page. A **Bind-ingSiteSpeciesType** object is a binding site, and its instance can further define the bindingStatus attribute and can participate a binding internally and explicitly in an **InSpeciesTypeBond** object, or externally and implicitly defined by an **OutwardBindingSite** object. A binding site must be an atomic component which means that a **BindingSiteSpeciesType** object cannot contain a **ListOfSpeciesTypeInstances** element.

☞ *Note:*
*In the Multi package, a binding site can only participate in one binding at a time. That means a binding site cannot bind two partners at the same time. Binding is one-to-one at all times.*

### 3.9 SpeciesFeatureType

**SpeciesFeatureType** is defined in Figure 4 on the following page, and serves to provide frameworks or templates to define the referencing **SpeciesFeature** objects. **SpeciesFeatureType** has two required attributes id and occur, an optional attribute name, and a required child **ListOfPossibleSpeciesFeatureValues** element. The multiple **Possible-SpeciesFeatureValue** elements in the **ListOfPossibleSpeciesFeatureValues** object permit constructing multistate species via its speciesFeatures under the **ListOfSpeciesFeatures** or **SubListOfSpeciesFeatures** object. Since **SpeciesFeatureType** is derived from **SBase**, it inherits the sboTerm and metaid attributes, as well as the optional child **Notes** and **Annotation** objects.

#### 3.9.1 The id and name attributes

The required id attribute, of type SId, serves to provide a way to identify a speciesFeatureType. Its value must be unique within its direct parent speciesType. When a speciesFeatureType is referenced by a speciesFeature, a **SpeciesTypeComponentIndex** object indexing the containing component can be used to avoid ambiguity.

**SpeciesFeatureType** also has an optional name attribute of type string.

#### 3.9.2 The occur attribute

**SpeciesFeatureType** has a required attribute occur, of type positiveInteger, used to indicate the number of instances of the speciesFeatureType. This attribute can be used to infer the number of the instances in *don't care* state with the use of the occur attribute in a referencing speciesFeature (also see Section 3.18.3 on page 26).

#### 3.9.3 ListOfPossibleSpeciesFeatureValues

**ListOfPossibleSpeciesFeatureValues** is defined in Figure 4 on the following page, and is extended from the **ListOf** class. A listOfPossible- SpeciesFeatureValues must have one or more **PossibleSpeciesFeatureValue** chil-

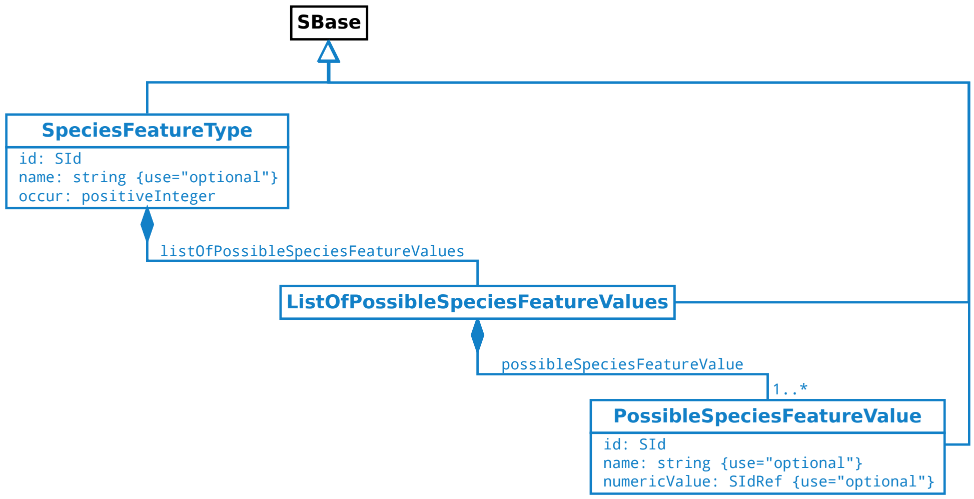

***Figure 4:*** *The definitions of **SpeciesFeatureType**, **ListOfPossibleSpeciesFeatureValues** and **PossibleSpeciesFeatureValue** classes.*

dren. Since **ListOfPossibleSpeciesFeatureValues** is derived from **SBase** through **ListOf**, it inherits the `sboTerm` and `metaid` attributes, as well as the optional child **Notes** and **Annotation** objects.

### 3.10 PossibleSpeciesFeatureValue

**PossibleSpeciesFeatureValue** is defined in Figure 4, and is used to define the possible values a `speciesFeature` can take. It has a required attribute `id` and two optional attributes `name` and `numericValue`. Since **PossibleSpeciesFeatureValue** is derived from **SBase**, it inherits the `sboTerm` and `metaid` attributes, as well as the optional child **Notes** and **Annotation** objects.

#### *3.10.1 The `id` and `name` attributes*

The required `id` attribute, of type `SId`, serves to provide a way to identify a `possibleSpeciesFeatureValue`.

If the `id` of a `possibleSpeciesFeatureValue` is the content of a `ci` element in a MathML expression, it can either represent the `numericValue` ( when the `ci` has `representationType`="`numericValue`") or the count of the feature instances (default) which have this value.

**PossibleSpeciesFeatureValue** also has an optional `name` attribute of type `string`.

#### *3.10.2 The `numericValue` attribute*

**PossibleSpeciesFeatureValue** has an optional attribute `numericValue` to be used to provide a reference to a numeric value that the **PossibleSpeciesFeatureValue** object can have. This attribute has type of `SIdRef`, and the value must be the identifier of a **Parameter** object in the `model`. The numeric value along with the unit can be defined in the **Parameter** object.

The modeler can either use the identifier of the `parameter`, or the identifier of the `possibleSpeciesFeatureValue` (with `ci`'s `representationType` and `speciesReference` attribute) as the content of a `ci` element to represent its value in `MathML` expressions in SBML.

### 3.11 SpeciesTypeInstance

**SpeciesTypeInstance** serves to provide a way to construct `speciesType`s and `species` with multiple components. A `speciesType` can contain a list of instances of other `speciesType`s which can also have their own `speciesTypeInstance`s, so a `speciesType` has a tree structure. A `speciesType` cannot contain an instance of any other `speciesType` that already contains an instance of it. In other words, circular references are not allowed when constructing `speciesType`s. For example, if a speciesType "`A`" contains an instance of another speciesType "`B`", "`B`" must not contain an instance of "`A`" anywhere in the complete structure of "`B`".

**SpeciesTypeInstance** is defined in Figure 5. It has two required attributes, `id`, and `speciesType`, and two optional attributes `name` and `compartmentReference`. Since **SpeciesTypeInstance** is derived from **SBase**, it inherits the `sboTerm` and `metaid` attributes, as well as the optional child **Notes** and **Annotation** objects.

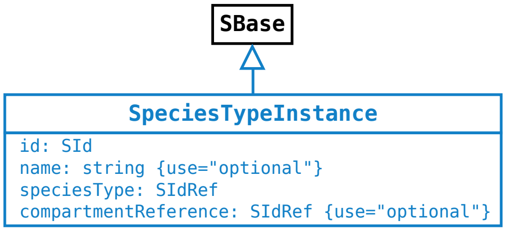

***Figure 5:*** *The definition of the* ***SpeciesTypeInstance*** *class*

#### 3.11.1 *The* `id` *and* `name` *attributes*

The required attribute `id` , of type `SId`, serves to provide a way to identify a `speciesTypeInstance`. Its value must be unique within its direct parent `speciesType`.

**SpeciesTypeInstance** also has an optional `name` attribute of type `string`.

#### 3.11.2 *The* `speciesType` *attribute*

The required attribute `speciesType`, of type `SIdRef`, is used to reference a `speciesType`.

#### 3.11.3 *The* `compartmentReference` *attribute*

The optional attribute `compartmentReference`, of type `SIdRef`, can be used to indicate which sub-compartment in a composite `compartment` the `speciesTypeInstance` is located in.

For example, a compartment "`cA`" has two sub-compartments "`cB1`" (referenced by compartmentReference "`crB1`") and "`cB2`" (referenced by compartmentReference "`crB2`") of the same compartment type "`cB`". A speciesType "`stA`" has two speciesTypeInstances "`stiB1`" and "`stiB2`" of the same speciesType "`stB`". The speciesType "`stA`" references the compartment "`cA`" and the speciesType "`stB`" references the compartment "`cB`". The speciesTypeInstance "`stiB1`" is located in "`cB1`" via the compartmentReference "`crB1`" and the speciesTypeInstance "`stiB2`" is located in "`cB2`" via the compartmentReference "`crB2`". The SBML code can be as follows:

```
<listOfCompartments>
   <compartment id="cB" multi:isType="true" ... />
   <compartment id="cB1" multi:isType="false" multi:compartmentType="cB" ... />
   <compartment id="cB2" multi:isType="false" multi:compartmentType="cB" ... />
   <compartment id="cA" multi:isType="false" ... >
      <multi:listOfCompartmentReferences>
         <multi:compartmentReference multi:id="crB1" multi:compartment="cB1" />
         <multi:compartmentReference multi:id="crB2" multi:compartment="cB2" />
      </multi:listOfCompartmentReferences>
   </compartment>
```

```
</listOfCompartments>
<multi:listOfSpeciesTypes>
   <multi:speciesType multi:id="stB" multi:compartment="cB" ... />
   <multi:speciesType multi:id="stA" multi:compartment="cA" ... >
      <multi:listOfSpeciesTypeInstances>
         <multi:speciesTypeInstance multi:id="stiB1" multi:speciesType="stB"
            multi:compartmentReference="crB1" ... />
         <multi:speciesTypeInstance multi:id="stiB2" multi:speciesType="stB"
            multi:compartmentReference="crB2" ... />
      </multi:listOfSpeciesTypeInstances>
   </multi:speciesType>
</multi:listOfSpeciesTypes>
```

### 3.12 SpeciesTypeComponentIndex

**SpeciesTypeComponentIndex** provides a way to identify or index a `component` within a `speciesType`. A **SpeciesTypeComponentIndex** object can be referenced by other classes, such as **InSpeciesTypeBond**, **OutwardBindingSite**, **SpeciesFeature** or **SpeciesTypeComponentMapInProduct**, which needs to identify a component in a particular `speciesType`.

**SpeciesTypeComponentIndex** is defined in Figure 6. It has two required attributes, `id`, and `component`, and two optional attributes `name` and `identifyingParent`. Since **SpeciesTypeComponentIndex** is derived from **SBase**, it inherits the `sboTerm` and `metaid` attributes, as well as the optional child **Notes** and **Annotation** objects.

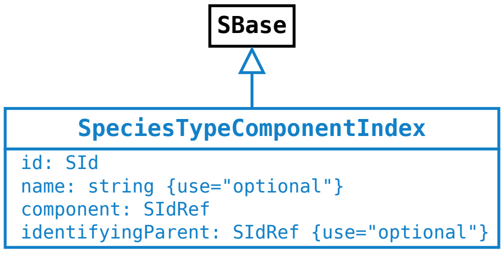

***Figure 6:*** *The definition of the* ***SpeciesTypeComponentIndex*** *class*

See Section 3.16.3 on page 23 about how to use **SpeciesTypeComponentIndex** in an `outwardBindingSite`.

*Note:*
*A* `speciesTypeComponentIndex` *should be unambiguous. For example, a* `speciesTypeComponentIndex` *should not reference to a* `speciesType` *which is referenced by two* `speciesTypeInstance`*s contained in the same* ***SpeciesType*** *object.*

#### *3.12.1 The* `id` *and* `name` *attributes*

The `id` attribute, of type `SId`, provides a way to identify a `speciesTypeComponentIndex`. The value must be unique within the direct parent `speciesType`. **SpeciesTypeComponentIndex** also has an optional `name` attribute of type `string`.

#### *3.12.2 The* `component` *attribute*

The `component` attribute, of type of `SIdRef`, references a `speciesTypeInstance` in the `speciesType`, or the `speciesType` itself. The value of this attribute can be the `id` of a `speciesTypeInstance` or a `speciesTypeComponentIndex` that is defined in the `speciesType` of a `speciesTypeInstance`.

#### 3.12.3 *The* `identifyingParent` *attribute*

The `component` attribute itself may not be sufficient to uniquely reference a component in a `speciesType`. The `identifyingParent` attribute provides assistance for the identification of a **`component`**. It references a parent of the component and the value can be the `id` of a **SpeciesTypeInstance** object, **SpeciesTypeComponentIndex** or **SpeciesType**.

This example illustrates the use of the `identifyingParent` attribute. There are three `speciesType`s "`stA`", "`stB`" and "`stC`". The `speciesType` "`stB`" contains two `speciesTypeInstance`s "`C1`" and "`C2`" of the same `speciesType` "`stC`". The `speciesType` "`stA`" contains two `speciesTypeInstance`s "`B1`" and "`B2`" of the same `speciesType` "`stB`". The `speciesType` "`A`" may be required to index every "`C1`" and "`C2`" by its **ListOfInSpeciesTypeBonds** child or referencing `species`. The following SBML code demonstrates how to do the indexing with assistance from the `identifyingParent` attribute.

```
<multi:listOfSpeciesTypes>
   <multi:speciesType multi:id="stC" ... />
   <multi:speciesType multi:id="stB" ... >
      <multi:listOfSpeciesTypeInstances>
         <multi:speciesTypeInstance multi:id="C1" multi:speciesType="stC" />
         <multi:speciesTypeInstance multi:id="C2" multi:speciesType="stC" />
      </multi:listOfSpeciesTypeInstances>
   </multi:speciesType>
   <multi:speciesType multi:id="stA" ... >
      <multi:listOfSpeciesTypeInstances>
         <multi:speciesTypeInstance multi:id="B1" multi:speciesType="stB" />
         <multi:speciesTypeInstance multi:id="B2" multi:speciesType="stB" />
      </multi:listOfSpeciesTypeInstances>
      <multi:listOfSpeciesTypeComponentIndexes>
         <multi:speciesTypeComponentIndex multi:id="B1C1"
            multi:component="C1" multi:identifyingParent="B1" />
         <multi:speciesTypeComponentIndex multi:id="B1C2"
            multi:component="C2" multi:identifyingParent="B1" />
         <multi:speciesTypeComponentIndex multi:id="B2C1"
            multi:component="C1" multi:identifyingParent="B2" />
         <multi:speciesTypeComponentIndex multi:id="B2C2"
            multi:component="C2" multi:identifyingParent="B2" />
      </multi:listOfSpeciesTypeComponentIndexes>
      ...
   </multi:speciesType>
   ...
</multi:listOfSpeciesTypes>
```

In the speciesType "`stA`", "`B1C1`" identifies the "`C1`" in "`B1`" and "`B2C1`" identifies the "`C1`" in "`B2`". Similarly, "`B1C2`" identifies the "`C2`" in "`B1`" and "`B2C2`" identifies "`C2`" in "`B2`".

#### 3.12.4 *Reference a* `component` *in a* `speciesType` *or a* `species`

In the Multi package, component(s) of a `speciesType` (or a `species` via its `speciesType` attribute) can be referenced by objects of multiple classes such as **OutwardBindingSite** and **SpeciesFeature**. A component of a `speciesType` can be a `speciesTypeInstance` or the `speciesType` itself. For example:

```
<multi:listOfSpeciesTypes>
   <multi:bindingSiteSpeciesType multi:id="stA" ... />
   <multi:speciesType multi:id="stB" ...>
      ...
   </multi:speciesType>
   <multi:speciesType multi:id="stABB" ...>
      <multi:listOfSpeciesTypeInstances>
         <multi:speciesTypeInstance multi:id="stiA" multi:speciesType="stA" .../>
         <multi:speciesTypeInstance multi:id="stiB1" multi:speciesType="stB" .../>
         <multi:speciesTypeInstance multi:id="stiB2" multi:speciesType="stB" .../>
```

```
      </multi:listOfSpeciesTypeInstances>
   </multi:speciesType>
</multi:listOfSpeciesTypes>
<listOfSpecies>
   <species id="spA" multi:speciesType="stA" ...>
      <multi:listOfOutwardBindingSites>
         <multi:outWardBindingSite multi:component="stA" multi:bindingStatus="bound" />
      </multi:listOfOutwardBindingSites>
   </species>
   <species id="spABB" multi:speciesType="stABB" ...>
      <multi:listOfOutwardBindingSites>
         <multi:outWardBindingSite multi:component="stiA" multi:bindingStatus="bound" />
      </multi:listOfOutwardBindingSites>
      ...
    </species>
</listOfSpecies>
```

In this example, the component of the outwardBindingSite in species“spABB” is a speciesTypeInstance (“spABB”), and the component of the outwardBindingSite in species“spA” is a speciesType (“stA”) which is directly referenced by the speciesType attribute of “spA”.

In many cases, to reference a component, the id of the referenced component will be sufficient and it is not necessary to create an index (speciesTypeComponentIndex). The example in Section 3.12.3 on the previous page illustrates two equivalent ways to reference a component, for example, the “B1” component in the “stA” speciesType. The creation of a speciesTypeComponentIndex cannot be avoided when a speciesType (indirectly) has two speciesTypeInstances that have the same id.

### 3.13 InSpeciesTypeBond

An **InSpeciesTypeBond** object defines a bond existing within a speciesType. The bond therefore exists in every species that references the speciesType.

**InSpeciesTypeBond** is defined in Figure 7. It has two optional attributes, id and name, and two required attributes, bindingSite1 and bindingSite2. Since **InSpeciesTypeBond** is derived from **SBase**, it inherits the sboTerm and metaid attributes, as well as the optional child **Notes** and **Annotation** objects.

The binding relationship in an inSpeciesTypeBond is one-to-one (see Section 3.8.7 on page 14). The uniqueness of an inSpeciesTypeBond is ensured by the pair of referenced bindingSites. A speciesType cannot have two inSpeciesTypeBonds containing the same pair of bindingSites.

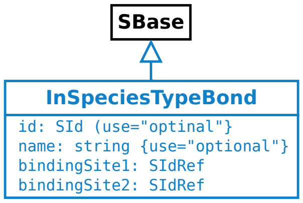

***Figure 7:*** *The definition of the* ***InSpeciesTypeBond*** *class*

#### *3.13.1 The id and name attributes*

The optional id attribute, of type SId, provides a way to identify an inSpeciesTypeBond. If present, the value of the id attribute must be unique within its direct parent speciesType.

**InSpeciesTypeBond** also has an optional name attribute of type string.

#### 3.13.2 *The* `bindingSite1` *and* `bindingSite2` *attributes*

**InSpeciesTypeBond** has two required attributes, `bindingSite1` and `bindingSite2`, both of type `SIdRef`, used to reference a pair of binding sites of the **InSpeciesTypeBond** object in a `speciesType`. The referenced identifiers of the binding sites can be the `id`s of the `speciesTypeInstance`s (binding sites), or the `id`s of the `speciesTypeComponent-Indexes` indexing the binding sites and the ultimately referenced components must be the **BindingSiteSpeciesType** objects. Obviously, `bindingSite1` and `bindingSite2` must not reference the same **BindingSiteSpeciesType** object.

### 3.14 Uniqueness of SpeciesType definitions

In some special cases, it may be possible to define a `speciesType` in multiple equivalent ways.

Figure 8 shows an example of a `speciesType` constructed in two different ways. The two "`st_x`" speciesTypes in the diagram can be the results of different reaction paths, but they are equivalent and define the same `speciesType`.

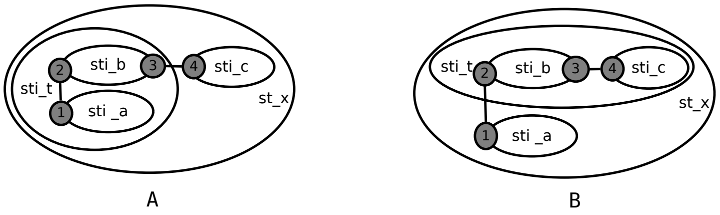

***Figure 8:*** *Different formats of the same* `speciesType`

Construct 1: The definition of speciesType "`st_x`" on the left (`A`) in Figure 8.

```
<multi:listOfSpeciesTypes>
   <multi:bindingSiteSpeciesType multi:id="st1" />
   <multi:bindingSiteSpeciesType multi:id="st2" />
   <multi:bindingSiteSpeciesType multi:id="st3" />
   <multi:bindingSiteSpeciesType multi:id="st4" />
   <multi:speciesType multi:id="st_a">
      <multi:listOfSpeciesTypeInstances>
         <multi:speciesTypeInstance multi:id="_1" multi:speciesType="st1" />
      </multi:listOfSpeciesTypeInstances>
   </multi:speciesType>
   <multi:speciesType multi:id="st_b">
      <multi:listOfSpeciesTypeInstances>
         <multi:speciesTypeInstance multi:id="_2" multi:speciesType="st2" />
         <multi:speciesTypeInstance multi:id="_3" multi:speciesType="st3" />
      </multi:listOfSpeciesTypeInstances>
   </multi:speciesType>
   <multi:speciesType multi:id="st_c">
      <multi:listOfSpeciesTypeInstances>
         <multi:speciesTypeInstance multi:id="_4" multi:speciesType="st4" />
      </multi:listOfSpeciesTypeInstances>
   </multi:speciesType>
   <multi:speciesType multi:id="st_t">
      <multi:listOfSpeciesTypeInstances>
         <multi:speciesTypeInstance multi:id="sti_a" multi:speciesType="st_a" />
         <multi:speciesTypeInstance multi:id="sti_b" multi:speciesType="st_b" />
      </multi:listOfSpeciesTypeInstances>
      <multi:listOfInSpeciesTypeBonds>
         <multi:inSpeciesTypeBond multi:bindingSite1="_1" multi:bindingSite2="_2" />
      </multi:listOfInSpeciesTypeBonds>
```

```
  </multi:speciesType>
  <multi:speciesType multi:id="st_x">
    <multi:listOfSpeciesTypeInstances>
      <multi:speciesTypeInstance multi:id="sti_t" multi:speciesType="st_t" />
      <multi:speciesTypeInstance multi:id="sti_c" multi:speciesType="st_c" />
    </multi:listOfSpeciesTypeInstances>
    <multi:listOfInSpeciesTypeBonds>
      <multi:inSpeciesTypeBond multi:bindingSite1="_3" multi:bindingSite2="_4" />
    </multi:listOfInSpeciesTypeBonds>
  </multi:speciesType>
</multi:listOfSpeciesTypes>
```

Construct 2: The definition of speciesType "`st_x`" on the right (B) in Figure 8 on the preceding page.

```
<multi:listOfSpeciesTypes>
  <multi:bindingSiteSpeciesType multi:id="st1" />
  <multi:bindingSiteSpeciesType multi:id="st2" />
  <multi:bindingSiteSpeciesType multi:id="st3" />
  <multi:bindingSiteSpeciesType multi:id="st4" />
  <multi:speciesType multi:id="st_a">
    <multi:listOfSpeciesTypeInstances>
      <multi:speciesTypeInstance multi:id="_1" multi:speciesType="st1" />
    </multi:listOfSpeciesTypeInstances>
  </multi:speciesType>
  <multi:speciesType multi:id="st_b">
    <multi:listOfSpeciesTypeInstances>
      <multi:speciesTypeInstance multi:id="_2" multi:speciesType="st2" />
      <multi:speciesTypeInstance multi:id="_3" multi:speciesType="st3" />
    </multi:listOfSpeciesTypeInstances>
  </multi:speciesType>
  <multi:speciesType multi:id="st_c">
    <multi:listOfSpeciesTypeInstances>
      <multi:speciesTypeInstance multi:id="_4" multi:speciesType="st4" />
    </multi:listOfSpeciesTypeInstances>
  </multi:speciesType>
  <multi:speciesType multi:id="st_t">
    <multi:listOfSpeciesTypeInstances>
      <multi:speciesTypeInstance multi:id="sti_b" multi:speciesType="st_b" />
      <multi:speciesTypeInstance multi:id="sti_c" multi:speciesType="st_c" />
    </multi:listOfSpeciesTypeInstances>
    <multi:listOfInSpeciesTypeBonds>
      <multi:inSpeciesTypeBond multi:bindingSite1="_3" multi:bindingSite2="_4" />
    </multi:listOfInSpeciesTypeBonds>
  </multi:speciesType>
  <multi:speciesType multi:id="st_x">
    <multi:listOfSpeciesTypeInstances>
      <multi:speciesTypeInstance multi:id="sti_a" multi:speciesType="st_a" />
      <multi:speciesTypeInstance multi:id="sti_t" multi:speciesType="st_t" />
    </multi:listOfSpeciesTypeInstances>
    <multi:listOfInSpeciesTypeBonds>
      <multi:inSpeciesTypeBond multi:bindingSite1="_1" multi:bindingSite2="_2" />
    </multi:listOfInSpeciesTypeBonds>
  </multi:speciesType>
</multi:listOfSpeciesTypes>
```

This kind of ambiguity cannot be avoided for `speciesType`s involving more than two subcomponents connected by `inSpeciesTypeBond`s. `A` and `B` in Figure 8 on the previous page can be products of different association reactions. It is up to the modeler (parser) to identify whether the two `speciesType`s such as those in the example above are identical.

### 3.15 Extended Species

A `species` in SBML Level 3 Core refers to a pool of entities. A `species` in the Multi package is extended from a pool to a template or pattern to which multiple pools may map to. An extended `species` can reference a `speciesType` that provides the backbone for the `species` such as `component`s (including `binding sites`) and `speciesFeatureType`s. When referencing a `speciesType`, a `species` can be further defined with regard to the binding statuses of its `outwardBindingSites` and the `speciesFeature`s. With the options to have variable values selected, such as "`either`" for the `bindingStatus` attribute and multiple `possibleSpeciesFeatureValue`s for a `speciesFeature`, an extended `species` can work as a template or pattern how `species` participate in `reaction`s.

The extension of the **Species** class is illustrated in Figure 9. It has a new optional attribute `speciesType`, and two extra optional **ListOfOutwardBindingSites** and **ListOfSpeciesFeatures** children. A `species` may have a **ListOfOutwardBindingSites** element and/or a **ListOfSpeciesFeatures** element only when its `speciesType` attribute has been defined. A `species` must have its `speciesType` attribute defined when it has a **ListOfOutwardBindingSites** element and/or a **ListOfSpeciesFeatures** element. The relationship among the elements of a **ListOfOutwardBindingSites** or **ListOfSpeciesFeatures** object is "and".

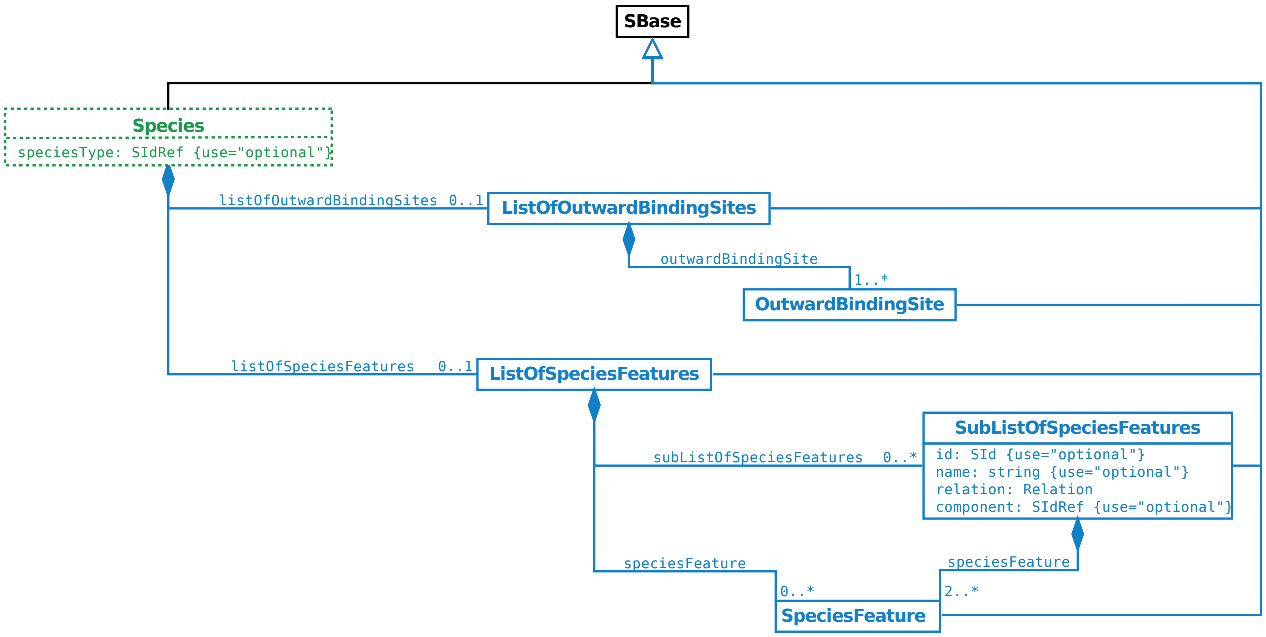

***Figure 9:*** *The extension of the* ***Species*** *class*

#### 3.15.1 *The* `speciesType` *attribute*

The optional attribute `speciesType`, of type `SIdRef`, references a **SpeciesType** object.

#### 3.15.2 *ListOfOutwardBindingSites*

**ListOfOutwardBindingSites** is defined in Figure 9 and is extended from the **ListOf** class. A `listOfOutwardBindingSites` can only be defined when the `speciesType` attribute is defined. If present, it must have one or more **OutwardBindingSite** children. Since **ListOfOutwardBindingSites** is derived from **SBase** through **ListOf**, it inherits the `sboTerm` and `metaid` attributes, as well as the optional child **Notes** and **Annotation** objects.

☞ *Note:*
*The* `listOfOutwardBindingSites` *of a* `species` *is not necessary to list all the* `outwardBindingSites` *(the binding*

*sites not involved in any* inSpeciesTypeBond*) defined by the referenced* speciesType*. If an* outwardBindingSite *is not listed in the* listOfOutwardBindingSites*, the value of its* bindingStatus *is "*either*". In other words, the binding site is in a "don't care" state.*

#### 3.15.3 ListOfSpeciesFeatures

**ListOfSpeciesFeatures** is defined in Figure 9 on the preceding page and is extended from the **ListOf** class. A listOfSpeciesFeatures can only be defined when the speciesType attribute is defined. If present, it must have one or more children. A child can be a **SpeciesFeature**, or a **SubListOfSpeciesFeatures** object.

☞ *Note:*
*The* listOfSpeciesFeatures *of a* species *does not have to cover all the* speciesFeature*s corresponding to all* speciesFeatureType*s (see Section 3.9 on page 14) of every* component *defined by the referenced* speciesType*. If a* speciesFeatureType *is defined and there is no* speciesFeature *explicitly referencing it, the* species *has an implicit* speciesFeature *having all the* listOfPossibleSpeciesFeatureValues *and "*or*" relationships between them. In other words, the implicit* speciesFeature *has a "don't care" state.*

Since **ListOfSpeciesFeatures** is derived from **SBase** through **ListOf**, it inherits the sboTerm and metaid attributes, as well as the optional child **Notes** and **Annotation** objects.

The example at Section 3.18.7 on page 27 illustrates the usage of the **ListOfSpeciesFeatures** class.

### 3.16 OutwardBindingSite

**OutwardBindingSite** is defined in Figure 10. It has two optional attributes, id and name, and two required attributes, bindingStatus and component. A binding site not involved in any **InSpeciesTypeBond** object in the speciesType referenced by a species is an outwardBindingSite. Since **OutwardBindingSite** is derived from **SBase**, it inherits the sboTerm and metaid attributes, as well as the optional child **Notes** and **Annotation** objects.

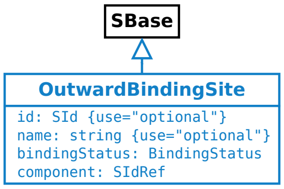

***Figure 10:*** *The definition of the* ***OutwardBindingSite*** *class*

#### 3.16.1 The id and name attributes

The optional id attribute, of type SId, can serve to provide a way to identify an outwardBindingSite. If present, the value must be unique within the species. **OutwardBindingSite** also has an optional name attribute of type string.

#### 3.16.2 The bindingStatus attribute

The bindingStatus attribute takes a value of type **BindingStatus**.

#### 3.16.3 The component attribute

The component attribute, of type SIdRef, references a component which ultimately references a **BindingSiteSpeciesType** object. The attribute value must be the identifier of a **SpeciesTypeInstance**, **SpeciesTypeComponentIndex** or **SpeciesType** object. An outwardBindingSite cannot be a binding site referenced by any inSpeciesTypeBond in the species.

There are three scenarios for the component attribute to have its value as an identifier of a **SpeciesType**, **SpeciesTypeInstance**, or **SpeciesTypeComponentIndex** object.

(1) When a species references a bindingSiteSpeciesType, the component attribute of its outwardBindingSite can only have value of the referenced speciesType's id.

(2) When a species references a speciesType with a speciesTypeInstance being a binding site (have an id of **BindingSiteSpeciesType** as its speciesType attribute) and the id of the speciesTypeInstance can identify the binding site within the speciesType (referenced by the species) unambiguously, and therefore, an outwardBindingSite of the species can have its component attribute with value of a speciesTypeInstance's id.

(3) When a species references a speciesType with a speciesTypeInstance being a binding site (directly or indirectly) and id of the speciesTypeInstance can NOT identify the binding site without ambiguity, an id of **SpeciesTypeComponentIndex** can be used as the value of the component attribute for an outwardBindingSite of the species.

#### 3.16.4 Example

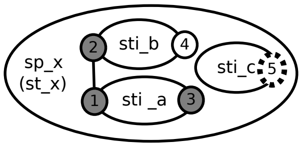

***Figure 11:** An example of* ***OutwardBindingSite***

Figure 11 illustrates the usage of the **OutwardBindingSite** class. Species "sp_x" references speciesType "st_x", which has three speciesTypeInstances "sti_a", "sti_b" and "sti_c". SpeciesTypeInstance "sti_a" has bindingSites "_1" and "_3", speciesTypeInstance "sti_b" has bindingSites "_2" and "_4", and speciesTypeInstance "sti_c" has bindingSite "_5". The inSpeciesTypeBond in "st_x" involves two bindingSites "_1" and "_2". The other three bindingSites, "_3", "_4" and "_5", in the species "sp_x" are outwardBindingSites. The outwardBindingSite "_3" is "bound" (filled circle with solid line in the diagram), the outwardBindingSite "_4" is "unbound" (empty circle with solid line) and the outwardBindingSite "_5" has binding status "either" (empty circle with dotted line). The corresponding SBML code would be as follows:

```
<multi:listOfSpeciesTypes>
   <multi:bindingSiteSpeciesType multi:id="st_1" />
   <multi:bindingSiteSpeciesType multi:id="st_2" />
   <multi:bindingSiteSpeciesType multi:id="st_3" />
   <multi:bindingSiteSpeciesType multi:id="st_4" />
   <multi:bindingSiteSpeciesType multi:id="st_5" />
   <multi:speciesType multi:id="st_a">
      <multi:listOfSpeciesTypeInstances>
         <multi:speciesTypeInstance multi:id="_1" multi:speciesType="st_1" />
         <multi:speciesTypeInstance multi:id="_3" multi:speciesType="st_3" />
      </multi:listOfSpeciesTypeInstances>
   </multi:speciesType>
   <multi:speciesType multi:id="st_b">
      <multi:listOfSpeciesTypeInstances>
         <multi:speciesTypeInstance multi:id="_2" multi:speciesType="st_2" />
         <multi:speciesTypeInstance multi:id="_4" multi:speciesType="st_4" />
      </multi:listOfSpeciesTypeInstances>
   </multi:speciesType>
   <multi:speciesType multi:id="st_c">
      <multi:listOfSpeciesTypeInstances>
         <multi:speciesTypeInstance multi:id="_5" multi:speciesType="st_5" />
      </multi:listOfSpeciesTypeInstances>
```

```
   </multi:speciesType>
   <multi:speciesType multi:id="st_x">
      <multi:listOfSpeciesTypeInstances>
         <multi:speciesTypeInstance multi:id="sti_a" multi:speciesType="st_a" />
         <multi:speciesTypeInstance multi:id="sti_b" multi:speciesType="st_b" />
         <multi:speciesTypeInstance multi:id="sti_c" multi:speciesType="st_c" />
      </multi:listOfSpeciesTypeInstances>
      <multi:listOfInSpeciesTypeBonds>
         <multi:inSpeciesTypeBond  multi:bindingSite1="_1" multi:bindingSite2="_2" />
      </multi:listOfInSpeciesTypeBonds>
   </multi:speciesType>
</multi:listOfSpeciesTypes>
<listOfSpecies>
   <species id="sp_x" multi:speciesType="st_x">
      <multi:listOfOutwardBindingSites>
         <multi:outwardBindingSite multi:component="_3" multi:bindingStatus="bound" />
         <multi:outwardBindingSite multi:component="_4" multi:bindingStatus="unbound" />
         <multi:outwardBindingSite multi:component="_5" multi:bindingStatus="either" />
      </multi:listOfOutwardBindingSites>
   </species>
</listOfSpecies>
```

### 3.17 SubListOfSpeciesFeatures

**SubListOfSpeciesFeatures** is defined in Figure 9 on page 22, and is extended from the **ListOf** class. A **SubListOfSpeciesFeatures** object must have two or more **SpeciesFeature** children. Since **SubListOfSpeciesFeatures** is derived from **SBase** through **ListOf**, it inherits the `sboTerm` and `metaid` attributes, as well as the optional child **Notes** and **Annotation** objects.

#### 3.17.1 *The* `id` *and* `name` *attributes*

The optional `id` attribute, of type `SId`, can serve to provide a way to identify a **SubListOfSpeciesFeatures** object. If present, its value must be unique within the `species`. **SubListOfSpeciesFeatures** also has an optional `name` attribute of type `string`.

#### 3.17.2 *The* `relation` *attribute*

**SubListOfSpeciesFeatures** has a required attribute `relation`, of type `Relation`, to define the logic relationship among its children. If any `speciesFeature` involved in a `subListOfSpeciesFeatures` references a `speciesFeatureType` with "`occur > 1`", the `subListOfSpeciesFeatures` can only have the value "`and`" for its `relation` attribute.

#### 3.17.3 *The* `component` *attribute*

The optional `component` attribute, of type `SIdRef`, can be used to indicate which **`component`** in a `species` the **SubListOfSpeciesFeatures** object belongs to. It is required when a `speciesFeature` contained in this `subListOfSpeciesFeatures` cannot be identified only based on its `speciesFeatureType` attribute.

### 3.18 SpeciesFeature

**SpeciesFeature** is defined in Figure 12 on the following page. It has three optional attributes, `id`, `name` and `component`, and two required attributes, `speciesFeatureType` and `occur`, and a required child `listOfSpeciesFeatureValues`. Since **SpeciesFeature** is derived from **SBase**, it inherits the `sboTerm` and `metaid` attributes, as well as the optional child **Notes** and **Annotation** objects. **SpeciesFeature** serves to define the state of a `component` in a `species` by selecting values from the `listOfPossibleSpeciesFeatureValues` of the referenced `speciesFeatureType`.

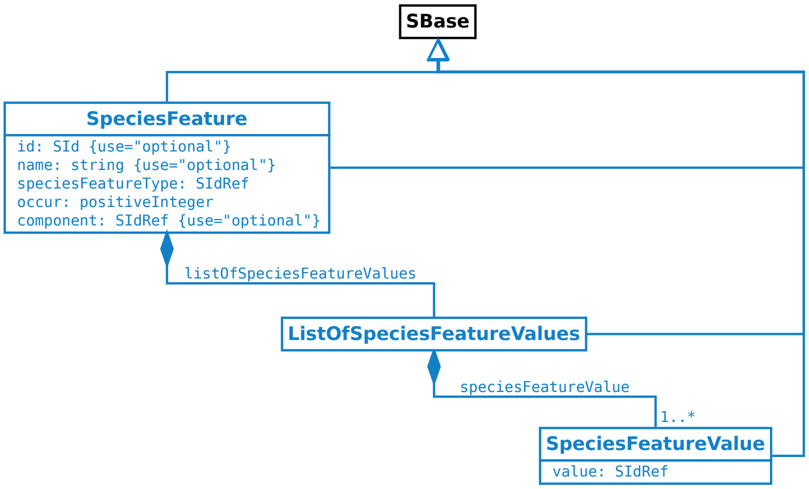

***Figure 12:*** *The definitions of the* ***SpeciesFeature*** *class and the* ***SpeciesFeatureValue*** *class*

#### *3.18.1 The* `id` *and* `name` *attributes*

The optional `id` attribute, of type `SId`, can serve to provide a way to identify a `speciesFeature`. If present, the value must be unique within the `species`. **SpeciesFeature** also has an optional `name` attribute of type `string`.

#### *3.18.2 The* `speciesFeatureType` *attribute*

**SpeciesFeature** has a required attribute `speciesFeatureType`, of type `SIdRef`, used to reference a `speciesFeatureType`.

#### *3.18.3 The* `occur` *attribute*

**SpeciesFeature** has a required attribute `occur`, of type of `positiveInteger`, used to define the number of instances of the referenced `speciesFeatureType`.

The value of the `occur` attribute cannot be larger than the `occur` of the referenced `speciesFeatureType`. When a `speciesFeatureType` has multiple instances (`speciesFeatureType`'s `occur` > "1"), the `speciesFeature`'s `occur` attribute provides a way for a `species` to define the instances of the `speciesFeatureType` differently.

For example, in a `speciesType`, `speciesFeatureType` "`ftA`" has `occur`="2" and two `possibleSpeciesFeatureValues` "`fva1`" and "`fva2`". A `species` referencing the `speciesType` can be defined to have two `speciesFeatures` "`sfA1`" and "`sfA2`" both referencing "`ftA`". The `speciesFeature` "`sfA1`" has `occur`="1" and its value is "`fva1`". The `speciesFeature` "`sfA2`" has `occur`="1" and its value is "`fva2`".

If the `occur` of a `speciesFeature` is less than the `occur` of the referenced `speciesFeatureType`, the rest of the unspecified instances of the `speciesFeatureType` are in *don't care* state which means that the value of an unspecified instance can be any from the `listOfPossibleSpeciesFeatureValues`.

For example, in a `speciesType`, a speciesFeatureType "`phosphorylation`" has two `possibleSpeciesFeatureValues` "`phosphorylated`" and "`unphosphorylated`" and the `occur` is "5". A `species` referencing the `speciesType` can be defined to have a `speciesFeature` of the "`phosphorylation`" with the value of "`phosphorylated`" and the `occur` of "1". Then, the `species` is a pattern `species` with at least one "`phosphorylated`" site (the other four "`phosphorylation`" sites are in *don't care* state). This pattern `species` can be mapped by anyone of the *fully defined* `species` (see Section 3.19 on page 29) of the same type and with any of "1" to "5" phosphorylated sites.

#### 3.18.4 The `component` attribute

The optional `component` attribute, of type `SIdRef`, can be used to indicate which **`component`** in a `species` the `speciesFeature` belongs to. It is required when the `component` cannot be identified only based on the `speciesFeatureType` attribute.

#### 3.18.5 ListOfSpeciesFeatureValues

**ListOfSpeciesFeatureValues** is defined in Figure 12 on the preceding page, and is extended from the **ListOf** class. A **ListOfSpeciesFeatureValues** object must have one or more **SpeciesFeatureValue** children. If a `listOfSpecies-Features` has multiple `speciesFeatureValue`s, the interpretation of the relationship between them is "`or`". Since **ListOfSpeciesFeatureValues** is derived from **SBase** through **ListOf**, it inherits the `sboTerm` and `metaid` attributes, as well as the optional child **Notes** and **Annotation** objects.

#### 3.18.6 SpeciesFeatureValue

**SpeciesFeatureValue** is defined in Figure 12 on the previous page. A `speciesFeatureValue` serves to specify a value for a `speciesFeature` to select from the `listOfPossibleSpeciesFeatureValues` defined in the referenced `speciesFeatureType`. The **SpeciesFeatureValue** class has only one attribute `value`, of type `SIdRef`, used to reference a **PossibleSpeciesFeatureValue** object. Since **SpeciesFeatureValue** is derived from **SBase**, it inherits the `sboTerm` and `metaid` attributes, as well as the optional child **Notes** and **Annotation** objects.

#### 3.18.7 Example

Figure 13 on the following page is an example `speciesType` to illustrate the usage of the **ListOfSpeciesFeatures**, **Sub-ListOfSpeciesFeatures** and **SpeciesFeature** classes. `SpeciesType` "`st_X`" has `speciesTypeInstance` "`sti_A`" with `speciesFeatureType` "`fA`", and `speciesTypeInstance` "`sti_B`" with `speciesFeatureType`s "`fB1`" and "`fB2`". The speciesFeatureType "`fA`" has two `possibleSpeciesFeatureValue`s "`v1`" and "`v2`". The `speciesFeatureType` "`fB1`" has "`v3`" and "`v4`", and "`fB2`" has "`v5`" and "`v6`". Here are several ways to construct the `listOfSpeciesFeatures` of a `species` referencing the `speciesType` "`st_X`":

- `Species` "`sp_X1`": `listOfSpeciesFeatures`("`fA`"="`v1`", "`fB1`"="`v3`", "`fB2`"="`v5`") represents a state:
  "`[fA=v1] AND [fB1=v3] AND [fB2=v5]`"
- `Species` "`sp_X2`": `listOfSpeciesFeatures`("`fA`"="`v1`", "`fB1`"="`v3`") represents a state:
  "`[fA=v1] AND [fB1=v3] AND ([fB2=v5] OR fB2=v6)`"
  "`fB2`" has a value of *don't care*
- `Species` "`sp_X3`": `listOfSpeciesFeatures`(
  "`fA=v1`",
  `subListOfSpeciesFeatures` ("`fB1=v3`", "`fB2=v5`", `relation`="`not`")
  ) represents states:
  "`[fA=v1] and [fB1=v4] and [fB2=V5]`" or
  "`[fA=v1] and [fB1=v4] and [fB2=V6]`" or
  "`[fA=v1] and [fB1=v3] and [fB2=V6]`"

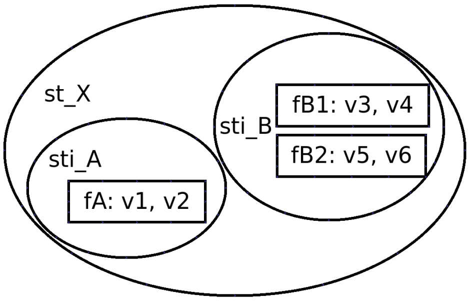

***Figure 13:*** *An example* `speciesFeatureType` *to illustrate the usage of* ***ListOfSpeciesFeatures****,* ***SubListOfSpecies-Features*** *and the* ***SpeciesFeature***

The SBML code for the `speciesTypes` "`st_A`", "`st_B`", and "`st_X`", and the `species` "`sp_X3`" can be as follows:

```
<multi:listOfSpeciesTypes>
  <multi:speciesType multi:id="st_A" ... >
    <multi:listOfSpeciesFeatureTypes>
      <multi:speciesFeatureType multi:id="fA" multi:occur="1">
        <multi:listOfPossibleSpeciesFeatureValues>
          <multi:possibleSpeciesFeatureValue multi:id="v1" />
          <multi:possibleSpeciesFeatureValue multi:id="v2" />
        </multi:listOfPossibleSpeciesFeatureValues>
      </multi:speciesFeatureType>
    </multi:listOfSpeciesFeatureTypes>
    ...
  </multi:speciesType>
  <multi:speciesType multi:id="st_B" ... >
    <multi:listOfSpeciesFeatureTypes>
      <multi:speciesFeatureType multi:id="fB1" multi:occur="1">
        <multi:listOfPossibleSpeciesFeatureValues>
          <multi:possibleSpeciesFeatureValue multi:id="v3" />
          <multi:possibleSpeciesFeatureValue multi:id="v4" />
        </multi:listOfPossibleSpeciesFeatureValues>
      </multi:speciesFeatureType>
      <multi:speciesFeatureType multi:id="fB2" multi:occur="1">
        <multi:listOfPossibleSpeciesFeatureValues>
          <multi:possibleSpeciesFeatureValue multi:id="v5" />
          <multi:possibleSpeciesFeatureValue multi:id="v6" />
        </multi:listOfPossibleSpeciesFeatureValues>
      </multi:speciesFeatureType>
    </multi:listOfSpeciesFeatureTypes>
    ...
  </multi:speciesType>
  <multi:speciesType multi:id="st_X" ... >
    <multi:listOfSpeciesTypeInstances>
      <multi:speciesTypeInstance multi:id="sti_A" multi:speciesType="st_A" />
      <multi:speciesTypeInstance multi:id="sti_B" multi:speciesType="st_B" />
    </multi:listOfSpeciesTypeInstances>
    ...
  </multi:speciesType>
  ...
</multi:listOfSpeciesTypes>
<listOfSpecies>
  <species id="sp_X3" name="X3" multi:speciesType="st_X" ... >
    <multi:listOfSpeciesFeatures>
      <multi:speciesFeature multi:speciesFeatureType="fA" multi:occur="1">
        <multi:listOfSpeciesFeatureValues>
          <multi:speciesFeatureValue multi:value="v1" />
        </multi:listOfSpeciesFeatureValues>
      </multi:speciesFeature>
      <multi:subListOfSpeciesFeatures multi:relation="not">
```

```
        <multi:speciesFeature multi:speciesFeatureType="fB1" multi:occur="1">
          <multi:listOfSpeciesFeatureValues>
            <multi:speciesFeatureValue multi:value="v3" />
          </multi:listOfSpeciesFeatureValues>
        </multi:speciesFeature>
        <multi:speciesFeature multi:speciesFeatureType="fB2" multi:occur="1">
          <multi:listOfSpeciesFeatureValues>
            <multi:speciesFeatureValue multi:value="v5" />
          </multi:listOfSpeciesFeatureValues>
        </multi:speciesFeature>
      </multi:subListOfSpeciesFeatures>
    </multi:listOfSpeciesFeatures>
  </species>
  ...
</listOfSpecies>
```

### 3.19 *Fully defined* species and mapping to *pattern* species

An extended **Species** object functions as a template or a pattern which allows multiple pools of entities to map to it. A species is *fully defined* if there is only one pool mapping to it. A *fully defined* species can be considered the same as an SBML core species, and can be initialized with the initialAmount attribute, or the initialConcentration attribute, or via an **InitialAssignment** object. In the Multi package, a species is *fully defined* if the following conditions are fulfilled:

- All outwardBindingSites must be free (bindingStatus="unbound"), since "bound" sites imply that there is a non-specified binding partner.
- Each speciesFeature occurrence can only have one speciesFeatureValue, and every occurrence of every speciesFeatureType of every component of the referenced speciesType must be referenced by exactly one speciesFeature occurrence.
- Only "and" values are allowed for the relation attributes of the **SubListOfSpeciesFeatures** objects.
- Only one single **SpeciesFeatureValue** object is allowed for any speciesFeature.
- The referenced compartment cannot be a compartment type, which means the value of the isType attribute of the referenced compartment can only be "false".

The mapping from a *fully defined* species to a *pattern* species is implicit and can be inferred from the structure of the species. For example, a speciesType "stA" has one speciesFeatureType with two possibleSpeciesFeatureValues "v1" and "v2". The species "spA1" references "stA" and has the speciesFeature with the value of "v1". Another species "spA" also references "stA" and has no speciesFeature explicitly defined. Thus, the species "spA1" is a fully defined species and can map to the *pattern* species "spA" because species "spA" has an implicit speciesFeature which can take either value "v1" or value "v2" (see the note in Section 3.15.3 on page 23).

### 3.20 Reaction

**Reaction** itself in the Multi package is not extended, but it allows to use the Multi **Species** objects to construct reactions. The **Reaction** class in the Multi package cannot only define the relations among pools (SBML core species), but also the relations among patterns (Multi extended species). Several related classes including **SimpleSpeciesReference** and **SpeciesReference** are extended to handle some requirements specific to the Multi package. A new class, **IntraSpeciesReaction**, is derived from **Reaction** to explicitly define those reactions within the same **Species** object.

The changes under the **Reaction** class in the Multi package are illustrated in Figure 14 on the following page.

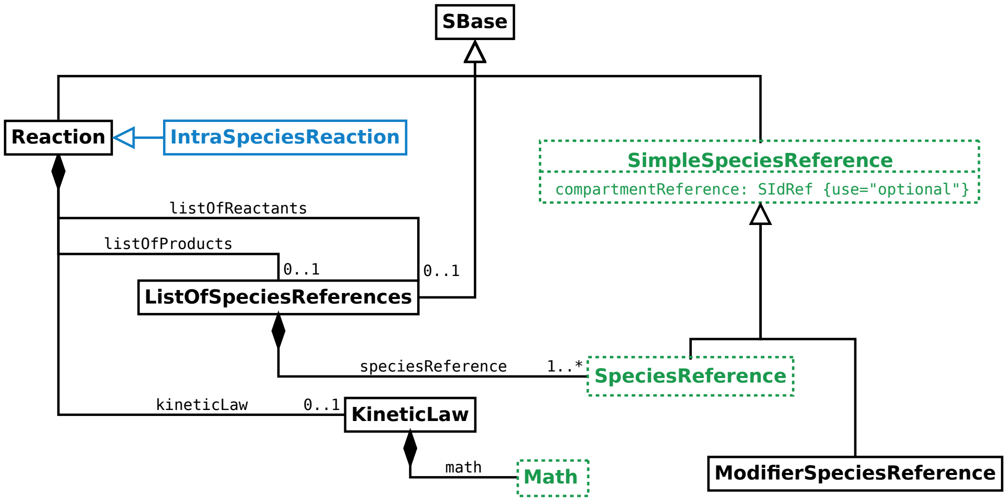

***Figure 14:*** *The changes under the* ***Reaction*** *class including* ***IntraSpeciesReaction****,* ***SimpleSpeciesReference****,* ***SpeciesReference*** *and* ***Math***

### 3.21 IntraSpeciesReaction

**IntraSpeciesReaction** is derived from **Reaction** for the `reaction`s happening within a `species` (see the example "`Extended Reaction class`" at page 23 of the slides at HARMONY 2013 [Zhang and Meier-Schellersheim (2013b)]).

A particular `reaction` may happen within a `species` as an `intraSpeciesReaction` if the following conditions are fulfilled:

- The `reaction` is either an association `reaction` or a dissociation `reaction`.
- If it is an association `reaction`, each of the two reactant `species` has at least one `outwardBindingSite` free ("`unbound`").
- If it is a dissociation `reaction`, each of the two product `species` has at least one `outwardBindingSite` free ("`unbound`").

☞ *Note:*
*Technically, transformations are reactions only happening in one* `species`*. They do not have the ambiguity and, therefore, do not have to be defined as* `intraSpeciesReactions`.

### 3.22 Extended SimpleSpeciesReference

The **SimpleSpeciesReference** class is extended with a new optional attribute `compartmentReference`, of type `SIdRef`, to reference a `compartmentReference`. The `compartmentReference` attribute can serve to indicate in which sub-compartment an object of a class (**SpeciesReference** or **ModifierSpeciesReference**) inheriting **SimpleSpeciesReference** is located.

This example illustrates the use of the `compartmentReference` attribute in `simpleSpeciesReference`s. The situation described here could correspond to interactions among species located on two adjacent membranes. A model has a composite `compartment` "`cc`" with two compartmentReferences "`cr1`" and "`cr2`", and both reference "`c`" subcomponents. `Species` "`spA`" references `compartment` "`c`", and `species` "`spAA`" references the composite `compartment` "`cc`". A `reaction` happens between two "`spA`" `species` from the two "`c`" `compartment`s and results in a cross-`compartment` product.

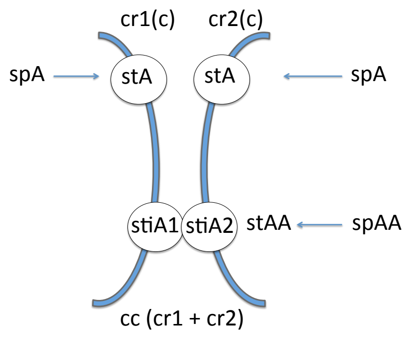

***Figure 15:*** *Reaction: spA(cr1) + spA(cr2) –> spAA*

The SBML code can be as follows:

```
<listOfCompartments>
   <compartment id="c" constant="true" multi:isType="true" />
   <compartment id="cc" constant="true" multi:isType="true">
      <multi:listOfCompartmentReferences>
         <multi:compartmentReference multi:id="cr1" multi:compartment="c" />
         <multi:compartmentReference multi:id="cr2" multi:compartment="c" />
      </multi:listOfCompartmentReferences>
   </compartment>
</listOfCompartments>
<multi:listOfSpeciesTypes>
   <multi:bindingSiteSpeciesType multi:id="stA" multi:compartment="c" />
   <multi:speciesType multi:id="stAA" multi:compartment="cc">
      <multi:listOfSpeciesTypeInstances>
         <multi:speciesTypeInstance multi:id="stiA1" multi:speciesType="stA"
            multi:compartmentReference="cr1" />
         <multi:speciesTypeInstance multi:id="stiA2" multi:speciesType="stA"
            multi:compartmentReference="cr2" />
      </multi:listOfSpeciesTypeInstances>
      <multi:listOfInSpeciesTypeBonds>
         <multi:inSpeciesTypeBond multi:bindingSite1="stiA1" multi:bindingSite2="stiA2" />
      </multi:listOfInSpeciesTypeBonds>
   </multi:speciesType>
</multi:listOfSpeciesTypes>
<listOfSpecies>
   <species id="spA" multi:speciesType="stA" compartment="c" ... />
   <species id="spAA" multi:speciesType="stAA" compartment="cc" ... />
</listOfSpecies>
<listOfReactions>
   <reaction id="reaction" ...>
      <listOfReactants>
         <speciesReference id="r1" species="spA" multi:compartmentReference="cr1" ... />
         <speciesReference id="r2" species="spA" multi:compartmentReference="cr2" ... />
      </listOfReactants>
      <listOfProducts>
         <speciesReference species="spAA" ... />
      </listOfProducts>
      ...
   </reaction>
   ...
</listOfReactions>
```

### 3.23 Extended SpeciesReference

The **SpeciesReference** class is extended from SBML Level 3 Core and can establish `component` mappings between the reactant `species` and the product `species` when the mappings cannot be inferred from the `id`s of the **SpeciesTypeInstance** objects. The **SpeciesReference** class has an optional **ListOfSpeciesTypeComponentMapsInProduct** child, as defined in Figure 16. Only a `reaction` `product` can contain the **ListOfSpeciesTypeComponentMapsInProduct** child and it is not necessary to store the mappings again in the `reactant`s.

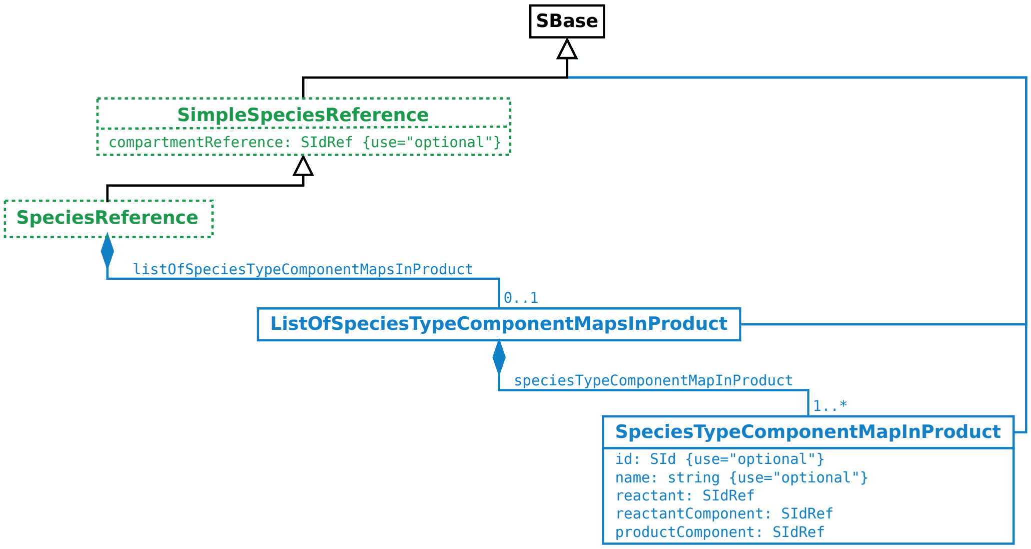

***Figure 16:*** *The extension of the **SpeciesReference** class*

#### *3.23.1 ListOfSpeciesTypeComponentMapsInProduct*

**ListOfSpeciesTypeComponentMapsInProduct** is defined in Figure 16, and is extended from the **ListOf** class. A **ListOfSpeciesTypeComponentMapsInProduct** object must have one or more **SpeciesTypeComponentMapInProduct** children. Since **ListOfSpeciesTypeComponentMapsInProduct** is derived from **SBase** through **ListOf**, it inherits the `sboTerm` and `metaid` attributes, as well as the optional child **Notes** and **Annotation** objects.

### 3.24 SpeciesTypeComponentMapInProduct

**SpeciesTypeComponentMapInProduct** is defined in Figure 16. It has two optional attributes, `id` and `name`, and three required attributes, `reactant`, `reactantComponent` and `productComponent`. Since **SpeciesTypeComponentMapInProduct** is derived from **SBase**, it inherits the `sboTerm` and `metaid` attributes, as well as the optional child **Notes** and **Annotation** objects.

A `speciesTypeComponentMapInProduct` defines the mapping between a `component` in a reactant and a `component` in a product. The identifications of a **`component`** and the `speciesReference` should be sufficient to identify the `component` in the context of a `reaction`. The attributes `reactant` and `reactantComponent` can identify the `component` in a `reactant`, and the `productComponent` attribute and the product storing the mapping information can identify the `component` in a `product`.

#### *3.24.1 The `id` and `name` attributes*

The optional `id` attribute, of type `SId`, can serve to provide a way to identify a `speciesTypeComponentMapInProduct`. If present, the value must be unique within the `reaction`. **SpeciesTypeComponentMapInProduct** also has an optional `name` attribute of type `string`.

#### 3.24.2 *The* `reactant` *attribute*

**SpeciesTypeComponentMapInProduct** has a required `reactant` attribute, of type `SIdRef`, to reference the `id` of a reactant `speciesReference` in a `reaction`.

#### 3.24.3 *The* `reactantComponent` *attribute*

**SpeciesTypeComponentMapInProduct** has a required `reactantComponent` attribute, of type `SIdRef`, to reference a `component` in a reactant `species`.

#### 3.24.4 *The* `productComponent` *attribute*

**SpeciesTypeComponentMapInProduct** has a required `productComponent` attribute, of type `SIdRef`, to reference a `component` in a product `species`.

### 3.25 `OutwardBindingSites` and `speciesFeatures` in *don't care* state in reaction `products`

An `outwardBindingSite` is in *don't care* state if its `bindingStatus` is "`either`" or is not specified (also see Section 3.15.2 on page 22). A `speciesFeature` is in *don't care* state if it is not specified in the referencing `species` (also see Section 3.18 on page 25).

For a `species` as a `product` in a `reaction`, if it has *don't care* `outwardBindingSites` or *don't care* `speciesFeatures`, the interpretation of the *don't care* is *don't change*. In a `product`, a *don't care* `outwardBindingSite` has the same `bindingStatus` as the mapped `outwardBindingSite` in the `reactant`, and a *don't care* `speciesFeature` has the same value as the mapped `speciesFeature` in the `reactant`.

### 3.26 Extended `ci` elements in Math objects

The Multi package extends the `ci` element in **Math** in **Reaction** with optional attributes `speciesReference` and `representationType`.

#### 3.26.1 *The* `speciesReference` *attribute*

The optional `speciesReference` attribute, of type `SIdRef`, can only be used when the content of the `ci` element is a `species` `id`, or when the content of the `ci` element is a `speciesFeature` `id`. The `speciesReference` attribute can identify which `species` is referenced in a `reaction`, and the `speciesReference` attribute must have a value of a `speciesReference` `id` within the same `reaction`.

If the `ci` content references a `species`' `id`, the `id` represents the concentration or amount of the `species`.

If the `ci` content references a `speciesFeature`'s `id`, the `id` represents the count of the `speciesFeature` instances with the `speciesFeatureValue` (also see Section 3.18.1 on page 26).

The example in Section 3.22 on page 30 can be further extended with a block of `kineticLaw` in the `reaction` to illustrate the use of the `speciesReference` attribute with a `species`' `id`.

```
<reaction id="reaction" ... >
   ...
   <kineticLaw>
      <math xmlns="http://www.w3.org/1998/Math/MathML">
         <apply>
            <times />
            <ci> k </ci>
            <ci multi:speciesReference="r1"> spA </ci>
            <ci multi:speciesReference="r2"> spA </ci>
         </apply>
      </math>
```

```
      <listOfLocalParameters>
         <localParameter id="k" value="0.1" ... />
      </listOfLocalParameters>
   </kineticLaw>
</reaction>
```

Two “spA” species are distinguished by the “r1” and “r2” speciesReferences respectively.

#### 3.26.2 The representationType attribute

The optional representationType attribute, of type **RepresentationType**, can only be used when the content of the ci element is a species’ id or a possibleSpeciesFeatureValue’s id. The representationType and speciesReference attributes can both be used for the same ci element at the same time.

The representationType attribute can only have the value of “sum” when the content of the ci is the id of a species. The interpretation of such a ci element is the total concentration or amount of all *fully defined* species (see Section 3.19 on page 29) mapping to the referenced pattern species.

The representationType attribute can have the value of numericValue when the content of the ci is the id of a possibleSpeciesFeatureValue and the speciesReference attribute must be defined. The interpretation of such a ci is the same as a ci element having a parameter which the possibleSpeciesFeatureValue links via its numericValue attribute.

The following example demonstrates the use of this attribute for “sum” of species concentrations.

```
   k1*Si/(k2+SUM(Si))
```

In this example, the reactant “Si” is a pattern species which may have multiple *fully defined* species mapping to it, for example species “S1”, “S2”, ..., “Sn”. “SUM(Si)” is a function to calculate the total concentration of all *fully defined* species mapping to “Si”. The product can be another pattern species “Pi”.

The SBML code for the math expression can be as follows:

```
<reaction id="r">
   <listOfReactants>
      <speciesReference species="Si" />
   </listOfReactants>
   <listOfProducts>
      <speciesReference species="Pi" />
   </listOfProducts>
   <kineticLaw>
      <math xmlns="http://www.w3.org/1998/Math/MathML">
         <apply>
            <divide>
               <apply>
                  <times />
                  <ci>Si</ci>
                  <ci>k1</ci>
               </apply>
               <apply>
                  <plus />
                  <ci>k2</ci>
                  <ci multi:representationType="sum">Si</ci>
               </apply>
            </divide>
         </apply>
      </math>
      <listOfLocalParameters>
         <localParameter id="k1" ... />
         <localParameter id="k2" ... />
      </listOfLocalParameters>
   </kineticLaw>
```

```
</reaction>
```

The math expressions for the individual species in the example can be:

```
For species S1:    k1*S1/(k2 + (S1 + S2 + ... + Sn))
For species S2:    k1*S2/(k2 + (S1 + S2 + ... + Sn))
...
For species Sn:    k1*Sn/(k2 + (S1 + S2 + ... + Sn))
```

### 3.27 Namespace scoping rules for identifiers

In the Multi package, as in SBML Level 3 Version 1 Core, the **Model** object contains the main components of an SBML model, such as species, compartments and reactions. The package defines new classes within a model and the scope of the identifiers of those new classes should be defined to prevent identifier collisions. In this section, we describe the scoping rules for all of the types and classes defined in Section 3.3 to Section 3.26 on pages 10–33.

1. The namespace for SId identifiers defined within a **Model** object used in the Multi package follows the same rules as those defined in SBML Level 3 Version 1 Core for plain **Model** objects. The scope of the identifiers is limited to the enclosing **Model** object.

2. The identifier of every **SpeciesType** and **PossibleSpeciesFeatureValue** object defined in the Multi package must be unique across the set of all identifiers in the **Model** object in which it is located.

3. The identifier of every **SpeciesTypeInstance**, **SpeciesTypeComponentIndex**, **InSpeciesTypeBond** and **SpeciesFeatureType** object defined in the Multi package must be unique across the set of all identifiers of the same class under the direct parent **SpeciesType** object in which it is located.

4. The identifier of every **SpeciesFeature** and **SubListOfSpeciesFeatures** object defined in the Multi package must be unique across the set of all identifiers in the **Species** object in which it is located.

5. The identifier, if defined, of every **CompartmentReference** object defined in the Multi package must be unique across the set of all identifiers in the **Compartment** object in which it is located.

## 4 Examples

This section contains examples employing the Multi package for SBML Level 3.

### 4.1 Example: Compartment, SpeciesType, and Species

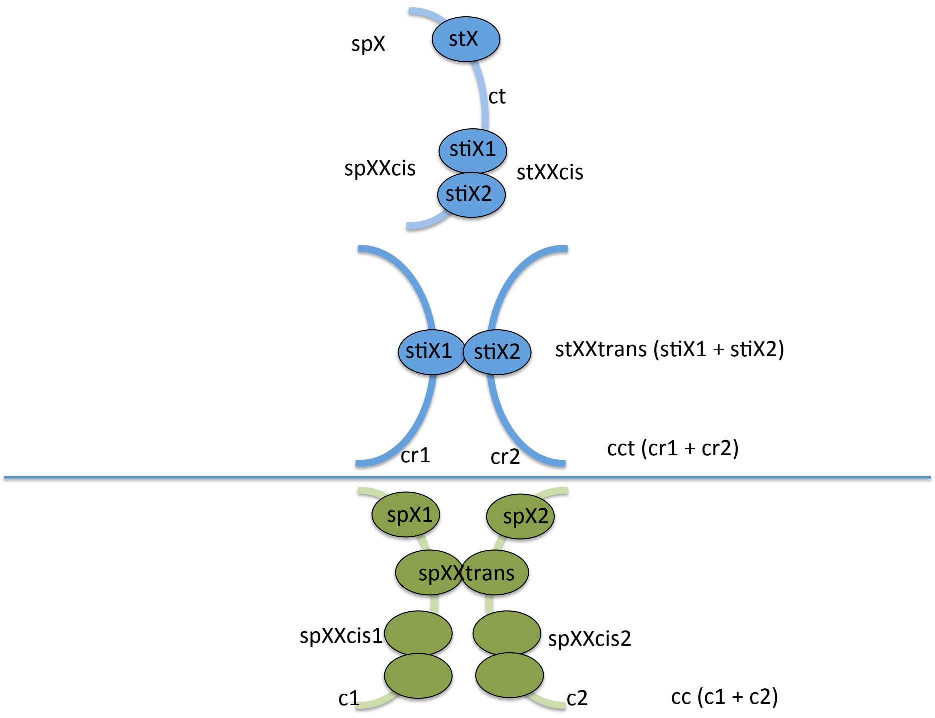

***Figure 17:*** *Diagram for an example of* ***Compartment****,* ***SpeciesType*** *and* ***Species***

Figure 17 shows an example illustrating the usages of and relations among the **Compartment**, **SpeciesType** and **Species** classes.

"`ct`" is a `compartment` type. "`cct`" is a composite `compartment` type with two `compartmentReference`s "`cr1`" and "`cr2`" both referencing "`ct`". "`c1`" is a "`not-a-type`" `compartment` and references "`ct`". Similarly, "`c2`" is also a "`not-a-type`" `compartment` and references "`ct`". "`cc`" is a composite "`not-a-type`" `compartment` composed of "`c1`" and "`c2`".

"`stX`" is a `speciesType` on the "`ct`" `compartment`. "`stXXcis`" is a `speciesType` on the "`ct`" `compartment`, and has two `speciesTypeInstances` "`stiX1`" and "`stX2`" both of that reference the "`stX`" speciesType. "`stXXtrans`" is a `speciesType` on the "`cct`" `compartment` with two `speciesTypeInstance`s "`stiX1`" and "`stiX2`" sitting in different sub-compartments.

"`spX`" is a `species` referencing `speciesType` "`stX`". "`spXXcis`" is a `species` referencing "`stXXcis`". "`spX1`" is a `species` referencing "`stX`" and sitting in the "`c1`" `compartment`. "`spX2`" is a `species` also referencing "`stX`", but sitting in "`c2`". "`spXXtrans`" is a `species` referencing "`stXXtrans`". "`spXXcis1`" is a `species` referencing "`stXXtrans`" and sitting in "`c1`"."`spXXcis1`" is a `species` referencing "`stXXtrans`" and sitting in "`c2`".

"spX1", "spX2", "spXXtrans", "spXXcis1" and "spXXcis2" are *fully defined* species (see Section 3.19 on page 29).

The SBML code can be as follows:

```
<!-- Note: this example is for the purpose of demonstration; it s not a complete model-->

<listOfCompartments>
   <compartment id="ct" multi:isType="true" />
   <compartment id="cct" multi:isType="true">
      <multi:listOfCompartmentReferences>
         <multi:compartmentReference multi:id="cr1" multi:compartment="ct" />
         <multi:compartmentReference multi:id="cr2" multi:compartment="ct" />
      </multi:listOfCompartmentReferences>
   </compartment>
   <compartment id="c1" multi:isType="false" multi:compartmentType="ct" />
   <compartment id="c2" multi:isType="false" multi:compartmentType="ct" />
   <compartment id="cc" multi:isType="false" multi:compartmentType="cct">
      <multi:listOfCompartmentReferences>
         <multi:compartmentReference multi:compartment="c1" />
         <multi:compartmentReference multi:compartment="c2" />
      </multi:listOfCompartmentReferences>
   </compartment>
</listOfCompartments>
<multi:listOfSpeciesTypes>
   <multi:bindingSiteSpeciesType multi:id="stX" multi:compartment="ct" />
   <multi:speciesType multi:id="stXXcis" multi:compartment="ct">
      <multi:listOfSpeciesTypeInstances>
         <multi:speciesTypeInstance multi:id="stiX1" multi:speciesType="stX" />
         <multi:speciesTypeInstance multi:id="stiX2" multi:speciesType="stX" />
      </multi:listOfSpeciesTypeInstances>
      <multi:listOfInSpeciesTypeBonds>
         <multi:inSpeciesTypeBond multi:bindingSite1="stiX1" multi:bindingSite2="stiX2" />
      </multi:listOfInSpeciesTypeBonds>
   </multi:speciesType>
   <multi:speciesType multi:id="stXXtrans" multi:compartment="cct">
      <multi:listOfSpeciesTypeInstances>
         <multi:speciesTypeInstance multi:id="stiX1" multi:speciesType="stX"
            multi:compartmentReference="cr1" />
         <multi:speciesTypeInstance multi:id="stiX2" multi:speciesType="stX"
            multi:compartmentReference="cr2" />
      </multi:listOfSpeciesTypeInstances>
      <multi:listOfInSpeciesTypeBonds>
         <multi:inSpeciesTypeBond multi:bindingSite1="stiX1" multi:bindingSite2="stiX2" />
      </multi:listOfInSpeciesTypeBonds>
   </multi:speciesType>
</multi:listOfSpeciesTypes>
<listOfSpecies>
   <species id="spX" multi:speciesType="stX" compartment="ct" />
   <species id="spXXcis" multi:speciesType="stXXcis" compartment="ct" />
   <species id="spX1" multi:speciesType="stX" compartment="c1" /> <!-- Fully defined -->
   <species id="spX2" multi:speciesType="stX" compartment="c2" /> <!-- Fully defined -->
   <species id="spXXtrans" multi:speciesType="stXXtrans" compartment="cc" /> <!-- Fully defined -->
   <species id="spXXcis1" multi:speciesType="stXXcis" compartment="c1" /> <!-- Fully defined -->
   <species id="spXXcis2" multi:speciesType="stXXcis" compartment="c2" /> <!-- Fully defined -->
</listOfSpecies>
```

### 4.2 *Simmune* example: the Ecad model

The *Simmune* toolset (http://go.usa.gov/QeH) has some example models including the published Ecad model [Angermann et al. (2012)]. The Ecad model describes the interactions between E-cadherin receptors that can associate either with other E-cadherin receptors within the same membrane (in "cis") or with E-cadherin receptors on adjacent membranes (in "trans"). This model is transformed into the SBML Level 3 format with use of the Multi package.

```
<?xml version="1.0" encoding="UTF-8"?>
<sbml xmlns="http://www.sbml.org/sbml/level3/version1/core" level="3" version="1"
   xmlns:multi="http://www.sbml.org/sbml/level3/version1/multi/version1" multi:required="true">

<!-- Note: this model has been validated against multi validation rules without errors -->

<model name="E-cadherin_mediated_adhesion">

<!-- Definitions -->
      <listOfUnitDefinitions>
         <unitDefinition id="litre_per_mole_per_sec">
            <listOfUnits>
               <unit kind="litre" exponent="1" scale="0" multiplier="1" />
               <unit kind="mole" exponent="-1" scale="0" multiplier="1" />
               <unit kind="second" exponent="-1" scale="0" multiplier="1" />
            </listOfUnits>
         </unitDefinition>
         <unitDefinition id="micron_square_per_sec">
            <listOfUnits>
               <unit kind="metre" exponent="2" scale="-6" multiplier="1" />
               <unit kind="second" exponent="-1" scale="0" multiplier="1" />
            </listOfUnits>
         </unitDefinition>
         <unitDefinition id="micrometre_per_sec">
            <listOfUnits>
               <unit kind="metre" exponent="1" scale="-6" multiplier="1" />
               <unit kind="second" exponent="-1" scale="0" multiplier="1" />
            </listOfUnits>
         </unitDefinition>
         <unitDefinition id="per_sec">
            <listOfUnits>
               <unit kind="second" exponent="-1" scale="0" multiplier="1" />
            </listOfUnits>
         </unitDefinition>
      </listOfUnitDefinitions>

<!-- Compartments -->
      <listOfCompartments>
         <compartment id="membrane" constant="true" multi:isType="true" />
         <compartment id="inter_membrane" constant="true" multi:isType="true">
            <multi:listOfCompartmentReferences>
               <multi:compartmentReference multi:id="m1" multi:compartment="membrane" />
               <multi:compartmentReference multi:id="m2" multi:compartment="membrane" />
            </multi:listOfCompartmentReferences>
         </compartment>
      </listOfCompartments>

<!-- SpeciesTypes -->
      <multi:listOfSpeciesTypes>

<!-- Ecad with cis-binding site and trans-binding site: -->
         <multi:bindingSiteSpeciesType multi:id="st_Cis_Interface" />
         <multi:bindingSiteSpeciesType multi:id="st_Trans_Interface" />
         <multi:speciesType multi:id="st_Ecad" multi:compartment="membrane">
            <multi:listOfSpeciesTypeInstances>
               <multi:speciesTypeInstance multi:id="cis" multi:speciesType="st_Cis_Interface" />
               <multi:speciesTypeInstance multi:id="trans" multi:speciesType="st_Trans_Interface" />
            </multi:listOfSpeciesTypeInstances>
         </multi:speciesType>

<!-- cis dimer: -->
         <multi:speciesType multi:id="st_Ecad_cis_dimer" multi:compartment="membrane">
            <multi:listOfSpeciesTypeInstances>
               <multi:speciesTypeInstance multi:id="Ecad1" multi:speciesType="st_Ecad" />
```

```
          <multi:speciesTypeInstance multi:id="Ecad_2" multi:speciesType="st_Ecad" />
        </multi:listOfSpeciesTypeInstances>
        <multi:listOfSpeciesTypeComponentIndexes>
          <multi:speciesTypeComponentIndex multi:id="Ecad1cis"
            multi:component="cis" multi:identifyingParent="Ecad1" />
          <multi:speciesTypeComponentIndex multi:id="Ecad2cis"
            multi:component="cis" multi:identifyingParent="Ecad2" />
          <multi:speciesTypeComponentIndex multi:id="Ecad1trans"
            multi:component="trans" multi:identifyingParent="Ecad1" />
          <multi:speciesTypeComponentIndex multi:id="Ecad2trans"
            multi:component="trans" multi:identifyingParent="Ecad2" />
        </multi:listOfSpeciesTypeComponentIndexes>
        <multi:listOfInSpeciesTypeBonds>
          <multi:inSpeciesTypeBond multi:bindingSite1="Ecad1cis"
            multi:bindingSite2="Ecad2cis" />
        </multi:listOfInSpeciesTypeBonds>
      </multi:speciesType>

<!-- trans dimer: -->
      <multi:speciesType multi:id="st_Ecad_trans_dimer" multi:compartment="inter_membrane">
        <multi:listOfSpeciesTypeInstances>
          <multi:speciesTypeInstance multi:id="Ecad1" multi:speciesType="st_Ecad"
            multi:compartmentReference="m1" />
          <multi:speciesTypeInstance multi:id="Ecad2" multi:speciesType="st_Ecad"
            multi:compartmentReference="m2" />
        </multi:listOfSpeciesTypeInstances>
        <multi:listOfSpeciesTypeComponentIndexes>
          <multi:speciesTypeComponentIndex multi:id="Ecad1trans"
            multi:component="trans" multi:identifyingParent="Ecad1" />
          <multi:speciesTypeComponentIndex multi:id="Ecad2trans"
            multi:component="trans" multi:identifyingParent="Ecad2" />
          <multi:speciesTypeComponentIndex multi:id="Ecad1cis"
            multi:component="cis" multi:identifyingParent="Ecad1" />
          <multi:speciesTypeComponentIndex multi:id="Ecad2cis"
            multi:component="cis" multi:identifyingParent="Ecad2" />
        </multi:listOfSpeciesTypeComponentIndexes>
        <multi:listOfInSpeciesTypeBonds>
          <multi:inSpeciesTypeBond multi:bindingSite1="Ecad1trans"
            multi:bindingSite2="Ecad2trans" />
        </multi:listOfInSpeciesTypeBonds>
      </multi:speciesType>

<!-- trimer: -->
      <multi:speciesType multi:id="st_Ecad_trimer" multi:compartment="inter_membrane">
        <multi:listOfSpeciesTypeInstances>
          <multi:speciesTypeInstance multi:id="Ecad1" multi:speciesType="st_Ecad"
            multi:compartmentReference="m1" />
          <multi:speciesTypeInstance multi:id="Ecad2" multi:speciesType="st_Ecad"
            multi:compartmentReference="m1" />
          <multi:speciesTypeInstance multi:id="Ecad3" multi:speciesType="st_Ecad"
            multi:compartmentReference="m2" />
        </multi:listOfSpeciesTypeInstances>
        <multi:listOfSpeciesTypeComponentIndexes>
          <multi:speciesTypeComponentIndex multi:id="Ecad1cis"
            multi:component="cis" multi:identifyingParent="Ecad1" />
          <multi:speciesTypeComponentIndex multi:id="Ecad1trans"
            multi:component="trans" multi:identifyingParent="Ecad1" />
          <multi:speciesTypeComponentIndex multi:id="Ecad2cis"
            multi:component="cis" multi:identifyingParent="Ecad2" />
          <multi:speciesTypeComponentIndex multi:id="Ecad2trans"
            multi:component="trans" multi:identifyingParent="Ecad2" />
          <multi:speciesTypeComponentIndex multi:id="Ecad3cis"
            multi:component="cis" multi:identifyingParent="Ecad3" />
          <multi:speciesTypeComponentIndex multi:id="Ecad3trans"
            multi:component="trans" multi:identifyingParent="Ecad3" />
        </multi:listOfSpeciesTypeComponentIndexes>
```

```xml
            <multi:listOfInSpeciesTypeBonds>
               <multi:inSpeciesTypeBond multi:bindingSite1="Ecad1cis"
                  multi:bindingSite2="Ecad2cis" />
               <multi:inSpeciesTypeBond multi:bindingSite1="Ecad1trans"
                  multi:bindingSite2="Ecad3trans" />
            </multi:listOfInSpeciesTypeBonds>
         </multi:speciesType>
      </multi:listOfSpeciesTypes>

<!-- Species -->
      <listOfSpecies>

<!-- free Ecad -->
         <species id="sp_Ecad_unbound" name="Ecad_unbound" compartment="membrane"
            hasOnlySubstanceUnits="false" boundaryCondition="false" constant="false"
            multi:speciesType="st_Ecad">
            <multi:listOfOutwardBindingSites>
               <multi:outwardBindingSite multi:component="cis"
                  multi:bindingStatus="unbound" />
               <multi:outwardBindingSite multi:component="trans"
                  multi:bindingStatus="unbound" />
            </multi:listOfOutwardBindingSites>
         </species>

<!-- Pattern species: Ecad trans unbnd -->
         <species id="sp_Ecad_trans_unbnd" name="Ecad_trans_unbnd" compartment="membrane"
            hasOnlySubstanceUnits="false" boundaryCondition="false" constant="false"
            multi:speciesType="st_Ecad">
            <multi:listOfOutwardBindingSites>
               <multi:outwardBindingSite multi:component="trans"
                  multi:bindingStatus="unbound" />
            </multi:listOfOutwardBindingSites>
         </species>

<!-- Pattern species: Ecad trans bnd -->
         <species id="sp_Ecad_trans_bnd" name="Ecad_trans_bnd" compartment="membrane"
            hasOnlySubstanceUnits="false" boundaryCondition="false" constant="false"
            multi:speciesType="st_Ecad">
            <multi:listOfOutwardBindingSites>
               <multi:outwardBindingSite multi:component="trans"
                  multi:bindingStatus="bound" />
            </multi:listOfOutwardBindingSites>
         </species>

<!-- Pattern species: Ecad all -->
         <species id="sp_Ecad_all" name="Ecad_all" compartment="membrane"
            hasOnlySubstanceUnits="false"
            boundaryCondition="false" constant="false" multi:speciesType="st_Ecad" />

<!-- Pattern species: Ecad cis unbnd -->
         <species id="sp_Ecad_cis_unbnd" name="Ecad_cis_unbnd" compartment="membrane"
            hasOnlySubstanceUnits="false" boundaryCondition="false" constant="false"
            multi:speciesType="st_Ecad">
            <multi:listOfOutwardBindingSites>
               <multi:outwardBindingSite multi:component="cis"
                  multi:bindingStatus="unbound" />
            </multi:listOfOutwardBindingSites>
         </species>

<!-- Pattern species: Ecad cis unbnd, trans bnd -->
         <species id="sp_Ecad_6" name="Ecad_6" compartment="membrane" hasOnlySubstanceUnits="false"
            boundaryCondition="false" constant="false" multi:speciesType="st_Ecad">
            <multi:listOfOutwardBindingSites>
               <multi:outwardBindingSite multi:component="cis"
                  multi:bindingStatus="unbound" />
               <multi:outwardBindingSite multi:component="trans"
```

```
            multi:bindingStatus="bound" />
      </multi:listOfOutwardBindingSites>
   </species>

<!-- Pattern species: Ecad cis bnd, trans unbnd -->
   <species id="sp_Ecad_7" name="Ecad_7" compartment="membrane" hasOnlySubstanceUnits="false"
      boundaryCondition="false" constant="false" multi:speciesType="st_Ecad">
      <multi:listOfOutwardBindingSites>
         <multi:outwardBindingSite multi:component="cis"
            multi:bindingStatus="bound" />
         <multi:outwardBindingSite multi:component="trans"
            multi:bindingStatus="unbound" />
      </multi:listOfOutwardBindingSites>
   </species>

<!-- Pattern species: Ecad cis dimer -->
   <species id="sp_Ecad_cis_dimer" name="Ecad_cis_dimer" compartment="membrane"
      hasOnlySubstanceUnits="false" boundaryCondition="false" constant="false"
      multi:speciesType="st_Ecad_cis_dimer" />

<!-- Pattern species: Ecad cis dimer: all trans bnd -->
   <species id="sp_EcadEcad_2" name="Ecad.Ecad_2" compartment="membrane"
      hasOnlySubstanceUnits="false" boundaryCondition="false" constant="false"
      multi:speciesType="st_Ecad_cis_dimer">
      <multi:listOfOutwardBindingSites>
         <multi:outwardBindingSite multi:component="Ecad1trans"
            multi:bindingStatus="bound" />
         <multi:outwardBindingSite multi:component="Ecad2trans"
            multi:bindingStatus="bound" />
      </multi:listOfOutwardBindingSites>
   </species>

<!-- Pattern species: Ecad trans dimer -->
   <species id="sp_EcadEcad_1" name="Ecad.Ecad_1" compartment="inter_membrane"
      hasOnlySubstanceUnits="false" boundaryCondition="false" constant="false"
      multi:speciesType="st_Ecad_trans_dimer" />

<!-- Pattern species: Ecad trans dimer: all cis bnd -->
   <species id="sp_Ecad_trans_dimer_2" name="Ecad_trans_dimer_2" compartment="inter_membrane"
      hasOnlySubstanceUnits="false" boundaryCondition="false" constant="false"
      multi:speciesType="st_Ecad_trans_dimer">
      <multi:listOfOutwardBindingSites>
         <multi:outwardBindingSite multi:component="Ecad1cis"
            multi:bindingStatus="bound" />
         <multi:outwardBindingSite multi:component="Ecad2cis"
            multi:bindingStatus="bound" />
      </multi:listOfOutwardBindingSites>
   </species>

<!-- Pattern species: Ecad True Trimer -->
   <species id="sp_Ecad_True_Trimer" compartment="inter_membrane" hasOnlySubstanceUnits="false"
      boundaryCondition="false" constant="false" multi:speciesType="st_Ecad_trimer">
      <multi:listOfOutwardBindingSites>
         <multi:outwardBindingSite multi:component="Ecad2trans"
            multi:bindingStatus="unbound" />
         <multi:outwardBindingSite multi:component="Ecad3cis"
            multi:bindingStatus="unbound" />
      </multi:listOfOutwardBindingSites>
   </species>

<!-- Pattern species: Ecad All Trimer -->
   <species id="sp_Ecad_All_Trimer" compartment="inter_membrane" hasOnlySubstanceUnits="false"
      boundaryCondition="false" constant="false" multi:speciesType="st_Ecad_trimer" />
</listOfSpecies>

<!-- Reactions -->
```

```
<listOfReactions>

<!-- cis association: -->
   <reaction id="rc_Cis_Association" name="Cis_Association" reversible="false" fast="false"
      compartment="membrane">
      <listOfReactants>
         <speciesReference id="Cis_Association_r1" species="sp_Ecad_6"
            stoichiometry="1" constant="false" />
         <speciesReference id="Cis_Association_r2" species="sp_Ecad_6"
            stoichiometry="1" constant="false" />
      </listOfReactants>
      <listOfProducts>
         <speciesReference species="sp_EcadEcad_2" constant="false" />
      </listOfProducts>
      <kineticLaw>
         <math xmlns="http://www.w3.org/1998/Math/MathML">
            <apply>
               <times />
               <ci> kon </ci>
               <ci multi:speciesReference="Cis_Association_r1"> sp_Ecad_6 </ci>
               <ci multi:speciesReference="Cis_Association_r2"> sp_Ecad_6 </ci>
            </apply>
         </math>
         <listOfLocalParameters>
            <localParameter id="kon" value="9000" units="litre_per_mole_per_sec" />
         </listOfLocalParameters>
      </kineticLaw>
   </reaction>

<!-- In species cis association: Here the model requires that the two interacting molecules
     are part of one connected complex already prior to the association. Since the necessary
     connectivity can only be mediated by the trans binding sites here, these sites must be
     bound to the subcomplex (not shown) linking the two interacting molecules.
     -->
   <multi:intraSpeciesReaction id="rc_Intra_Complex_Cis_Association"
    name="Intra-Complex_Cis_Association"
      reversible="false" fast="false" compartment="membrane">
      <listOfReactants>
         <speciesReference id="Intra_Complex_Cis_Association_r1" species="sp_Ecad_6"
            stoichiometry="1" constant="false" />
         <speciesReference id="Intra_Complex_Cis_Association_r2" species="sp_Ecad_6"
            stoichiometry="1" constant="false" />
      </listOfReactants>
      <listOfProducts>
         <speciesReference species="sp_EcadEcad_2" constant="false" />
      </listOfProducts>
      <kineticLaw>
         <math xmlns="http://www.w3.org/1998/Math/MathML">
            <apply>
               <times />
               <ci> kon </ci>
               <ci multi:speciesReference="Intra_Complex_Cis_Association_r1"> sp_Ecad_6 </ci>
            </apply>
         </math>
         <listOfLocalParameters>
            <localParameter id="kon" value="100" units="per_sec" />
         </listOfLocalParameters>
      </kineticLaw>
   </multi:intraSpeciesReaction>

<!-- trans association: -->
   <reaction id="rc_Trans_Association" name="Trans_Association" reversible="false" fast="false"
      compartment="inter_membrane">
      <listOfReactants>
         <speciesReference id="Trans_Association_r1" species="sp_Ecad_trans_unbnd"
            multi:compartmentReference="m1" constant="false" />
```

```
            <speciesReference id="Trans_Association_r2" species="sp_Ecad_trans_unbnd"
               multi:compartmentReference="m2" constant="false" />
         </listOfReactants>
         <listOfProducts>
            <speciesReference species="sp_EcadEcad_1" constant="false" />
         </listOfProducts>
         <kineticLaw>
            <math xmlns="http://www.w3.org/1998/Math/MathML">
               <apply>
                  <times />
                  <ci> kon </ci>
                  <ci multi:speciesReference="Trans_Association_r1"> sp_Ecad_trans_unbnd </ci>
                  <ci multi:speciesReference="Trans_Association_r2"> sp_Ecad_trans_unbnd </ci>
               </apply>
            </math>
            <listOfLocalParameters>
               <localParameter id="kon" value="90000" units="litre_per_mole_per_sec" />
            </listOfLocalParameters>
         </kineticLaw>
      </reaction>

<!-- In complex trans association: Here the model requires that the two interacting molecules
        are part of one connected complex already prior to the association. Since the necessary
        connectivity can only be mediated by the cis binding sites here, these sites must be bound
        to the subcomplex (not shown) linking the two interacting molecules.
        -->
      <multi:intraSpeciesReaction id="rc_Intra_Complex_Trans_Association"
       name="Intra-Complex_Trans_Association"
         reversible="false" fast="false" compartment="inter_membrane" >
         <listOfReactants>
            <speciesReference id="Intra_Complex_Trans_Association_r1" species="sp_Ecad_7"
               multi:compartmentReference="m1" constant="false" />
            <speciesReference id="Intra_Complex_Trans_Association_r2" species="sp_Ecad_7"
               multi:compartmentReference="m2" constant="false" />
         </listOfReactants>
         <listOfProducts>
            <speciesReference species="sp_Ecad_trans_dimer_2" constant="false" />
         </listOfProducts>
         <kineticLaw>
            <math xmlns="http://www.w3.org/1998/Math/MathML">
               <apply>
                  <times />
                  <ci> kon </ci>
                  <ci multi:speciesReference="Intra_Complex_Trans_Association_r1"> sp_Ecad_7 </ci>
               </apply>
            </math>
            <listOfLocalParameters>
               <localParameter id="kon" value="100" units="per_sec" />
            </listOfLocalParameters>
         </kineticLaw>
      </multi:intraSpeciesReaction>

<!-- cis dissociation: -->
      <reaction id="rc_Cis_dissociation" name="Cis_dissociation" reversible="false" fast="false"
         compartment="membrane">
         <listOfReactants>
            <speciesReference species="sp_Ecad_cis_dimer" constant="false" />
         </listOfReactants>
         <listOfProducts>
            <speciesReference id="Cis_dissociation_p1" species="sp_Ecad_cis_unbnd"
               stoichiometry="1" constant="false" />
            <speciesReference id="Cis_dissociation_p2" species="sp_Ecad_cis_unbnd"
               stoichiometry="1" constant="false" />
         </listOfProducts>
         <kineticLaw>
            <math xmlns="http://www.w3.org/1998/Math/MathML">
```

```
            <apply>
               <times />
               <ci> koff </ci>
               <ci> sp_Ecad_cis_unbnd </ci>
            </apply>
         </math>
         <listOfLocalParameters>
            <localParameter id="koff" value="1" units="per_sec" />
         </listOfLocalParameters>
      </kineticLaw>
   </reaction>

<!-- In-species cis dissociation: By specifying that this reaction breaks only an inner bond,
     the model limits the application of this reaction to dissociations that result in only one
     reaction product. The complex is still connected through a subcomplex that is not shown
     here but that links the two molecules involved in the reaction at their trans binding
     sites. Note that the modeler application has to ensure the correct application of this
     rule and its consistent definition. For instance, specifying the one or both of the trans
     binding sites to be unbound would lead to a rule that could never be applied because the
     trans bindings are required for the connectivity of the result complex.
     -->
   <multi:intraSpeciesReaction id="rc_Intra_Complex_Cis_dissociation"
    name="Intra-Complex_Cis_dissociation"
      reversible="false" fast="false" compartment="membrane" >
      <listOfReactants>
         <speciesReference species="sp_EcadEcad_2" constant="false" />
      </listOfReactants>
      <listOfProducts>
         <speciesReference id="Intra_Complex_Cis_dissociation_p1" species="sp_Ecad_6"
            stoichiometry="2" constant="false" />
         <speciesReference id="Intra_Complex_Cis_dissociation_p2" species="sp_Ecad_6"
            stoichiometry="2" constant="false" />
      </listOfProducts>
      <kineticLaw>
         <math xmlns="http://www.w3.org/1998/Math/MathML">
            <apply>
               <times />
               <ci> koff </ci>
               <ci> sp_Ecad_6 </ci>
            </apply>
         </math>
         <listOfLocalParameters>
            <localParameter id="koff" value="0.01" units="per_sec" />
         </listOfLocalParameters>
      </kineticLaw>
   </multi:intraSpeciesReaction>

<!-- trans dissociation: -->
   <reaction id="rc_Trans_dissociation" name="Trans_dissociation" reversible="false"
      fast="false" compartment="inter_membrane">
      <listOfReactants>
         <speciesReference species="sp_EcadEcad_1" constant="false" />
      </listOfReactants>
      <listOfProducts>
         <speciesReference id="Trans_dissociation_p1" species="sp_Ecad_trans_unbnd"
            multi:compartmentReference="m1" constant="false" />
         <speciesReference id="Trans_dissociation_p2" species="sp_Ecad_trans_unbnd"
            multi:compartmentReference="m2" constant="false" />
      </listOfProducts>
      <kineticLaw>
         <math xmlns="http://www.w3.org/1998/Math/MathML">
            <apply>
               <times />
               <ci> koff </ci>
               <ci> sp_Ecad_trans_unbnd </ci>
            </apply>
```

```
            </math>
            <listOfLocalParameters>
               <localParameter id="koff" value="1" units="per_sec" />
            </listOfLocalParameters>
         </kineticLaw>
      </reaction>

<!-- In species trans dissociation: By specifying that this reaction breaks only an inner
        bond, the model limits the application of this reaction to dissociations that result in
        only one reaction product. The complex is still connected through a subcomplex that is
        not shown here but that links the two molecules involved in the reaction at their cis
        binding sites. Note that the modeler application has to ensure the correct application
        of this rule and its consistent definition. For instance, specifying the one or both of
        the cis binding sites to be unbound would lead to a rule that could never be applied
        because the cis bindings are required for the connectivity of the result complex.
        -->
      <multi:intraSpeciesReaction id="rc_Intra_Complex_Trans_dissociation"
       name="Intra-Complex_Trans_dissociation"
         reversible="false" fast="false" compartment="inter_membrane" >
         <listOfReactants>
            <speciesReference species="sp_Ecad_trans_dimer_2" constant="false" />
         </listOfReactants>
         <listOfProducts>
            <speciesReference id="Intra_Complex_Trans_dissociation_p1" species="sp_Ecad_7"
               multi:compartmentReference="m1" constant="false" />
            <speciesReference id="Intra_Complex_Trans_dissociation_p2" species="sp_Ecad_7"
               multi:compartmentReference="m2" constant="false" />
         </listOfProducts>
         <kineticLaw>
            <math xmlns="http://www.w3.org/1998/Math/MathML">
               <apply>
                  <times />
                  <ci> koff </ci>
                  <ci> sp_Ecad_7 </ci>
               </apply>
            </math>
            <listOfLocalParameters>
               <localParameter id="koff" value="0.01" units="per_sec" />
            </listOfLocalParameters>
         </kineticLaw>
      </multi:intraSpeciesReaction>
   </listOfReactions>
</model>
</sbml>
```

### 4.3 A *BioNetGen* example from its user manual egfr_simple.bngl (http://bionetgen.org/index.php/BNGManual:Listing_1)

```
begin parameters
  NA 6.02e23                # Avogadro's number (molecules/mol)
  f  1                      # Fraction of the cell to simulate
  Vo f*1.0e-10              # Extracellular volume=1/cell_density (L)
  V  f*3.0e-12              # Cytoplasmic volume (L)

EGF_init 20*1e-9*NA*Vo    # Initial amount of ligand (20 nM)
                            # converted to copies per cell

## Initial amounts of cellular components (copies per cell)
  EGFR_init f*1.8e5
  Grb2_init f*1.5e5
  Sos1_init f*6.2e4

## Rate constants
```

```
  # Divide by NA*V to convert bimolecular rate constants
  # from /M/sec to /(molecule/cell)/sec
  kp1 9.0e7/(NA*Vo)   # ligand-monomer binding
  km1 0.06            # ligand-monomer dissociation
  kp2 1.0e7/(NA*V)    # aggregation of bound monomers
  km2 0.1             # dissociation of bound monomers
  kp3 0.5             # dimer transphosphorylation
  km3 4.505           # dimer dephosphorylation
  kp4 1.5e6/(NA*V)    # binding of Grb2 to receptor
  km4 0.05            # dissociation of Grb2 from receptor
  kp5 1.0e7/(NA*V)    # binding of Grb2 to Sos1
  km5 0.06            # dissociation of Grb2 from Sos1
  deg 0.01            # degradation of receptor dimers
end parameters begin molecule types
  EGF(R)
  EGFR(L,CR1,Y1068~U~P)
  Grb2(SH2,SH3)
  Sos1(PxxP)
  Trash()
end molecule types begin seed species
  EGF(R)              0
  EGFR(L,CR1,Y1068~U) EGFR_init
  Grb2(SH2,SH3)       Grb2_init
  Sos1(PxxP)          Sos1_init
end seed species begin observables
  1 Molecules   EGFR_tot  EGFR()
  2 Molecules   Lig_free  EGF(R)
  3 Species     Dim       EGFR(CR1!+)
  4 Molecules   RP        EGFR(Y1068~P!?)
  5 Molecules   Grb2Sos1  Grb2(SH2,SH3!1).Sos1(PxxP!1)
  6 Molecules   Sos1_act  EGFR(Y1068!1).Grb2(SH2!1,SH3!2).Sos1(PxxP!2)
end observables begin reaction rules
  # Ligand-receptor binding
  1 EGFR(L,CR1) + EGF(R) <-> EGFR(L!1,CR1).EGF(R!1) kp1, km1

## Receptor-aggregation
  2 EGFR(L!+,CR1) + EGFR(L!+,CR1) <-> EGFR(L!+,CR1!1).EGFR(L!+,CR1!1) kp2,km2

## Transphosphorylation of EGFR by RTK
  3 EGFR(CR1!+,Y1068~U) -> EGFR(CR1!+,Y1068~P) kp3

## Dephosphorylation
  4 EGFR(Y1068~P) -> EGFR(Y1068~U) km3

## Grb2 binding to pY1068
  5 EGFR(Y1068~P) + Grb2(SH2) <-> EGFR(Y1068~P!1).Grb2(SH2!1) kp4,km4

## Grb2 binding to Sos1
  6 Grb2(SH3) + Sos1(PxxP) <-> Grb2(SH3!1).Sos1(PxxP!1) kp5,km5

## Receptor dimer internalization/degradation
  7 EGF(R!1).EGF(R!2).EGFR(L!1,CR1!3).EGFR(L!2,CR1!3) -> Trash()
end reaction rules

##actions
generate_network({overwrite=>1});

## Equilibration
```

```
simulate_ode({suffix=>equil,t_end=>100000,n_steps=>10,sparse=>1,steady_state=>1});
setConcentration("EGF(R)","EGF_init");
saveConcentrations(); # Saves concentrations for future reset

## Kinetics
writeSBML({});
simulate_ode({t_end=>120,n_steps=>120});
resetConcentrations(); # reverts to saved Concentrations
simulate_ssa({suffix=>ssa,t_end=>120,n_steps=>120});
```

The SBML code can be as follows. Please note, the SBML code does not cover the content other than the model in the bngl file, such as the "`actions`", "`Equilibration`" and "`Kinetics`" sections.

```
<?xml version="1.0" encoding="UTF-8"?>
<sbml xmlns="http://www.sbml.org/sbml/level3/version1/core" level="3" version="1"
   xmlns:multi="http://www.sbml.org/sbml/level3/version1/multi/version1" multi:required="true">

<!-- Note: this model has been validated agains multi validation rules without errors -->

<model name="bionetgen_example_egfr_simple">

<listOfUnitDefinitions>
         <unitDefinition id="molecules_per_mol">
            <listOfUnits>
               <unit kind="mole" scale="0" multiplier="1" exponent="-1" />
            </listOfUnits>
         </unitDefinition>
      </listOfUnitDefinitions>

<!-- compartments -->
      <listOfCompartments>
         <compartment id="Vo" constant="true" spatialDimensions="3" units="litre"
            multi:isType="false" />
         <compartment id="V" constant="true" spatialDimensions="3" units="litre"
            multi:isType="false" />
      </listOfCompartments>

<!-- speciesType -->
      <multi:listOfSpeciesTypes>

<!-- EGF(R) -->
         <multi:bindingSiteSpeciesType multi:id="st_EGF_bs_R" />
         <multi:speciesType multi:id="st_EGF">
            <multi:listOfSpeciesTypeInstances>
               <multi:speciesTypeInstance multi:id="R" multi:speciesType="st_EGF_bs_R" />
            </multi:listOfSpeciesTypeInstances>
         </multi:speciesType>

<!-- EGFR(L,CR1,Y1068~U~P) -->
         <multi:bindingSiteSpeciesType multi:id="st_EGFR_bs_L" />
         <multi:bindingSiteSpeciesType multi:id="st_EGFR_bs_CR1" />
         <multi:bindingSiteSpeciesType multi:id="st_EGFR_bs_Y1068">
            <multi:listOfSpeciesFeatureTypes>
               <multi:speciesFeatureType multi:id="sft_Y1068" multi:occur="1">
                  <multi:listOfPossibleSpeciesFeatureValues>
                     <multi:possibleSpeciesFeatureValue multi:id="U" />
                     <multi:possibleSpeciesFeatureValue multi:id="P" />
                  </multi:listOfPossibleSpeciesFeatureValues>
               </multi:speciesFeatureType>
            </multi:listOfSpeciesFeatureTypes>
         </multi:bindingSiteSpeciesType>
         <multi:speciesType multi:id="st_EGFR">
            <multi:listOfSpeciesTypeInstances>
               <multi:speciesTypeInstance multi:id="L" multi:speciesType="st_EGFR_bs_L" />
               <multi:speciesTypeInstance multi:id="CR1" multi:speciesType="st_EGFR_bs_CR1" />
```

```
        <multi:speciesTypeInstance multi:id="Y1068" multi:speciesType="st_EGFR_bs_Y1068" />
      </multi:listOfSpeciesTypeInstances>
    </multi:speciesType>

<!-- EGFR dimer: [EGFR(CR1!1).EGFR(CR1!1)] -->
    <multi:speciesType multi:id="st_EGFR_dimer">
      <multi:listOfSpeciesTypeInstances>
        <multi:speciesTypeInstance multi:id="EGFR1" multi:speciesType="st_EGFR" />
        <multi:speciesTypeInstance multi:id="EGFR2" multi:speciesType="st_EGFR" />
      </multi:listOfSpeciesTypeInstances>
      <multi:listOfSpeciesTypeComponentIndexes>
        <multi:speciesTypeComponentIndex multi:id="EGFR1CR1"
          multi:component="CR1" identifyingParent="EGFR1" />
        <multi:speciesTypeComponentIndex multi:id="EGFR2CR1"
          multi:component="CR1" identifyingParent="EGFR2" />
      </multi:listOfSpeciesTypeComponentIndexes>
      <multi:listOfInSpeciesTypeBonds>
        <multi:inSpeciesTypeBond multi:bindingSite1="EGFR1CR1"
          multi:bindingSite2="EGFR2CR1" />
      </multi:listOfInSpeciesTypeBonds>
    </multi:speciesType>

<!-- EGFR-EGF dimer: [EGF(R!1).EGF(R!2).EGFR(L!1,CR1!3).EGFR(L!2,CR1!3)] -->
    <multi:speciesType multi:id="st_EGFR_EGF_dimer">
      <multi:listOfSpeciesTypeInstances>
        <multi:speciesTypeInstance multi:id="EGF1" multi:speciesType="st_EGF" />
        <multi:speciesTypeInstance multi:id="EGF2" multi:speciesType="st_EGF" />
        <multi:speciesTypeInstance multi:id="EGFR1" multi:speciesType="st_EGFR" />
        <multi:speciesTypeInstance multi:id="EGFR2" multi:speciesType="st_EGFR" />
      </multi:listOfSpeciesTypeInstances>
      <multi:listOfSpeciesTypeComponentIndexes>
        <multi:speciesTypeComponentIndex multi:id="EGF1R"
          multi:component="R" identifyingParent="EGF1" />
        <multi:speciesTypeComponentIndex multi:id="EGF2R"
          multi:component="R" identifyingParent="EGF2" />
        <multi:speciesTypeComponentIndex multi:id="EGFR1L"
          multi:component="L" identifyingParent="EGFR1" />
        <multi:speciesTypeComponentIndex multi:id="EGFR2L"
          multi:component="L" identifyingParent="EGFR2" />
        <multi:speciesTypeComponentIndex multi:id="EGFR1CR1"
          multi:component="CR1" identifyingParent="EGFR1" />
        <multi:speciesTypeComponentIndex multi:id="EGFR2CR1"
          multi:component="CR1" identifyingParent="EGFR2" />
      </multi:listOfSpeciesTypeComponentIndexes>
      <multi:listOfInSpeciesTypeBonds>
        <multi:inSpeciesTypeBond multi:bindingSite1="EGFR1CR1" multi:bindingSite2="EGFR2CR1" />
        <multi:inSpeciesTypeBond multi:bindingSite1="EGF1R" multi:bindingSite2="EGFR1L" />
        <multi:inSpeciesTypeBond multi:bindingSite1="EGF2R" multi:bindingSite2="EGFR2L" />
      </multi:listOfInSpeciesTypeBonds>
    </multi:speciesType>

<!-- Grb2(SH2, SH3) -->
    <multi:bindingSiteSpeciesType multi:id="st_Grb2_bs_SH2" />
    <multi:bindingSiteSpeciesType multi:id="st_Grb2_bs_SH3" />
    <multi:speciesType multi:id="st_Grb2">
      <multi:listOfSpeciesTypeInstances>
        <multi:speciesTypeInstance multi:id="SH2" multi:speciesType="st_Grb2_bs_SH2" />
        <multi:speciesTypeInstance multi:id="SH3" multi:speciesType="st_Grb2_bs_SH3" />
      </multi:listOfSpeciesTypeInstances>
    </multi:speciesType>

<!-- Sos1 -->
    <multi:bindingSiteSpeciesType multi:id="st_Sos1_bs_PxxP" />
    <multi:speciesType multi:id="st_Sos1">
      <multi:listOfSpeciesTypeInstances>
        <multi:speciesTypeInstance multi:id="PxxP" multi:speciesType="st_Sos1_bs_PxxP" />
```

```
            </multi:listOfSpeciesTypeInstances>
         </multi:speciesType>

<!-- Trash -->
         <multi:speciesType multi:id="st_trash" />

<!-- Grb2-Sos1 -->
         <multi:speciesType multi:id="st_Grb2_Sos1">
            <multi:listOfSpeciesTypeInstances>
               <multi:speciesTypeInstance multi:id="Grb2" multi:speciesType="st_Grb2" />
               <multi:speciesTypeInstance multi:id="Sos1" multi:speciesType="st_Sos1" />
            </multi:listOfSpeciesTypeInstances>
            <multi:listOfInSpeciesTypeBonds>
               <multi:inSpeciesTypeBond multi:bindingSite1="SH3" multi:bindingSite2="PxxP" />
            </multi:listOfInSpeciesTypeBonds>
         </multi:speciesType>

<!-- EGFR(Y1068!1).Grb1(SH2!1,SH3!2).Sos1(PxxP!2) -->
         <multi:speciesType multi:id="st_EGFR_Grb2_Sos1">
            <multi:listOfSpeciesTypeInstances>
               <multi:speciesTypeInstance multi:id="EGFR" multi:speciesType="st_EGFR" />
               <multi:speciesTypeInstance multi:id="Grb2" multi:speciesType="st_Grb2" />
               <multi:speciesTypeInstance multi:id="Sos1" multi:speciesType="st_Sos1" />
            </multi:listOfSpeciesTypeInstances>
            <multi:listOfInSpeciesTypeBonds>
               <multi:inSpeciesTypeBond multi:bindingSite1="Y1068" multi:bindingSite2="SH2" />
               <multi:inSpeciesTypeBond multi:bindingSite1="SH3" multi:bindingSite2="PxxP" />
            </multi:listOfInSpeciesTypeBonds>
         </multi:speciesType>

<!-- EGFR(L!1).EGF(R!1) -->
         <multi:speciesType multi:id="st_EGFR_EGF">
            <multi:listOfSpeciesTypeInstances>
               <multi:speciesTypeInstance multi:id="EGFR" multi:speciesType="st_EGFR" />
               <multi:speciesTypeInstance multi:id="EGF" multi:speciesType="st_EGF" />
            </multi:listOfSpeciesTypeInstances>
            <multi:listOfInSpeciesTypeBonds>
               <multi:inSpeciesTypeBond multi:bindingSite1="L" multi:bindingSite2="R" />
            </multi:listOfInSpeciesTypeBonds>
         </multi:speciesType>

<!-- EGFR(Y1068!1).Grb2(SH2!1) -->
         <multi:speciesType multi:id="st_EGFR_Grb2">
            <multi:listOfSpeciesTypeInstances>
               <multi:speciesTypeInstance multi:id="EGFR" multi:speciesType="st_EGFR" />
               <multi:speciesTypeInstance multi:id="Grb2" multi:speciesType="st_Grb2" />
            </multi:listOfSpeciesTypeInstances>
            <multi:listOfInSpeciesTypeBonds>
               <multi:inSpeciesTypeBond multi:bindingSite1="Y1068" multi:bindingSite2="SH2" />
            </multi:listOfInSpeciesTypeBonds>
         </multi:speciesType>

</multi:listOfSpeciesTypes>

<!-- species -->
      <listOfSpecies>

<species id="sp_EGF_free" name="EGF(R)" compartment="Vo" multi:speciesType="st_EGF"
            hasOnlySubstanceUnits="false" boundaryCondition="false" constant="false">
            <multi:listOfOutwardBindingSites>
               <multi:outwardBindingSite multi:component="R" multi:bindingStatus="unbound" />
            </multi:listOfOutwardBindingSites>
         </species>

<species id="sp_EGFR_free_U" name="EGFR(L,CR1,Y1068~U)" compartment="V"
            multi:speciesType="st_EGFR" hasOnlySubstanceUnits="false"
```

```
            boundaryCondition="false" constant="false">
            <multi:listOfOutwardBindingSites>
               <multi:outwardBindingSite multi:component="L" multi:bindingStatus="unbound" />
               <multi:outwardBindingSite multi:component="CR1" multi:bindingStatus="unbound" />
               <multi:outwardBindingSite multi:component="Y1068" multi:bindingStatus="unbound" />
            </multi:listOfOutwardBindingSites>
            <multi:listOfSpeciesFeatures>
               <multi:speciesFeature multi:speciesFeatureType="sft_Y1068" multi:occur="1">
                  <multi:listOfSpeciesFeatureValues>
                     <multi:speciesFeatureValue multi:value="U" />
                  </multi:listOfSpeciesFeatureValues>
               </multi:speciesFeature>
            </multi:listOfSpeciesFeatures>
         </species>
         <species id="sp_Grb2_free" name="Grb2(SH2,SH3)" compartment="Vo" multi:speciesType="st_Grb2"
            hasOnlySubstanceUnits="false" boundaryCondition="false" constant="false">
            <multi:listOfOutwardBindingSites>
               <multi:outwardBindingSite multi:component="SH2" multi:bindingStatus="unbound" />
               <multi:outwardBindingSite multi:component="SH3" multi:bindingStatus="unbound" />
            </multi:listOfOutwardBindingSites>
         </species>
         <species id="sp_Grb2_SH2" name="Grb2(SH2)" compartment="V" multi:speciesType="st_Grb2"
            hasOnlySubstanceUnits="false"
            boundaryCondition="false" constant="false">
            <multi:listOfOutwardBindingSites>
               <multi:outwardBindingSite multi:component="SH2" multi:bindingStatus="unbound" />
            </multi:listOfOutwardBindingSites>
         </species>
         <species id="sp_Grb2_SH3" name="Grb2(SH3)" compartment="V" multi:speciesType="st_Grb2"
            hasOnlySubstanceUnits="false"
            boundaryCondition="false" constant="false">
            <multi:listOfOutwardBindingSites>
               <multi:outwardBindingSite multi:component="SH3" multi:bindingStatus="unbound" />
            </multi:listOfOutwardBindingSites>
         </species>
         <species id="sp_Sos1_free" name="Sos1(PxxP)" compartment="V" multi:speciesType="st_Sos1"
            hasOnlySubstanceUnits="false" boundaryCondition="false" constant="false">
            <multi:listOfOutwardBindingSites>
               <multi:outwardBindingSite multi:component="PxxP" multi:bindingStatus="unbound" />
            </multi:listOfOutwardBindingSites>
         </species>
         <species id="sp_EGF_tot" name="EGF()" compartment="Vo" multi:speciesType="st_EGF"
            hasOnlySubstanceUnits="false"
            boundaryCondition="false" constant="false" />
         <species id="sp_EGFR_dimerized" name="EGFR(CR1!+)" compartment="V"
            multi:speciesType="st_EGFR" hasOnlySubstanceUnits="false" boundaryCondition="false"
            constant="false">
            <multi:listOfOutwardBindingSites>
               <multi:outwardBindingSite multi:component="CR1" multi:bindingStatus="bound" />
            </multi:listOfOutwardBindingSites>
         </species>
         <species id="sp_EGFR_U" name="EGFR(Y1068~P!?)" compartment="V"
            multi:speciesType="st_EGFR" hasOnlySubstanceUnits="false" boundaryCondition="false"
            constant="false">
            <multi:listOfSpeciesFeatures>
               <multi:speciesFeature multi:speciesFeatureType="sft_Y1068" multi:occur="1">
                  <multi:listOfSpeciesFeatureValues>
                     <multi:speciesFeatureValue multi:value="P" />
                  </multi:listOfSpeciesFeatureValues>
               </multi:speciesFeature>
            </multi:listOfSpeciesFeatures>
         </species>
         <species id="sp_EGFR_L_CR1" name="EGFR(L,CR1)" compartment="V" multi:speciesType="st_EGFR"
            hasOnlySubstanceUnits="false" boundaryCondition="false" constant="false">
            <multi:listOfOutwardBindingSites>
               <multi:outwardBindingSite multi:component="L" multi:bindingStatus="unbound" />
```

```
               <multi:outwardBindingSite multi:component="CR1" multi:bindingStatus="unbound" />
            </multi:listOfOutwardBindingSites>
         </species>
         <species id="sp_EGFR_EGF_CR1" name="EGFR(L!1,CR1).EGF(R!1)" compartment="V"
            multi:speciesType="st_EGFR_EGF" hasOnlySubstanceUnits="false" boundaryCondition="false"
            constant="false">
            <multi:listOfOutwardBindingSites>
               <multi:outwardBindingSite multi:component="CR1" multi:bindingStatus="unbound" />
            </multi:listOfOutwardBindingSites>
         </species>
         <species id="sp_EGFR_bL_CR1" name="EGFR(L!+,CR1)" compartment="V"
            multi:speciesType="st_EGFR" hasOnlySubstanceUnits="false" boundaryCondition="false"
            constant="false">
            <multi:listOfOutwardBindingSites>
               <multi:outwardBindingSite multi:component="L" multi:bindingStatus="bound" />
               <multi:outwardBindingSite multi:component="CR1" multi:bindingStatus="unbound" />
            </multi:listOfOutwardBindingSites>
         </species>
         <species id="sp_EGFR_dimer_bL" name="EGFR(L!+,CR1!1).EGFR(L!+,CR1!1)" compartment="V"
            multi:speciesType="st_EGFR_dimer"
            hasOnlySubstanceUnits="false" boundaryCondition="false" constant="false">
            <multi:listOfOutwardBindingSites>
               <multi:outwardBindingSite multi:component="EGFR1L"
                  multi:bindingStatus="bound" />
               <multi:outwardBindingSite multi:component="EGFR2L"
                  multi:bindingStatus="bound" />
            </multi:listOfOutwardBindingSites>
         </species>
         <species id="sp_EGFR_EGF_dimer" name="EGF(R!1).EGF(R!2).EGFR(L!1,CR1!3).EGFR(L!2,CR1!3)"
            compartment="V" multi:speciesType="st_EGFR_EGF_dimer" hasOnlySubstanceUnits="false"
            boundaryCondition="false" constant="false" />
         <species id="sp_EGFR_bCR1_Y1068_U" name="EGFR(CR1!+,Y1068~U)" compartment="V"
            multi:speciesType="st_EGFR" hasOnlySubstanceUnits="false" boundaryCondition="false"
            constant="false">
            <multi:listOfOutwardBindingSites>
               <multi:outwardBindingSite multi:component="CR1" multi:bindingStatus="bound" />
               <multi:outwardBindingSite multi:component="Y1068" multi:bindingStatus="unbound" />
            </multi:listOfOutwardBindingSites>
            <multi:listOfSpeciesFeatures>
               <multi:speciesFeature multi:speciesFeatureType="sft_Y1068" multi:occur="1">
                  <multi:listOfSpeciesFeatureValues>
                     <multi:speciesFeatureValue multi:value="U" />
                  </multi:listOfSpeciesFeatureValues>
               </multi:speciesFeature>
            </multi:listOfSpeciesFeatures>
         </species>
         <species id="sp_EGFR_bCR1_Y1068_P" name="EGFR(CR1!+,Y1068~P)" multi:speciesType="st_EGFR"
            compartment="V" hasOnlySubstanceUnits="false" boundaryCondition="false" constant="false">
            <multi:listOfOutwardBindingSites>
               <multi:outwardBindingSite multi:component="CR1" multi:bindingStatus="bound" />
               <multi:outwardBindingSite multi:component="Y1068" multi:bindingStatus="unbound" />
            </multi:listOfOutwardBindingSites>
            <multi:listOfSpeciesFeatures>
               <multi:speciesFeature multi:speciesFeatureType="sft_Y1068" multi:occur="1">
                  <multi:listOfSpeciesFeatureValues>
                     <multi:speciesFeatureValue multi:value="P" />
                  </multi:listOfSpeciesFeatureValues>
               </multi:speciesFeature>
            </multi:listOfSpeciesFeatures>
         </species>
         <species id="sp_EGFR_Y1068_P" name="EGFR(Y1068~P)" multi:speciesType="st_EGFR"
            compartment="V" hasOnlySubstanceUnits="false" boundaryCondition="false" constant="false">
            <multi:listOfOutwardBindingSites>
               <multi:outwardBindingSite multi:component="Y1068" multi:bindingStatus="unbound" />
            </multi:listOfOutwardBindingSites>
            <multi:listOfSpeciesFeatures>
```

```
               <multi:speciesFeature multi:speciesFeatureType="sft_Y1068" multi:occur="1">
                  <multi:listOfSpeciesFeatureValues>
                     <multi:speciesFeatureValue multi:value="P" />
                  </multi:listOfSpeciesFeatureValues>
               </multi:speciesFeature>
            </multi:listOfSpeciesFeatures>
         </species>
         <species id="sp_EGFR_Y1068_U" name="EGFR(Y1068~U)" multi:speciesType="st_EGFR"
            compartment="V" hasOnlySubstanceUnits="false" boundaryCondition="false" constant="false">
            <multi:listOfOutwardBindingSites>
               <multi:outwardBindingSite multi:component="Y1068" multi:bindingStatus="unbound" />
            </multi:listOfOutwardBindingSites>
            <multi:listOfSpeciesFeatures>
               <multi:speciesFeature multi:speciesFeatureType="sft_Y1068" multi:occur="1">
                  <multi:listOfSpeciesFeatureValues>
                     <multi:speciesFeatureValue multi:value="U" />
                  </multi:listOfSpeciesFeatureValues>
               </multi:speciesFeature>
            </multi:listOfSpeciesFeatures>
         </species>
         <species id="sp_EGFR_Grb2_P" name="EGFR(Y1068~P!1).Grb2(SH2!1)"  compartment="V"
            multi:speciesType="st_EGFR_Grb2"
            hasOnlySubstanceUnits="false" boundaryCondition="false" constant="false">
            <multi:listOfSpeciesFeatures>
               <multi:speciesFeature multi:speciesFeatureType="sft_Y1068" multi:occur="1">
                  <multi:listOfSpeciesFeatureValues>
                     <multi:speciesFeatureValue multi:value="P" />
                  </multi:listOfSpeciesFeatureValues>
               </multi:speciesFeature>
            </multi:listOfSpeciesFeatures>
         </species>
         <species id="sp_Grb2_Sos1" name="Grb2(SH3!1).Sos1(PxxP!1)" compartment="V"
            multi:speciesType="st_Grb2_Sos1" hasOnlySubstanceUnits="false"
            boundaryCondition="false" constant="false" />

<species id="sp_Trash" name="Trash()" compartment="V" multi:speciesType="st_trash"
            hasOnlySubstanceUnits="false"
            boundaryCondition="false" constant="false" />
      </listOfSpecies>

<!-- parameters -->
      <listOfParameters>
         <parameter id="NA" value="6.02e23" constant="true" units="molecules_per_mol" />
         <parameter id="f" value="1" constant="true" />
         <parameter id="kp1" constant="true" />
         <parameter id="km1" value="0.06" constant="true" />
         <parameter id="kp2" constant="true" />
         <parameter id="km2" value="0.1" constant="true" />
         <parameter id="kp3" value="0.5" constant="true" />
         <parameter id="km3" value="4.505" constant="true" />
         <parameter id="kp4" constant="true" />
         <parameter id="km4" value="0.05" constant="true" />
         <parameter id="kp5" constant="true" />
         <parameter id="km5" value="0.06" constant="true" />
         <parameter id="deg" value="0.01" constant="true" />
      </listOfParameters>

<!-- intiialAssignments -->
      <listOfInitialAssignments>

<initialAssignment symbol="Vo">
            <math xmlns="http://www.w3.org/1998/Math/MathML">
               <apply>
                  <times />
                  <ci>f</ci>
                  <cn> 1e-10 </cn>
```

```
            </apply>
         </math>
      </initialAssignment>
      <initialAssignment symbol="V">
         <math xmlns="http://www.w3.org/1998/Math/MathML">
            <apply>
               <times />
               <ci>f</ci>
               <cn> 3e-12 </cn>
            </apply>
         </math>
      </initialAssignment>
      <initialAssignment symbol="kp1">
         <math xmlns="http://www.w3.org/1998/Math/MathML">
            <apply>
               <divide />
               <cn>9.02e7</cn>
               <apply>
                  <times />
                  <ci>NA</ci>
                  <ci>Vo</ci>
               </apply>
            </apply>
         </math>
      </initialAssignment>
      <initialAssignment symbol="kp2">
         <math xmlns="http://www.w3.org/1998/Math/MathML">
            <apply>
               <divide />
               <cn>1.0e7</cn>
               <apply>
                  <times />
                  <ci>NA</ci>
                  <ci>V</ci>
               </apply>
            </apply>
         </math>
      </initialAssignment>
      <initialAssignment symbol="kp4">
         <math xmlns="http://www.w3.org/1998/Math/MathML">
            <apply>
               <divide />
               <cn>1.5e6</cn>
               <apply>
                  <times />
                  <ci>NA</ci>
                  <ci>V</ci>
               </apply>
            </apply>
         </math>
      </initialAssignment>
      <initialAssignment symbol="kp5">
         <math xmlns="http://www.w3.org/1998/Math/MathML">
            <apply>
               <divide />
               <cn>1.0e7</cn>
               <apply>
                  <times />
                  <ci>NA</ci>
                  <ci>V</ci>
               </apply>
            </apply>
         </math>
      </initialAssignment>
      <initialAssignment symbol="sp_EGF_free">
         <math xmlns="http://www.w3.org/1998/Math/MathML">
```

```
               <apply>
                  <times />
                  <cn>20</cn>
                  <cn>1e-9</cn>
                  <ci>NA</ci>
                  <ci>Vo</ci>
               </apply>
            </math>
         </initialAssignment>
         <initialAssignment symbol="sp_EGFR_free_U">
            <math xmlns="http://www.w3.org/1998/Math/MathML">
               <apply>
                  <times />
                  <ci>f</ci>
                  <cn>1.8e5</cn>
               </apply>
            </math>
         </initialAssignment>
         <initialAssignment symbol="sp_Grb2_free">
            <math xmlns="http://www.w3.org/1998/Math/MathML">
               <apply>
                  <times />
                  <ci>f</ci>
                  <cn>1.5e5</cn>
               </apply>
            </math>
         </initialAssignment>
         <initialAssignment symbol="sp_Sos1_free">
            <math xmlns="http://www.w3.org/1998/Math/MathML">
               <apply>
                  <times />
                  <ci>f</ci>
                  <cn>6.2e4</cn>
               </apply>
            </math>
         </initialAssignment>
      </listOfInitialAssignments>

<!-- reactions -->
      <listOfReactions>

<!-- # Ligand-receptor binding -->
         <!-- 1 EGFR(L,CR1) + EGF(R) <-> EGFR(L!1,CR1).EGF(R!1) kp1, km1 -->
         <reaction id="rc_Ligand_receptor_binding" reversible="true" fast="false">
            <listOfReactants>
               <speciesReference species="sp_EGFR_L_CR1" constant="false" />
               <speciesReference species="sp_EGF_free" constant="false" />
            </listOfReactants>
            <listOfProducts>
               <speciesReference species="sp_EGFR_EGF_CR1" constant="false" />
            </listOfProducts>
            <kineticLaw>
               <math xmlns="http://www.w3.org/1998/Math/MathML">
                  <apply>
                     <minus />
                     <apply>
                        <times />
                        <ci> kp1 </ci>
                        <ci> sp_EGFR_L_CR1 </ci>
                        <ci> sp_EGF_free </ci>
                     </apply>
                     <apply>
                        <times />
                        <ci> km1 </ci>
                        <ci> sp_EGFR_EGF_CR1 </ci>
                     </apply>
```

```
            </apply>
         </math>
      </kineticLaw>
   </reaction>

<!-- # Receptor-aggregation -->
   <!-- 2 EGFR(L!+,CR1) + EGFR(L!+,CR1) <-> EGFR(L!+,CR1!1).EGFR(L!+,CR1!1) kp2,km2 -->
   <reaction id="rc_Receptor_aggregation" reversible="true" fast="false">
      <listOfReactants>
         <speciesReference species="sp_EGFR_bL_CR1" constant="false" stoichiometry="2" />
      </listOfReactants>
      <listOfProducts>
         <speciesReference species="sp_EGFR_dimer_bL" constant="false" />
      </listOfProducts>
      <kineticLaw>
         <math xmlns="http://www.w3.org/1998/Math/MathML">
            <apply>
               <minus />
               <apply>
                  <times />
                  <ci> kp2 </ci>
                  <ci> sp_EGFR_bL_CR1 </ci>
                  <ci> sp_EGFR_bL_CR1 </ci>
               </apply>
               <apply>
                  <times />
                  <ci> km3 </ci>
                  <ci> sp_EGFR_dimer_bL </ci>
               </apply>
            </apply>
         </math>
      </kineticLaw>
   </reaction>

<!-- # Transphosphorylation of EGFR by RTK -->
   <!-- 3 EGFR(CR1!+,Y1068~U) -> EGFR(CR1!+,Y1068~P) kp3 -->
   <reaction id="rc_Transphosphorylation" reversible="false" fast="false">
      <listOfReactants>
         <speciesReference species="sp_EGFR_bCR1_Y1068_U" constant="false" />
      </listOfReactants>
      <listOfProducts>
         <speciesReference species="sp_EGFR_bCR1_Y1068_P" constant="false" />
      </listOfProducts>
      <kineticLaw>
         <math xmlns="http://www.w3.org/1998/Math/MathML">
            <apply>
               <times />
               <ci>kp3</ci>
               <ci>sp_EGFR_bCR1_Y1068_U</ci>
            </apply>
         </math>
      </kineticLaw>
   </reaction>

<!-- # Dephosphorylation -->
   <!-- 4 EGFR(Y1068~P) -> EGFR(Y1068~U) km3 -->
   <reaction id="rc_Dephosphorylation" reversible="false" fast="false">
      <listOfReactants>
         <speciesReference species="sp_EGFR_Y1068_P" constant="false" />
      </listOfReactants>
      <listOfProducts>
         <speciesReference species="sp_EGFR_Y1068_U" constant="false" />
      </listOfProducts>
      <kineticLaw>
         <math xmlns="http://www.w3.org/1998/Math/MathML">
            <apply>
```

```
            <times />
            <ci>km3</ci>
            <ci>sp_EGFR_Y1068_P</ci>
         </apply>
      </math>
   </kineticLaw>
</reaction>

<!-- # Grb2 binding to pY1068 -->
<!-- 5 EGFR(Y1068~P) + Grb2(SH2) <-> EGFR(Y1068~P!1).Grb2(SH2!1) kp4,km4 -->
<reaction id="rc_Grb2_binding_to_pY1068" reversible="true" fast="false">
   <listOfReactants>
      <speciesReference species="sp_EGFR_Y1068_P" constant="false" />
      <speciesReference species="sp_Grb2_SH2" constant="false" />
   </listOfReactants>
   <listOfProducts>
      <speciesReference species="sp_EGFR_Grb2_P" constant="false" />
   </listOfProducts>
   <kineticLaw>
      <math xmlns="http://www.w3.org/1998/Math/MathML">
         <apply>
            <minus />
            <apply>
               <times />
               <ci> kp4 </ci>
               <ci> sp_EGFR_Y1068_P </ci>
               <ci> sp_Grb2_SH2 </ci>
            </apply>
            <apply>
               <times />
               <ci> km4 </ci>
               <ci> sp_EGFR_Grb2_P </ci>
            </apply>
         </apply>
      </math>
   </kineticLaw>
</reaction>

<!-- # Grb2 binding to Sos1 -->
<!-- 6 Grb2(SH3) + Sos1(PxxP) <-> Grb2(SH3!1).Sos1(PxxP!1) kp5,km5 -->
<reaction id="rc_Grb2_binding_to_Sos1" reversible="true" fast="false">
   <listOfReactants>
      <speciesReference species="sp_Grb2_SH3" constant="false" />
      <speciesReference species="sp_Sos1_free" constant="false" />
   </listOfReactants>
   <listOfProducts>
      <speciesReference species="sp_Grb2_Sos1" constant="false" />
   </listOfProducts>
   <kineticLaw>
      <math xmlns="http://www.w3.org/1998/Math/MathML">
         <apply>
            <minus />
            <apply>
               <times />
               <ci> kp5 </ci>
               <ci> sp_Grb2_SH3 </ci>
               <ci> sp_Sos1_free </ci>
            </apply>
            <apply>
               <times />
               <ci> km5 </ci>
               <ci> sp_Grb2_Sos1 </ci>
            </apply>
         </apply>
      </math>
   </kineticLaw>
```

```
        </reaction>

<!-- # Receptor dimer internalization/degradation -->
        <!-- 7 EGF(R!1).EGF(R!2).EGFR(L!1,CR1!3).EGFR(L!2,CR1!3) -> Trash() -->
        <reaction id="rc_EGFR_EGF_dimer_degration" reversible="false" fast="false">
           <listOfReactants>
              <speciesReference species="sp_EGFR_EGF_dimer" constant="false" />
           </listOfReactants>
           <listOfProducts>
              <speciesReference species="sp_Trash" constant="false" />
           </listOfProducts>
           <kineticLaw>
              <math xmlns="http://www.w3.org/1998/Math/MathML">
                 <apply>
                    <times />
                    <ci>deg</ci>
                    <ci>sp_EGFR_EGF_dimer</ci>
                 </apply>
              </math>
           </kineticLaw>
        </reaction>
     </listOfReactions>
  </model>
</sbml>
```

### 4.4 Example from *Kappa*'s documentation

Here is the example “An Introduction to Kappa Syntax” at *Kappa* website (http://www.kappalanguage.org/syntax.html).

Rule in English: “Unphosphorylated Site1 of A binds to Site1 of B”

Kappa Rule: `A(Site1 u),B(Site1) -> A(Site1 u!1),B(Site1!1)`

```
<?xml version="1.0" encoding="UTF-8"?>
<sbml xmlns="http://www.sbml.org/sbml/level3/version1/core" level="3" version="1"
   xmlns:multi="http://www.sbml.org/sbml/level3/version1/multi/version1" multi:required="true">

<!-- Note: this example is for the purpose of demonstration; it is not a complete model-->

<model name="An␣Introduction␣to␣Kappa␣Syntax">
      ...
      <!-- speciesType -->
      <multi:listOfSpeciesTypes>

<!-- A:Site1 -->
         <multi:bindingSiteSpeciesType multi:id="st_A_Site1">
            <multi:listOfSpeciesFeatureTypes>
               <multi:speciesFeatureType multi:id="phosphorylation" multi:occur="1">
                  <multi:listOfPossibleSpeciesFeatureValues>
                     <multi:possibleSpeciesFeatureValue multi:id="U" />
                     <multi:possibleSpeciesFeatureValue multi:id="P" />
                  </multi:listOfPossibleSpeciesFeatureValues>
               </multi:speciesFeatureType>
            </multi:listOfSpeciesFeatureTypes>
         </multi:bindingSiteSpeciesType>

<!-- A -->
         <multi:speciesType multi:id="st_A">
            <multi:listOfSpeciesTypeInstances>
               <multi:speciesTypeInstance multi:id="Asite1" multi:speciesType="st_A_Site1" />
            </multi:listOfSpeciesTypeInstances>
         </multi:speciesType>

```

```
        <!-- B:Site1 -->
        <multi:bindingSiteSpeciesType multi:id="st_B_Site1" />

<!-- B -->
        <multi:speciesType multi:id="st_B">
           <multi:listOfSpeciesTypeInstances>
              <multi:speciesTypeInstance multi:id="Bsite1" multi:speciesType="st_B_Site1" />
           </multi:listOfSpeciesTypeInstances>
        </multi:speciesType>

<!-- A.B -->
        <multi:speciesType multi:id="st_AB">
           <multi:listOfSpeciesTypeInstances>
              <multi:speciesTypeInstance multi:id="A" multi:speciesType="st_A" />
              <multi:speciesTypeInstance multi:id="B" multi:speciesType="st_B" />
           </multi:listOfSpeciesTypeInstances>
           <multi:listOfInSpeciesTypeBonds>
              <multi:inSpeciesTypeBond multi:bindingSite1="Asite1"
                 multi:bindingSite2="Bsite1" />
           </multi:listOfInSpeciesTypeBonds>
        </multi:speciesType>
        ...
     </multi:listOfSpeciesTypes>

<!-- species -->
     <listOfSpecies>
        <!-- species A with free unphosphorylated Site1 -->
        <species id="sp_A" name="A␣with␣Unphosphorylated␣Site␣1" multi:speciesType="st_A"
           hasOnlySubstanceUnits="false" boundaryCondition="false" constant="false">
           <multi:listOfOutwardBindingSites>
              <multi:outwardBindingSite multi:component="Asite1"
                 multi:bindingStatus="unbound" />
           </multi:listOfOutwardBindingSites>
           <multi:listOfSpeciesFeatures>
              <multi:speciesFeature multi:speciesFeatureType="phosphorylation">
                 <multi:listOfSpeciesFeatureValues>
                    <multi:speciesFeatureValue multi:value="U" />
                 </multi:listOfSpeciesFeatureValues>
              </multi:speciesFeature>
           </multi:listOfSpeciesFeatures>
        </species>

<!-- species B with free Site 1 -->
        <species id="sp_B" name="B" multi:speciesType="st_B" hasOnlySubstanceUnits="false"
           boundaryCondition="false" constant="false">
           <multi:listOfOutwardBindingSites>
              <multi:outwardBindingSite multi:component="sti_B_Site1"
                 multi:bindingStatus="unbound" />
           </multi:listOfOutwardBindingSites>
        </species>

<!-- species AB: unphosphorylated -->
        <species id="sp_AB" name="AB" multi:speciesType="st_AB" hasOnlySubstanceUnits="false"
           boundaryCondition="false" constant="false">
           <multi:listOfSpeciesFeatures>
              <multi:speciesFeature multi:speciesFeatureType="phosphorylation">
                 <multi:listOfSpeciesFeatureValues>
                    <multi:speciesFeatureValue multi:value="U" />
                 </multi:listOfSpeciesFeatureValues>
              </multi:speciesFeature>
           </multi:listOfSpeciesFeatures>
        </species>
        ...
     </listOfSpecies>

<!-- reactions -->
```

```
    <listOfReactions>

<!-- Unphosphorylated Site1 of A binds to Site1 of B -->
      <!-- Kappa Rule: A(Site1~u),B(Site1) -> A(Site1~u!1),B(Site1!1) -->
      <reaction id="rc_AB" reversible="false" fast="false">
        <listOfReactants>
          <speciesReference species="sp_A" constant="false" />
          <speciesReference species="sp_B" constant="false" />
        </listOfReactants>
        <listOfProducts>
          <speciesReference species="sp_AB" constant="false" />
        </listOfProducts>
        <kineticLaw>
          ...
        </kineticLaw>
      </reaction>
      ...
    </listOfReactions>
  </model>
</sbml>
```

## A Validation of SBML documents using Multi constructs

This section summarizes all the conditions that should be true of an SBML Level 3 Version 1 model that uses the Multi package. We use the same conventions that are used in the SBML Level 3 Version 1 Core specification document. In particular, there are different degrees of rule strictness. Formally, the differences are expressed in the statement of a rule: either a rule states that a condition *must* be true, or a rule states that it *should* be true. Rules of the former kind are strict SBML validation rules—a model encoded in SBML must conform to all of them in order to be considered valid. Rules of the latter kind are consistency rules. To help highlight these differences, we use the three symbols next to the rule numbers as described in section A of the SBML Level 3 Version 1 Core specification document:

- ☑ A checked box indicates a *requirement* for SBML conformance. If a model does not follow this rule, it does not conform to the Multi package specification. (Mnemonic intention behind the choice of symbol: "This must be checked.")
- ▲ A triangle indicates a *recommendation* for model consistency. If a model does not follow this rule, it is not considered strictly invalid as far as the Multi package specification is concerned; however, it indicates that the model contains a physical or conceptual inconsistency. (Mnemonic intention behind the choice of symbol: "This is a cause for warning.")
- ★ A star indicates a strong recommendation for good modeling practice. This rule is not strictly a matter of SBML encoding, but the recommendation comes from logical reasoning. As in the previous case, if a model does not follow this rule, it is not considered an invalid SBML encoding. (Mnemonic intention behind the choice of symbol: "You're a star if you heed this.")

The validation rules listed in the following subsections are all stated or implied in the rest of this specification document. They are enumerated here for convenience. Unless explicitly stated, all validation rules concern objects and attributes specifically defined in the Multi package.

For convenience and brevity, we use the shorthand "`multi:x`" to stand for an attribute or element name `x` in the namespace for the Multi package, using the namespace prefix `multi`. We use "`multi:x`" because it is shorter than to write a full explanation everywhere we refer to an attribute or element in the Multi package namespace.

### General rules about the Multi package

**multi-10101** ☑ To conform to Version 1 of the Multi package specification for SBML Level 3, an SBML document must declare the use of the following XML Namespace: "`http://www.sbml.org/sbml/level3/version1/multi/version1`". (References: SBML Level 3 Package Specification for Multi Version 1, Section 3.1 on page 9.)

**multi-10102** ☑ Wherever they appear in an SBML document, elements and attributes from the Multi package must be declared either implicitly or explicitly to be in the XML namespace "`http://www.sbml.org/sbml/level3/version1/multi/version1`". (References: SBML Level 3 Package Specification for Multi Version 1, Section 3.1 on page 9.)

### General rules about MathML content in the Multi package

**multi-10201** ☑ A `ci` element in a **Math** object may have the optional attributes `multi:speciesReference` and `multi:representationType`. No other attributes from the Multi namespace are permitted on a `ci` element. (References: Section 3.26 on page 33).

**multi-10202** ☑ The value of the `multi:speciesReference` attribute on a given `ci` element must be the identifier of a **SpeciesReference** object within the same reaction. (References: Section 3.26.1 on page 33.)

**multi-10203** ☑ The value of the `multi:representationType` attribute on a given `ci` element must conform to the syntax of the Multi data type **RepresentationType**. (References: Section 3.26.2 on page 34.)

### General rules about identifiers

**multi-10301** ☑ (Extends validation rule #10301 in the SBML Level 3 Version 1 Core specification.) Within a **Model** object, the values of the attributes `id` and `multi:id` on every instance of the following classes of objects must be unique across the set of all `id` and `multi:id` attribute values of all such objects in a model: the **Model** itself, plus all contained **FunctionDefinition**, **Compartment**, **Species**, **Reaction**, **SpeciesReference**, **ModifierSpeciesReference**, **Event**, and **Parameter** objects, plus the **SpeciesType** and **PossibleSpeciesFeatureValue** objects defined by the Multi package, and any objects defined by any other package with `package:id` attributes defined as falling in the 'SId' namespace. (References: Section 3.27 on page 35.)

**multi-10302** ☑ The value of a `multi:id` attribute must always conform to the syntax of the SBML data type `SId`. (References: SBML Level 3 Version 1 Core, Section 3.1.7.)

**multi-10303** ☑ The value of a `multi:name` attribute must always conform to the syntax of type `string`. (References: SBML Level 3 Version 1 Core, Section 3.1.1.)

**multi-10304** ☑ The value of a `multi:id` attribute on **SpeciesTypeInstance** objects must be unique across the set of all `multi:id` attribute values of all the **SpeciesTypeInstance** objects under the direct parent **SpeciesType** object in which it is located. (References: Section 3.11.1 on page 16 and Section 3.27 on page 35.)

**multi-10305** ☑ The value of a `multi:id` attribute on **SpeciesTypeComponentIndex** objects must be unique across the set of all `multi:id` attribute values of all the **SpeciesTypeComponentIndex** objects under the direct parent **SpeciesType** object in which it is located. (References: Section 3.12.1 on page 17 and Section 3.27 on page 35.)

**multi-10306** ☑ The value of a `multi:id` attribute on **InSpeciesTypeBond** objects must be unique across the set of all `multi:id` attribute values of all the **InSpeciesTypeBond** objects under the direct parent **SpeciesType** object in which it is located. (References: Section 3.13.1 on page 19 and Section 3.27 on page 35.)

**multi-10307** ☑ The value of a `multi:id` attribute on **SpeciesFeatureType** objects must be unique across the set of all `multi:id` attribute values of all the **SpeciesFeatureType** objects under the direct parent **SpeciesType** object in which it is located. (References: Section 3.9.1 on page 14 and Section 3.27 on page 35.)

**multi-10308** ☑ The value of a `multi:id` attribute on **SubListOfSpeciesFeatures** objects must be unique across the set of all `id` and `multi:id` attribute values of all objects in the **Species** object in which it is located. (References: Section 3.17.1 on page 25 and Section 3.27 on page 35.)

**multi-10309** ☑ The value of a `multi:id` attribute on **SpeciesFeature** objects must be unique across the set of all `id` and `multi:id` attribute values of all objects in the **Species** object in which it is located. (References: Section 3.18.1 on page 26 and Section 3.27 on page 35.)

**multi-10310** ☑ The value of a `multi:id` attribute on **CompartmentReference** objects must be unique across the set of all `id` and `multi:id` attribute values of all objects in the **Compartment** object in which it is located. (References: Section 3.6.1 on page 12 and Section 3.27 on page 35.)

**multi-10311** ☑ The value of a `multi:compartment` attribute on **SpeciesType** objects must conform to the syntax of the SBML data type `SIdRef`. (References: Section 3.8.2 on page 13.)

**multi-10312** ☑ The value of a multi:numericValue attribute on **PossibleSpeciesFeatureValue** objects must conform to the syntax of the SBML data type SIdRef. (References: Section 3.10.2 on page 15.)

**multi-10313** ☑ The value of a multi:speciesType attribute on **SpeciesTypeInstance** objects must conform to the syntax of the SBML data type SIdRef. (References: Section 3.11.2 on page 16.)

**multi-10314** ☑ The value of a multi:compartmentReference attribute on **SpeciesTypeInstance** objects must conform to the syntax of the SBML data type SIdRef. (References: Section 3.11.3 on page 16.)

**multi-10315** ☑ The value of a multi:component attribute on **SpeciesTypeComponentIndex** objects must conform to the syntax of the SBML data type SIdRef. (References: Section 3.12.2 on page 17.)

**multi-10316** ☑ The value of a multi:identifyingParent attribute on **SpeciesTypeComponentIndex** objects must conform to the syntax of the SBML data type SIdRef. (References: Section 3.12.3 on page 18.)

**multi-10317** ☑ The value of a multi:bindingSite1 attribute on **InSpeciesTypeBond** objects must conform to the syntax of the SBML data type SIdRef. (References: Section 3.13.2 on page 20.)

**multi-10318** ☑ The value of a multi:bindingSite2 attribute on **InSpeciesTypeBond** objects must conform to the syntax of the SBML data type SIdRef. (References: Section 3.13.2 on page 20.)

**multi-10319** ☑ The value of a multi:speciesType attribute on **Species** objects must conform to the syntax of the SBML data type SIdRef. (References: Section 3.15.1 on page 22.)

**multi-10320** ☑ The value of a multi:component attribute on **OutwardBindingSite** objects must conform to the syntax of the SBML data type SIdRef. (References: Section 3.16.3 on page 23.)

**multi-10321** ☑ The value of a multi:speciesFeatureType attribute on **SpeciesFeature** objects must conform to the syntax of the SBML data type SIdRef. (References: Section 3.18.2 on page 26.)

**multi-10322** ☑ The value of a multi:component attribute on **SpeciesFeature** objects must conform to the syntax of the SBML data type SIdRef. (References: Section 3.18.4 on page 27.)

**multi-10323** ☑ The value of a multi:value attribute on **SpeciesFeatureValue** objects must conform to the syntax of the SBML data type SIdRef. (References: Section 3.18.6 on page 27.)

**multi-10324** ☑ The value of a multi:compartmentReference attribute on **SimpleSpeciesReference** objects must conform to the syntax of the SBML data type SIdRef. (References: Section 3.22 on page 30.)

**multi-10325** ☑ The value of a multi:reactant attribute on **SpeciesTypeComponentMapInProduct** objects must conform to the syntax of the SBML data type SIdRef. (References: Section 3.24.2 on page 33.)

**multi-10326** ☑ The value of a multi:reactantComponent attribute on **SpeciesTypeComponentMapInProduct** objects must conform to the syntax of the SBML data type SIdRef. (References: Section 3.24.3 on page 33.)

**multi-10327** ☑ The value of a multi:productComponent attribute on **SpeciesTypeComponentMapInProduct** objects must conform to the syntax of the SBML data type SIdRef. (References: Section 3.24.4 on page 33.)

**multi-10328** ☑ The value of a multi:compartmentType attribute on **Compartment** objects must conform to the syntax of the SBML data type SIdRef. (References: Section 3.5.2 on page 11.)

**multi-10329** ☑ The value of a multi:compartment attribute on **CompartmentReference** objects must conform to the syntax of the SBML data type SIdRef. (References: Section 3.6.2 on page 12.)

### Rules for extended SBML object

**multi-20101** ☑ The `multi:required` attribute is required on the `<sbml>` element in the Multi package. (References: SBML Level 3 Package Specification for Multi Version 1, Section 3.1 on page 9.)

**multi-20102** ☑ The `multi:required` attribute on the `<sbml>` element must be `Boolean`. (References: SBML Level 3 Package Specification for Multi Version 1, Section 3.1 on page 9.)

**multi-20103** ☑ The value of the `multi:required` attribute on the `<sbml>` element must be "`true`". (References: SBML Level 3 Package Specification for Multi Version 1, Section 3.1 on page 9.)

### Rules for extended Model objects

**multi-20201** ☑ There may be at most one **ListOfSpeciesTypes** container object within a **Model** object. (References: Section 3.3 on page 10.)

**multi-20202** ☑ A **ListOfSpeciesTypes** object within an extended **Model** object is optional, but if present, must not be empty. (References: Section 3.3 on page 10.)

**multi-20203** ☑ A **ListOfSpeciesTypes** object may have the optional SBML core attributes `metaid` and `sboTerm`. No other attributes from the SBML Level 3 Core namespace or the Multi namespace are permitted on a **ListOfSpeciesTypes** object. (References: Section 3.4.1 on page 11.)

**multi-20204** ☑ Apart from the general `notes` and `annotation` subobjects permitted on all SBML objects, a **ListOfSpeciesTypes** container object may only contain **SpeciesType** objects. (References: Section 3.4.1 on page 11.)

### Rules for extended Compartment objects

**multi-20301** ☑ An extended **Compartment** object must have the required attribute `multi:isType`, and may also have the optional attribute `multi:comparetmentType`. No other attributes from the Multi namespace are permitted on an extended **Compartment** object.(References: Section 3.5 on page 11.)

**multi-20302** ☑ The value of a `multi:isType` attribute on an extended **Compartment** object must always confirm to the syntax of the SBML data type `boolean`. (References: Section 3.5.1 on page 11.)

**multi-20303** ☑ The `multi:isType` attribute on an extended **Compartment** object is required. (References: Section 3.5.1 on page 11.)

**multi-20304** ☑ The value of the `multi:isType` attribute of the **Compartment** object referenced by a **CompartmentReference** object must be the same as that of the `multi:isType` attribute of the parent **Compartment** object of the **ListOfCompartmentReferences** object which contains the **CompartmentReference** object. (References: Section 3.7 on page 12.)

**multi-20305** ☑ The `multi:compartmentType` attribute on a **Compartment** object must not be defined if the value of the `multi:isType` is "`true`". (References: Section 3.5.2 on page 11.)

**multi-20306** ☑ There may be at most one **ListOfCompartmentReferences** container object within a **Compartment** object. (References: Section 3.5.3 on page 12.)

**multi-20307** ☑ A **ListOfCompartmentReferences** object within a **Compartment** object is optional, but if present, must not be empty. (References: Section 3.5.3 on page 12.)

**multi-20308** ☑ A **ListOfCompartmentReferences** object may have the optional SBML core attributes `metaid` and `sboTerm`. No other attributes from the SBML Level 3 Core namespace or the Multi namespace are permitted on a **ListOfCompartmentReferences** object. (References: Section 3.5.3 on page 12.)

**multi-20309** ☑ Apart from the general `notes` and `annotation` subobjects permitted on all SBML objects, a **ListOfCompartmentReferences** container object may only contain **CompartmentReference** objects. (References: Section 3.5.3 on page 12.)

### Rules for SpeciesType objects

**multi-20401** ☑ A **SpeciesType** object may have the optional SBML Level 3 Core attributes `metaid` and `sboTerm`. No other attributes from the SBML Level 3 Core namespace are permitted on a **SpeciesType** object. (References: Section 3.8 on page 12).

**multi-20402** ☑ A **SpeciesType** object may have the optional SBML Level 3 Core subobjects for `notes` and `annotation`. No other elements from the SBML Level 3 Core namespace are permitted on a **SpeciesType** object. (References: Section 3.8 on page 12).

**multi-20403** ☑ A **SpeciesType** object must have the required attribute `multi:id`, and may have the optional attributes `multi:name` and `multi:compartment`. No other attributes from the Multi namespace are permitted on a **SpeciesType** object. (References: Section 3.8 on page 12.)

**multi-20404** ☑ The value of the `multi:compartment` attribute, if set on a given **SpeciesType** object, must be the value of an `id` attribute on an existing **Compartment** object in the `SId` namespace of the parent **Model** object. (References: Section 3.8.2 on page 13.)

**multi-20405** ☑ The various **ListOf**__ subobjects within a **SpeciesType** object are optional, but if present, these container objects must not be empty. Specifically, if any of the following classes of objects are present with a **SpeciesType** object, it must not be empty: **ListOfSpeciesFeatureTypes**, **ListOfSpeciesTypeInstances**, **ListOfSpeciesTypeComponentIndexes** and **ListOfInSpeciesTypeBonds**. (References: Section 3.8 on page 12.)

**multi-20406** ☑ There may be at most one **ListOfSpeciesFeatureTypes** container object within a **SpeciesType** object. (References: Section 3.8 on page 12.)

**multi-20407** ☑ Apart from the general `notes` and `annotation` subobjects permitted on all SBML objects, a **ListOfSpeciesFeatureTypes** container object may only contain **SpeciesFeatureType** objects. (References: Section 3.8.3 on page 13.)

**multi-20408** ☑ A **ListOfSpeciesFeatureTypes** object may have the optional SBML core attributes `metaid` and `sboTerm`. No other attributes from the SBML Level 3 Core namespace or the Multi namespace are permitted on a **ListOfSpeciesFeatureTypes** object. (References: Section 3.8.3 on page 13.)

**multi-20409** ☑ There may be at most one **ListOfSpeciesTypeInstances** container object within a **SpeciesType** object. (References: Section 3.8 on page 12.)

**multi-20410** ☑ Apart from the general `notes` and `annotation` subobjects permitted on all SBML objects, a **ListOfSpeciesTypeInstances** container object may only contain **SpeciesTypeInstance** objects. (References: Section 3.8.4 on page 13.)

**multi-20411** ☑ A **ListOfSpeciesTypeInstances** object may have the optional SBML core attributes `metaid` and `sboTerm`. No other attributes from the SBML Level 3 Core namespace or the Multi namespace are permitted on a **ListOfSpeciesTypeInstances**. (References: Section 3.8.4 on page 13.)

**multi-20412** ☑ There may be at most one **ListOfSpeciesTypeComponentIndexes** container object within a **SpeciesType** object. (References: Section 3.8 on page 12.)

**multi-20413** ☑ Apart from the general `notes` and `annotation` subobjects permitted on all SBML objects, a **ListOfSpeciesTypeComponentIndexes** container object may only contain **SpeciesTypeComponentIndex** objects. (References: Section 3.8.6 on page 14.)

**multi-20414** ☑ A **ListOfSpeciesTypeComponentIndexes** object may have the optional SBML core attributes `metaid` and `sboTerm`. No other attributes from the SBML Level 3 Core namespace or the Multi namespace are permitted on a **ListOfSpeciesTypeComponentIndexes** object. (References: Section 3.8.6 on page 14.)

**multi-20415** ☑ There may be at most one **ListOfInSpeciesTypeBonds** container object within a **SpeciesType** object. (References: Section 3.8 on page 12.)

**multi-20416** ☑ Apart from the general `notes` and `annotation` subobjects permitted on all SBML objects, a **ListOfInSpeciesTypeBonds** container object may only contain **InSpeciesTypeBond** objects. (References: Section 3.8.5 on page 14.)

**multi-20417** ☑ A **ListOfInSpeciesTypeBonds** object may have the optional SBML core attributes `metaid` and `sboTerm`. No other attributes from the SBML Level 3 Core namespace or the Multi namespace are permitted on a **ListOfInSpeciesTypeBonds** object. (References: Section 3.8.5 on page 14.)

### Rules for BindingSiteSpeciesType objects

**multi-20501** ☑ A **BindingSiteSpeciesType** object is not permitted to have any **ListOfSpeciesTypeInstances** subobject. (References: Section 3.8.7 on page 14).

### Rules for SpeciesFeatureType objects

**multi-20601** ☑ A **SpeciesFeatureType** object may have the optional SBML Level 3 Core attributes `metaid` and `sboTerm`. No other attributes from the SBML Level 3 Core namespace are permitted on a **SpeciesFeatureType** object. (References: Section 3.9 on page 14).

**multi-20602** ☑ A **SpeciesFeatureType** object may have the optional SBML Level 3 Core subobjects for `notes` and `annotation`. No other elements from the SBML Level 3 Core namespace are permitted on a **SpeciesFeatureType** object. (References: Section 3.9 on page 14).

**multi-20603** ☑ A **SpeciesFeatureType** object must have the required attributes `multi:id` and `multi:occur`, and may have the optional attribute `multi:name`. No other attributes from the Multi namespace are permitted on a **SpeciesFeatureType** object. (References: Section 3.9 on page 14.)

**multi-20604** ☑ The value of the `multi:occur` attribute on a given **SpeciesFeatureType** object must conform to the syntax of the SBML data type `positiveInteger`. (References: Section 3.9.2 on page 14.)

**multi-20605** ☑ One **ListOfPossibleSpeciesFeatureValues** subobject in a **SpeciesFeatureType** object is required. (References: Section 3.9.3 on page 14.)

**multi-20606** ☑ A **ListOfPossibleSpeciesFeatureValues** object may have the optional SBML core attributes `metaid` and `sboTerm`. No other attributes from the SBML Level 3 Core namespace or the Multi namespace are permitted on a **ListOfPossibleSpeciesFeatureValues** object. (References: Section 3.9.3 on page 14.)

**multi-20607** ☑ Apart from the general `notes` and `annotation` subobjects permitted on all SBML objects, a **ListOfPossibleSpeciesFeatureValues** container object may only contain **PossibleSpeciesFeatureValue** objects. (References: Section 3.9.3 on page 14.)

**multi-20608** ☑ A **ListOfPossibleSpeciesFeatureValues** object must not be empty. (References: Section 3.9.3 on page 14.)

### Rules for PossibleSpeciesFeatureValue objects

**multi-20701** ☑ A **PossibleSpeciesFeatureValue** object may have the optional SBML Level 3 Core attributes `metaid` and `sboTerm`. No other attributes from the SBML Level 3 Core namespace are permitted on a **PossibleSpeciesFeatureValue** object. (References: Section 3.10 on page 15).

**multi-20702** ☑ A **PossibleSpeciesFeatureValue** object may have the optional SBML Level 3 Core subobjects for `notes` and `annotation`. No other elements from the SBML Level 3 Core namespace are permitted on a **PossibleSpeciesFeatureValue** object. (References: Section 3.10 on page 15).

**multi-20703** ☑ A **PossibleSpeciesFeatureValue** object must have the required attribute `multi:id`, and may have the optional attributes `multi:name` and `multi:numericValue`. No other attributes from the Multi namespace are permitted on a **PossibleSpeciesFeatureValue** object. (References: Section 3.10 on page 15.)

**multi-20704** ☑ The value of the `multi:numericValue` attribute on a given **PossibleSpeciesFeatureValue** object must be the identifier of a **Parameter** object defined in the same **Model** object. (References: Section 3.10.2 on page 15.)

### Rules for SpeciesTypeInstance objects

**multi-20801** ☑ A **SpeciesTypeInstance** object may have the optional SBML Level 3 Core attributes `metaid` and `sboTerm`. No other attributes from the SBML Level 3 Core namespace are permitted on a **SpeciesTypeInstance** object. (References: Section 3.11 on page 16).

**multi-20802** ☑ A **SpeciesTypeInstance** object may have the optional SBML Level 3 Core subobjects for `notes` and `annotation`. No other elements from the SBML Level 3 Core namespace are permitted on a **SpeciesTypeInstance** object. (References: Section 3.11 on page 16).

**multi-20803** ☑ A **SpeciesTypeInstance** object must have the required attributes `multi:id` and `multi:speciesType`, and may have the optional attributes `multi:name` and `multi:compartmentReference`. No other attributes from the Multi namespace are permitted on a **SpeciesTypeInstance** object. (References: Section 3.11 on page 16.)

**multi-20805** ☑ The value of the `multi:speciesType` attribute on a given **SpeciesTypeInstance** object must be the identifier of a **SpeciesType** object defined in the same **Model** object. (References: Section 3.11.2 on page 16.)

**multi-20806** ☑ The value of the `multi:compartmentReference` attribute, if present on a given **SpeciesTypeInstance** object, must be the identifier of a **CompartmentReference** object defined in the same **Model** object. (References: Section 3.11.3 on page 16.)

### Rules for SpeciesTypeComponentIndex objects

**multi-20901** ☑ A **SpeciesTypeComponentIndex** object may have the optional SBML Level 3 Core attributes `metaid` and `sboTerm`. No other attributes from the SBML Level 3 Core namespace are permitted on a **SpeciesTypeComponentIndex** object. (References: Section 3.12 on page 17).

**multi-20902** ☑ A **SpeciesTypeComponentIndex** object may have the optional SBML Level 3 Core subobjects for `notes` and `annotation`. No other elements from the SBML Level 3 Core namespace are permitted on a **SpeciesTypeComponentIndex** object. (References: Section 3.12 on page 17).

**multi-20903** ☑ A **SpeciesTypeComponentIndex** object must have the required attributes `multi:id` and `multi:compartment`, and may have the optional attributes `multi:name` and `multi:identifyingParent`. No other attributes from the Multi namespace are permitted on a **SpeciesTypeComponentIndex** object. (References: Section 3.12 on page 17.)

**multi-20904** ☑ The value of the `multi:component` attribute on a given **SpeciesTypeComponentIndex** object must be the identifier of a **SpeciesTypeInstance** object, or a **SpeciesTypeComponentIndex** object under the **SpeciesType** object that this **SpeciesTypeComponentIndex** object belongs to, or the **SpeciesType** object itself. (References: Section 3.12.2 on page 17.)

**multi-20907** ☑ The value of the `multi:identifyingParent` attribute on a given **SpeciesTypeComponentIndex** object must be the identifier of a `component` object under the **SpeciesType** object that this **SpeciesTypeComponentIndex** object belongs to. A `component` object can be an object of **SpeciesTypeInstance**, **SpeciesTypeComponentIndex** or **SpeciesType**. (References: Section 3.12.3 on page 18.)

### Rules for InSpeciesTypeBond objects

**multi-21101** ☑ An **InSpeciesTypeBond** object may have the optional SBML Level 3 Core attributes `metaid` and `sboTerm`. No other attributes from the SBML Level 3 Core namespace are permitted on an **InSpeciesTypeBond** object. (References: Section 3.13 on page 19).

**multi-21102** ☑ An **InSpeciesTypeBond** object may have the optional SBML Level 3 Core subobjects for `notes` and `annotation`. No other elements from the SBML Level 3 Core namespace are permitted on an **InSpeciesTypeBond** object. (References: Section 3.13 on page 19).

**multi-21103** ☑ An **InSpeciesTypeBond** object must have the required attributes, `multi:bindingSite1` and `multi:bindingSite2`, and may have the optional attributes, `multi:id` and `multi:name`. No other attributes from the Multi namespace are permitted on an **InSpeciesTypeBond** object. (References: Section 3.13 on page 19.)

**multi-21104** ☑ The value of the `multi:bindingSite1` attribute on a given **InSpeciesTypeBond** object must be the identifier of a **SpeciesTypeInstance** object or **SpeciesTypeComponentIndex** which ultimately reference a object of **BindingSiteSpeciesType**. (References: Section 3.13.2 on page 20.)

**multi-21105** ☑ The value of the `multi:bindingSite2` attribute on a given **InSpeciesTypeBond** object must be the identifier of a **SpeciesTypeInstance** object or **SpeciesTypeComponentIndex** which ultimately reference a object of **BindingSiteSpeciesType**. (References: Section 3.13.2 on page 20.)

**multi-21106** ☑ The `multi:bindingSite1` and `multi:bindingSite2` attributes must not reference the same **BindingSiteSpeciesType** object. (References: Section 3.13.2 on page 20.)

### Rules for extended Species objects

**multi-21201** ☑ A **Species** object may have the optional attribute, `multi:speciesType`. No other attributes from the Multi namespace are permitted on a **Species** object. (References: Section 3.14 on page 22.)

**multi-21202** ☑ The value of a `multi:speciesType` attribute, if present on a **Species** object, must be the identifier of a **SpeciesType** object. (References: Section 3.15.1 on page 22.)

**multi-21203** ☑ Two **ListOf__** subobjects with a **Species** object are optional, but if present, these container object must not be empty. Specifically, if any of the following two classes of objects are present on the **Species** object, it must not be empty: **ListOfOutwardBindingSites** and **ListOfSpeciesFeatures**. (References: Section 3.14 on page 22.)

**multi-21204** ☑ A **ListOfOutwardBindingSites** object may have the optional SBML core attributes `metaid` and `sboTerm`. No other attributes from the SBML Level 3 Core namespace or the Multi namespace are permitted on a **ListOfOutwardBindingSites** object. (References: Section 3.15.2 on page 22.)

**multi-21205** ☑ Apart from the general `notes` and `annotation` subobjects permitted on all SBML objects, a **ListOfOutwardBindingSites** container object may only contain **OutwardBindingSite** objects. (References: Section 3.15.2 on page 22.)

**multi-21206** ☑ A **ListOfSpeciesFeatures** object may have the optional SBML core attributes `metaid` and `sboTerm`. No other attributes from the SBML Level 3 Core namespace or the Multi namespace are permitted on a **ListOfSpeciesFeatures** object. (References: Section 3.15.3 on page 23.)

**multi-21207** ☑ A **SubListOfSpeciesFeatures** object may have the optional attributes `multi:id`, `multi:name`, `multi:component`, and the required attribute `multi:relation`. No other attributes from the Multi namespace are permitted on a **SubListOfSpeciesFeatures** object. (References: Section 3.17 on page 25.)

**multi-21208** ☑ The value of the `multi:relation` attribute, if presented on a **SubListOfSpeciesFeatures** object, must conform to the syntax of the Multi data type **`Relation`**. (References: Section 3.17.2 on page 25.)

**multi-21209** ☑ Apart from the general `notes` and `annotation` subobjects permitted on all SBML objects, a **ListOfSpeciesFeatures** container object may only contain **SpeciesFeature** and/or **SubListOfSpeciesFeatures** objects. (References: Section 3.15.3 on page 23.)

**multi-21210** ☑ A **SubListOfSpeciesFeatures** object may have the optional SBML core attributes `metaid` and `sboTerm`. No other attributes from the SBML Level 3 Core namespace are permitted on a **SubListOfSpeciesFeatures** object. (References: Section 3.17 on page 25.)

**multi-21211** ☑ Apart from the general `notes` and `annotation` subobjects permitted on all SBML objects, a **SubListOfSpeciesFeatures** container object may only contain **SpeciesFeature** objects. (References: Section 3.17 on page 25.)

**multi-21212** ☑ The value of the `multi:component` attribute on a given **SubListOfSpeciesFeatures** object must be the identifier of an object of **SpeciesTypeInstance**, **SpeciesTypeComponentIndex** or **SpeciesType** which contains the **SpeciesFeature** objects in this `subListOfSpeciesFeatures`. (References: Section 3.17.3 on page 25.)

**multi-21213** ☑ A `species` must have its `speciesType` attribute defined when it has a `listOfOutwardBindingSites`. (References: Section 3.14 on page 22.)

**multi-21214** ☑ A `species` must have its `speciesType` attribute defined when it has a `listOfSpeciesFeatures`. (References: Section 3.14 on page 22.)

**multi-21215** ☑ The `relation` attribute of a `subListOfSpeciesFeatures` can only have the value "`and`" if any `speciesFeature` involved references a `speciesFeatureType` with "`occur > 1`". (References: Section 3.17.2 on page 25.)

**multi-21216** ☑ A **SubListOfSpeciesFeatures** object must have at least two `speciesFeature`s. (References: Section 3.17 on page 25.)

### Rules for OutwardBindingSite objects

**multi-21301** ☑ An **OutwardBindingSite** object may have the optional SBML Level 3 Core attributes `metaid` and `sboTerm`. No other attributes from the SBML Level 3 Core namespace are permitted on an **OutwardBindingSite** object. (References: Section 3.16 on page 23).

**multi-21302** ☑ An **OutwardBindingSite** object may have the optional SBML Level 3 Core subobjects for `notes` and `annotation`. No other elements from the SBML Level 3 Core namespace are permitted on an **OutwardBindingSite** object. (References: Section 3.16 on page 23).

**multi-21303** ☑ An **OutwardBindingSite** object must have the required attributes, `multi:bindingStatus` and `multi:component`, and may have the optional attributes `multi:id` and `multi:name`. No other attributes from the Multi namespace are permitted on an **OutwardBindingSite** object. (References: Section 3.16 on page 23.)

**multi-21304** ☑ The value of the `multi:bindingStatus` attribute on a given **OutwardBindingSite** object must conform to the syntax of the Multi data type `BindingStatus`. (References: Section 3.16.2 on page 23.)

**multi-21305** ☑ The value of the `multi:component` attribute on a given **OutwardBindingSite** object must be the identifier of an object of **SpeciesTypeInstance**, **SpeciesTypeComponentIndex** or **SpeciesType** which ultimately reference an object of **BindingSiteSpeciesType**. (References: Section 3.16.3 on page 23.)

**multi-21306** ☑ An `outwardBindingSite` cannot be a binding site referenced by any `inSpeciesTypeBond` in the `species`. (References: Section 3.16.3 on page 23.)

### Rules for SpeciesFeature objects

**multi-21401** ☑ A **SpeciesFeature** object may have the optional SBML Level 3 Core attributes `metaid` and `sboTerm`. No other attributes from the SBML Level 3 Core namespace are permitted on a **SpeciesFeature** object. (References: Section 3.18 on page 25).

**multi-21402** ☑ A **SpeciesFeature** object may have the optional SBML Level 3 Core subobjects for `notes` and `annotation`. No other elements from the SBML Level 3 Core namespace are permitted on a **SpeciesFeature** object. (References: Section 3.18 on page 25).

**multi-21403** ☑ A **SpeciesFeature** object must have the required attributes, `multi:speciesFeatureType` and `multi:occur`, and may have the optional attributes, `multi:id`, `multi:name`, and `multi:component`. No other attributes from the Multi namespace are permitted on a **SpeciesFeature** object. (References: Section 3.18 on page 25.)

**multi-21404** ☑ The value of the `multi:speciesFeatureType` attribute on a given **SpeciesFeature** object must be the identifier of a **SpeciesFeatureType** object which is in the **SpeciesType** object referenced by the **Species** object containing this **SpeciesFeature** object. (References: Section 3.18.2 on page 26.)

**multi-21405** ☑ The value of the `multi:occur` attribute on a given **SpeciesFeature** object must conform to the syntax of the SBML data type `positiveInteger`. The value of the `multi:occur` attribute must not be larger than that of the `multi:occur` attribute of the **SpeciesFeatureType** object referenced by this **SpeciesFeature** object. (References: Section 3.18.3 on page 26.)

**multi-21406** ☑ The value of the `multi:component` attribute on a given **SpeciesFeature** object must be the identifier of an object of **SpeciesTypeInstance**, **SpeciesTypeComponentIndex** or **SpeciesType** which contains this **SpeciesFeature** object. (References: Section 3.18.4 on page 27.)

**multi-21407** ☑ One and only one **ListOfSpeciesFeatureValues** subobject within a **SpeciesFeature** object is required. (References: Section 3.18.5 on page 27.)

**multi-21408** ☑ A **ListOfSpeciesFeatureValues** object must not be empty. (References: Section 3.18.5 on page 27.)

**multi-21409** ☑ A **ListOfSpeciesFeatureValues** object may have the optional SBML core attributes `metaid` and `sboTerm`. No other attributes from the SBML Level 3 Core namespace or the Multi namespace are permitted on a **ListOfSpeciesFeatureValues** object. (References: Section 3.18.5 on page 27.)

**multi-21410** ☑ Apart from the general `notes` and `annotation` subobjects permitted on all SBML objects, a **ListOfSpeciesFeatureValues** container object may only contain **SpeciesFeatureValue** objects. (References: Section 3.18.5 on page 27.)

### Rules for SpeciesFeatureValue objects

**multi-21501** ☑ A **SpeciesFeatureValue** object may have the optional SBML Level 3 Core attributes `metaid` and `sboTerm`. No other attributes from the SBML Level 3 Core namespace are permitted on a **SpeciesFeatureValue** object. (References: Section 3.18.6 on page 27).

**multi-21502** ☑ A **SpeciesFeatureValue** object may have the optional SBML Level 3 Core subobjects for `notes` and `annotation`. No other elements from the SBML Level 3 Core namespace are permitted on a **SpeciesFeatureValue** object. (References: Section 3.18.6 on page 27).

**multi-21503** ☑ A **SpeciesFeatureValue** object must have the required attribute `multi:value`. No other attributes from the Multi namespace are permitted on a **SpeciesFeatureValue** object. (References: Section 3.18.6 on page 27.)

**multi-21504** ☑ The value of the `multi:value` attribute on a given **SpeciesFeatureValue** object must be the identifier of a **PossibleSpeciesFeatureValue** object defined in the **SpeciesFeatureType** object referenced by the **SpeciesFeature** object containing this **SpeciesFeatureValue** object. (References: Section 3.18.6 on page 27.)

### Rules for IntraSpeciesReaction objects

**multi-21601** ☑ An **IntraSpeciesReaction** object may have the optional SBML Level 3 Core attributes `metaid` and `sboTerm`. No other attributes from the SBML Level 3 Core namespace and the Multi namespace are permitted on an **IntraSpeciesReaction** object. (References: Section 3.21 on page 30).

**multi-21602** ☑ An **IntraSpeciesReaction** object may have the optional SBML Level 3 Core subobjects for `notes` and `annotation`. No other elements from the SBML Level 3 Core namespace are permitted on an **IntraSpeciesReaction** object. (References: Section 3.21 on page 30).

### Rules for extended SimpleSpeciesReference objects

**multi-21701** ☑ An extended **SimpleSpeciesReference** object may have the optional attribute, `multi:compartmentReference`. No other attributes from the Multi namespace are permitted on a **SimpleSpeciesReference** object. (References: Section 3.22 on page 30.)

**multi-21702** ☑ The value of a `multi:compartmentReference` attribute, if present on a **SimpleSpeciesReference** object, must be the identifier of a **CompartmentReference** object. (References: Section 3.22 on page 30.)

### Rules for extended SpeciesReference objects

**multi-21801** ☑ A **ListOfSpeciesTypeComponentMapsInProduct** object within an extended **SpeciesReference** object is optional, but if present, must not be empty. (References: Section 3.23.1 on page 32.)

**multi-21802** ☑ A **ListOfSpeciesTypeComponentMapsInProduct** object may have the optional SBML core attributes `metaid` and `sboTerm`. No other attributes from the SBML Level 3 Core namespace or the Multi namespace are permitted on a **ListOfSpeciesTypeComponentMapsInProduct** object. (References: Section 3.23.1 on page 32.)

**multi-21803** ☑ Apart from the general `notes` and `annotation` subobjects permitted on all SBML objects, a **ListOfSpeciesTypeComponentMapsInProduct** container object may only contain **SpeciesTypeComponentMapInProduct** objects. (References: Section 3.23.1 on page 32.)

### Rules for SpeciesTypeComponentMapInProduct objects

**multi-21901** ☑ A **SpeciesTypeComponentMapInProduct** object may have the optional SBML Level 3 Core attributes `metaid` and `sboTerm`. No other attributes from the SBML Level 3 Core namespace are permitted on a **SpeciesTypeComponentMapInProduct** object. (References: Section 3.24 on page 32).

**multi-21902** ☑ A **SpeciesTypeComponentMapInProduct** object may have the optional SBML Level 3 Core subobjects for `notes` and `annotation`. No other elements from the SBML Level 3 Core namespace are permitted on a **SpeciesTypeComponentMapInProduct** object. (References: Section 3.24 on page 32).

**multi-21903** ☑ A **SpeciesTypeComponentMapInProduct** object must have the required attributes `multi:reactant`, `multi:reactantComponent`, and `multi:productComponent`, and may have the optional attributes `multi:id` and `multi:name`. No other attributes from the Multi namespace are permitted on a **SpeciesTypeComponentMapInProduct** object. (References: Section 3.24 on page 32.)

**multi-21904** ☑ The value of the `multi:reactant` attribute on a given **SpeciesTypeComponentMapInProduct** object must be the identifier of a reactant **SpeciesReference** object within a reaction. (References: Section 3.24.2 on page 33.)

**multi-21905** ☑ The value of the `multi:reactantComponent` attribute on a given **SpeciesTypeComponentMapInProduct** object must be the identifier of an object of **SpeciesTypeInstance**, **SpeciesTypeComponentIndex** or **SpeciesType**. (References: Section 3.24.3 on page 33.)

**multi-21906** ☑ The value of the `multi:productComponent` attribute on a given **SpeciesTypeComponentMapInProduct** object must be the identifier of an object of **SpeciesTypeInstance**, **SpeciesTypeComponentIndex** or **SpeciesType**. (References: Section 3.24.4 on page 33.)

### Rules for CompartmentReference objects

**multi-22001** ☑ A **CompartmentReference** object may have the optional SBML Level 3 Core attributes `metaid` and `sboTerm`. No other attributes from the SBML Level 3 Core namespace are permitted on a **CompartmentReference** object. (References: Section 3.6 on page 12).

**multi-22002** ☑ A **CompartmentReference** object may have the optional SBML Level 3 Core subobjects for `notes` and `annotation`. No other elements from the SBML Level 3 Core namespace are permitted on a **CompartmentReference** object. (References: Section 3.6 on page 12).

**multi-22003** ☑ A **CompartmentReference** object must have the required attribute `multi:compartment`, and may have the optional attributes `multi:id` and `multi:name`. No other attributes from the Multi namespace are permitted on a **CompartmentReference** object. (References: Section 3.6 on page 12.)

**multi-22004** ☑ The value of the `multi:compartment` attribute must be the value of an `id` attribute on an existing **Compartment** object in the `SId` namespace of the parent **Model**. (References: Section 3.6 on page 12.)

**multi-22005** ☑ If some or all **CompartmentReference** objects within a **ListOfCompartmentReferences** object reference the same **Compartment** object, those `compartmentReference`s are required to have its `multi:id` attribute defined. (References: Section 3.6.1 on page 12.)

**multi-22006** ☑ A `compartmentReference` cannot reference a `compartment` that directly or indirectly contains the `compartmentReference`. (References: Section 3.6.2 on page 12.)

## Acknowledgments

This work was supported by the Intramural Research Program of the US National Institute of Allergy and Infectious Diseases of the National Institutes of Health.

We sincerely thank all the contributors and their funding agencies. L.P.S., S.M.K, N.R., A.D., F.B. and M.H. were supported by the National Institute of General Medical Sciences (NIGMS, US) grant R01 GM070923. In addition, A.D. was supported by the DZIF (German Center for Infection Research) and by infrastructural funding from the *Deutsche Forschungsgemeinschaft* (DFG, German Research Foundation), Cluster of Excellence EXC 2124 Controlling Microbes to Fight Infections; and F.B. was supported by the Bundesministerium für Bildung und Forschung (BMBF, DE) grant de.NBI ModSim1, 031L0104A. M.L.B. was supported by NIH (US) grant P41 GM103313 and R24 GM134211. J.F. and J.J.T. were supported by National Institutes of Health (NIH, US) grant P41 GM103712 to the National Center for Multiscale Modeling of Biological Systems (MMBioS). W.S.H was supported by the National Institute of General Medical Sciences (NIGMS, US) grant R01 GM111510.
